# Shifts in the coral microbiome in response to *in situ* experimental deoxygenation

**DOI:** 10.1128/aem.00577-23

**Published:** 2023-11-02

**Authors:** Rachel D. Howard, Monica D. Schul, Lucia M. Rodriguez Bravo, Andrew H. Altieri, Julie L. Meyer

**Affiliations:** 1 Department of Soil, Water, and Ecosystem Sciences, University of Florida, Gainesville, Florida, USA; 2 Smithsonian Tropical Research Institute, Balboa, Ancon, Panama; 3 Red Sea Research Center, King Abdullah University of Science and Technology, Thuwal, Saudi Arabia; 4 Department of Environmental Engineering Sciences, University of Florida, Gainesville, Florida, USA; University of Delaware, Lewes, Delaware, USA

**Keywords:** coral, microbiome, hypoxia, oxygen, *Agaricia lamarcki*, *Siderastrea siderea*, Panama

## Abstract

Global climate change impacts marine ecosystems through rising surface temperatures, ocean acidification, and deoxygenation. While the response of the coral holobiont to the first two effects has been relatively well studied, less is known about the response of the coral microbiome to deoxygenation. In this study, we investigated the response of the microbiome to hypoxia in two coral species that differ in their tolerance to hypoxia. We conducted *in situ* oxygen manipulations on a coral reef in Bahía Almirante on the Caribbean coast of Panama, which has previously experienced documented episodes of hypoxia. Naïve coral colonies (previously unexposed to hypoxia) of *Siderastrea siderea* and *Agaricia lamarcki* were transplanted to a reef and either enclosed in chambers that created hypoxic conditions or left at ambient oxygen levels. We collected samples of surface mucus and tissue after 48 hours of exposure and characterized the microbiome by sequencing 16S rRNA genes. We found that the microbiomes of the two coral species were distinct from one another and remained so after exhibiting similar shifts in microbiome composition in response to hypoxia. There was an increase in both abundance and number of taxa of anaerobic microbes after exposure to hypoxia. Some of these taxa may play beneficial roles in the coral holobiont by detoxifying the surrounding environment during hypoxic stress or may represent opportunists exploiting host stress. This work describes the first characterization of the coral microbiome under hypoxia and is an initial step toward identifying potential beneficial bacteria for corals facing this environmental stressor.

**IMPORTANCE:**

Marine hypoxia is a threat for corals but has remained understudied in tropical regions where coral reefs are abundant. Though microbial symbioses can alleviate the effects of ecological stress, we do not yet understand the taxonomic or functional response of the coral microbiome to hypoxia. In this study, we experimentally lowered oxygen levels around *Siderastrea siderea* and *Agaricia lamarcki* colonies *in situ* to observe changes in the coral microbiome in response to deoxygenation. Our results show that hypoxia triggers a stochastic change of the microbiome overall, with some bacterial families changing deterministically after just 48 hours of exposure. These families represent an increase in anaerobic and opportunistic taxa in the microbiomes of both coral species. Thus, marine deoxygenation destabilizes the coral microbiome and increases bacterial opportunism. This work provides novel and fundamental knowledge of the microbial response in coral during hypoxia and may provide insight into holobiont function during stress.

## INTRODUCTION

Marine deoxygenation is a devastating and global threat to oceanic and coastal ecosystems, with ecological, evolutionary, and social repercussions comparable to other major anthropogenic threats including warming and ocean acidification (1 – 3). While previous work has established hypoxia as a widespread threat to temperate marine ecosystems (2 – 5), it has only recently garnered attention in tropical marine systems as a cause of mass mortality that reduces biodiversity and productivity (6). Many marine species globally are already in decline due to oxygen levels at or below critical oxygen thresholds (7), and decreased oxygen availability will likely be responsible for large shifts in ecosystem structures (8). Localized coastal hypoxia in tropical and subtropical waters has recently become a substantial threat to corals (9). Prolonged exposure to hypoxia can have adverse effects on coral health and resiliency including bleaching, disease, and mortality (6, 10 – 12).

Though prolonged exposure to hypoxia will ultimately lead to death, corals and other reef-associated organisms may have an innate tolerance to periodic deoxygenation (6, 7, 13 – 16). Corals are able to actively stir water at their surface microenvironment with their epidermal cilia, which can transport oxygen and support molecular diffusion at the host surface (17). Corals undergo natural diel shifts in oxygen concentrations within their surface microenvironment (18 – 20). When sunlight is available in the photic zone during the day, oxygen produced by *Symbiodiniaceae* saturates the coral surface (18, 19). At night, coral holobiont respiration uses the free oxygen, creating a hypoxic microenvironment on the coral surface until sunlight triggers photosynthesis (18, 19). These diel changes in oxygen concentration can occur in the matter of minutes (20), yet the coral remains mostly undisturbed.

Corals may also exhibit some hypoxia tolerance during the periodic macroscale oxygen depletion that can occur naturally on reefs. These shifts in dissolved oxygen concentrations occur because of unusual weather patterns (21 – 23), reef geomorphology (21, 24 – 26), isolation of reefs during diel tidal cycles (24, 27), coral spawn slicks (22, 28), or other elements that reduce water column mixing and exchange with the open ocean (29). However, these natural occurrences of deoxygenation are exacerbated by eutrophication and climate change, intensifying the overall severity and duration of hypoxic events globally (1, 4, 9, 30, 31). With over 13% of the world’s coral reefs at an elevated risk for deoxygenation (6), understanding the response of corals to hypoxia and implementing mitigation strategies to reefs is critical.

The coral microbiome is a source of resilience for environmental stressors including warming (32, 33) and may play a similarly important role for hypoxia. Members of the microbiome fill a variety of functional roles within the coral host (10, 34 – 36), including nutrient cycling within the holobiont (35 – 37), nitrogen fixation (35, 36, 38), and pathogen resistance (35 – 37, 39). If there is flexibility of microbial species in response to dynamic oxygen conditions, this could contribute to the observed ability of coral hosts to withstand exposure to hypoxic conditions. Here, we experimentally induced hypoxic conditions with an *in situ* reef experiment to test how the microbiomes of the hypoxia-resistant massive starlet coral (*Siderastrea siderea*) (40) and the hypoxia-sensitive whitestar sheet coral (*Agaricia lamarcki*) (6, 40) responded to hypoxia.

## MATERIALS AND METHODS

### Site description

Bahiá Almirante in Bocas del Toro, Panama, is a large, semi-enclosed tropical embayment of 450 km^2^ (6) and is home to many shallow-water (<25 m) coral reefs (41, 42). This basin on the Caribbean coast shares many features with temperate estuaries that experience bouts of hypoxia, including reduced exchange with the open ocean, seasonal cycles of low wind energy and high temperatures, and a watershed delivering excess nutrients from agricultural run-off and untreated sewage (41, 43). Because of these conditions, Bahiá Almirante has experienced patches of hypoxic stress, with documented occurrences in 2010 and 2017 that caused extensive coral bleaching and necrosis in other marine invertebrates (6, 40). Due to these periodic hypoxic events, Bahiá Almirante and its coral reefs are ideal study sites for assessing the response of coral health and resilience to hypoxia. We chose massive starlet coral (*Siderastrea siderea*) and whitestar sheet coral (*Agaricia lamarcki*) as our study species because they are two of the predominant coral species in the region and exhibited strikingly different responses to prior hypoxia events, with *S. siderea* persisting at hypoxic sites (40) and *A. lamarcki* suffering near total mortality (6, 40).

### *In situ* oxygen manipulation

**Fig 1:**
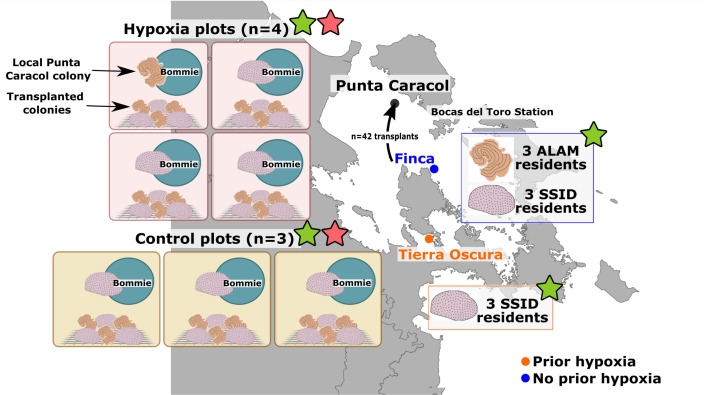
Map of experimental sites in Bahía Almirante, Bocas del Toro, Panama. Resident corals were sampled from Tierra Oscura (TO) and Finca (F) to test for site variation in the microbiome. Corals from Finca were transplanted to Punta Caracol for oxygen manipulation experiments (control plots, hypoxic plots). Each of the seven plots contained a mixed species bommie with a local Punta Caracol colony attached. Three transplanted *S. siderea* and *A. lamarcki* colonies were also placed in each plot by fastening the colonies to a mesh rack. Samples designated with pink stars were used in all analyses. Samples designated with green stars were used in Analysis of Compositions of Microbiomes (ANCOM).

To test the response of coral microbiomes to hypoxic stress, we conducted a field experiment in which we manipulated oxygen with benthic incubation chambers. The experiment was conducted at Punta Caracol, in the vicinity of areas with documented mortality associated with hypoxia (Fig. 1) (40, 44). Seven 60 × 60 cm plots were established and a miniDOT dissolved oxygen logger (Precision Measurement Engineering, Vista, CA) in each plot recorded oxygen concentration and temperature at 10-minute intervals. Four randomly selected plots were assigned to the hypoxia treatment, and the remaining three served as control plots (Fig. 1). Four-sided benthic incubation chambers made of greenhouse-grade plastic were used to locally reduce oxygen concentrations. The chambers were open at the bottom, with 15 cm flanges that were tucked into the sediment to better isolate the water within. A submersible aquarium pump was placed in each chamber to homogenize the water column and prevent stagnant water within. Control, oxygenated chambers employed the open plastic tent structure without the greenhouse-grade plastic.

Colonies of *A. lamarcki* and *S. siderea* (7–12 cm diameter) were collected at the Finca site from a depth of 5–10 m for transplantation to the experimental plots. Colonies were collected at least 2 m apart and likely represented independent genotypes. Coral colonies were transported in aerated seawater to Punta Caracol where they were randomly assigned to experimental plots. Each incubation chamber enclosed a local Punta Caracol bommie with a representative reef community that contained a mix of corals, sponges, and other benthic organisms that included either a *S. siderea* or *A. lamarcki* colony (Fig. 1). We transplanted three *S*. *siderea* and three *A. lamarcki* colonies to each plot by fastening the colonies to a mesh rack next to the bommie (Fig. 1). The experimental oxygen manipulation was conducted for 48 hours, at which time the coral surface microbiome was sampled.

### Coral microbiome sampling

In addition to coral colonies in the experimental plots, three colonies of *S. siderea* were sampled from Tierra Oscura where hypoxia has been previously documented and three colonies each of *A. lamarcki* and *S. siderea* were sampled from Finca where hypoxia has not been documented (Fig. 1) (40, 44, 45). Slurries of coral mucus/tissue were collected by agitation and suction of the coral surface with individual sterile needleless syringes. Syringes were transported in a cooler with ice to the lab, and mucus was allowed to settle in the syringes before expelling into a 2-mL cryovial with RNALater (Ambion, Austin, TX). Preserved samples were frozen until further processing at the University of Florida.

### V4 amplicon library preparation

Extraction of genomic DNA was performed with a DNeasy Powersoil Kit (Qiagen, Germantown, MD) according to the manufacturer’s instructions. The V4 region of the 16S rRNA gene was amplified in triplicate for each sample using the 515F (46) and 806RB (47) Earth Microbiome primers and thermocycler protocol (48) in 25 µL reactions containing Phusion High-fidelity Master Mix (New England Biolabs, Ipswich, MA), 0.25 µM of each primer, 3% dimethyl sulfoxide (as recommended by the manufacturer of the polymerase), and 2 µL of DNA template. Triplicate reactions were consolidated and cleaned with a MinElute PCR Purification Kit (Qiagen) and quantified with a DS-11 FX+ spectrophotometer (DeNovix, Wilmington, DE). One DNA extraction kit blank without the addition of any starting coral biomass was produced alongside regular DNA extractions and then amplified and sequenced using a unique barcode. One final pool containing 240 ng of each amplicon library was submitted to the University of Florida Interdisciplinary Center for Biotechnology Research (RRID:SCR_019152) for sequencing on an Illumina MiSeq with the 2 × 150bp v.2 cycle format.

### Analysis of V4 Amplicon libraries

Adapters and primers were removed from raw sequencing reads with cutadapt v. 1.8.1 (49). Further processing of amplicon libraries was completed in RStudio v. 1.1.456 with R v. 4.0.4. Quality filtering, error estimation, merging of reads, dereplication, removal of chimeras, and selection of amplicon sequence variants (ASVs) were performed with DADA2 v. 1.18.0 (50) using the filtering parameters: filterAndTrim {fnFs, filtFs, fnRs, truncLen = c(150,150), maxN = 0, maxEE = [c(2,2), truncQ = 2, rm.phix = TRUE, compress = TRUE, multithread = TRUE]}. Taxonomy was assigned to ASVs using the SILVA small subunit rRNA database v. 132 (51). The ASV and taxonomy tables were imported into phyloseq v. 1.34.0 (52) for analysis and visualization of microbial community structure. ASVs with a mean read count of less than five across all samples were removed from the analysis, and ASVs assigned as chloroplast, mitochondria, or eukaryote were removed from further analysis. Remaining ASVs labeled only as “Bacteria” were searched with BLASTn, and those matching mitochondrial sequences were removed from the analysis.

Variation in community composition was determined using the Aitchison distance of centered log-ratio transformed, zero-replaced read counts using CoDaSeq v. 0.99.6 (53) and visualized with principal component analysis. Principal component analysis of the Aitchison distance was performed with the package prcomp in R and plotted with ggplot2 v. 3.3.3 (54). Permutational Multivariate Analysis of Variance (PERMANOVA) with vegan v. 2.5-7 (55) was used to test for differences in community structure by treatment and coral species.

We also estimated beta diversity dispersion using the dissimilarity matrix by estimating the distance to a group’s centroid for each sample. This measure of multivariate dispersion was calculated using the betadisper function in vegan (55) and based on the treatment type (control plots, hypoxia plots) for both coral species. We examined beta diversity dispersion visually with a boxplot and tested differences in beta diversity dispersion between treatment types with ANOVA.

Original count data were used for the ANCOM statistics. For clarity, the nine coral microbiome samples collected at Tierra Oscura and Finca that were not part of the experimental plots were only included in the ANCOM figures, as they did not provide sufficient statistical power for additional analyses (Fig. 1). ANCOM (56) was used to identify microbial families that were differentially abundant across treatments, using an ANOVA significance level of 0.05 and removing families with zero counts in 90% or more of samples. Only families detected in at least 70% of samples were reported. Finally, indicspecies v. 1.7.9 (57) was used to identify differentially abundant ASVs amongst treatment types. The complete set of R scripts and metadata are available at github.com/meyermicrobiolab/Panama_Hypoxia.

## RESULTS

### Experimental deoxygenation

**Fig 2:**
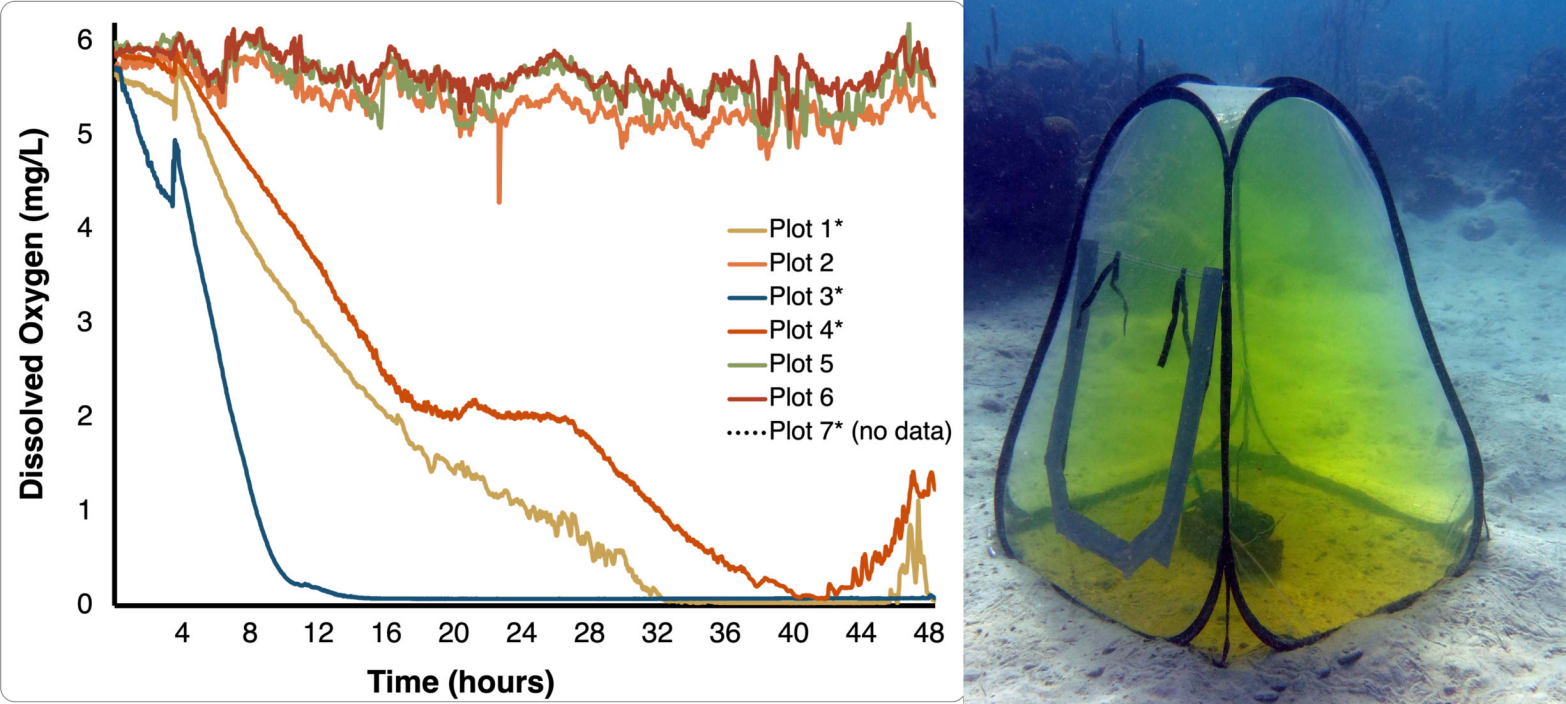
(**A**) Dissolved oxygen concentrations (mg/L) in the hypoxic and control plots over 48 hours. Tent 3 became hypoxic rapidly and stayed hypoxic for the duration of the experiment. (**B**) An example of the greenhouse chamber used to simulate natural hypoxia in the marine environment. Fluorescein dye was used before trials to ensure the chambers could be secured with minimal flow-through and leaks.

Dissolved oxygen (DO) concentrations (mg/L) in the control plots ranged from 4.29 mg/L to 6 mg/L throughout the experimental period, while DO concentrations in hypoxia chambers steadily decreased (Fig. 2A). Background-dissolved oxygen levels during the experimental period at our study site were considered well above conventional thresholds of hypoxia (2.8 mg/L), although equilibrium concentrations of dissolved oxygen were slightly lower than a saturation concentration of 6.2 mg/L (44). In the chamber associated with MiniDOT logger 3, DO concentrations decreased drastically starting at hour 5 and reached levels <0.1 mg/L at hour 15 of the experiment (Fig. 2A). At hour 15, hypoxia chamber plot 1 was at 2.46 mg/L DO and hypoxia chamber plot 4 was at 3.08 mg/L DO. Our open-chamber plots at the same time of incubation ranged from 5.5 to 6.0 mg/L DO. The oxygen concentrations in hypoxia chamber plots 1 and 4 continued to decline thereafter. We observed *in situ* that corals within chamber 3 experienced severe bleaching. Over the course of 48 hours, water temperature ranged from 29.42°C to 30.08°C in the Punta Caracol experimental plots (Fig. S1).

### Microbial community characterization

Microbial communities were characterized for a total of 56 coral mucus samples from *Agaricia lamarcki* and *Siderastrea siderea* collected from three different sites in May 2019 (Fig. 1; Table S1). After quality filtering and joining, an average of 56,660 sequencing reads (11,273–116,996) per coral sample was used in the analysis (Table S1). A total of 157 archaeal ASVs and 22,666 bacterial ASVs were detected. After filtering ASVs with a mean read count of less than 5, a total of 2 archaeal ASVs and 877 bacterial ASVs were detected. One control sample from the extraction kit was also sequenced, and after quality filtering and joining, it had 22,860 reads, which were classified as 78 bacterial ASVs (Table S2). Sequencing reads with primers and adapters removed are available at NCBI’s Sequence Read Archive under BioProject PRJNA641080.

**Fig 3:**
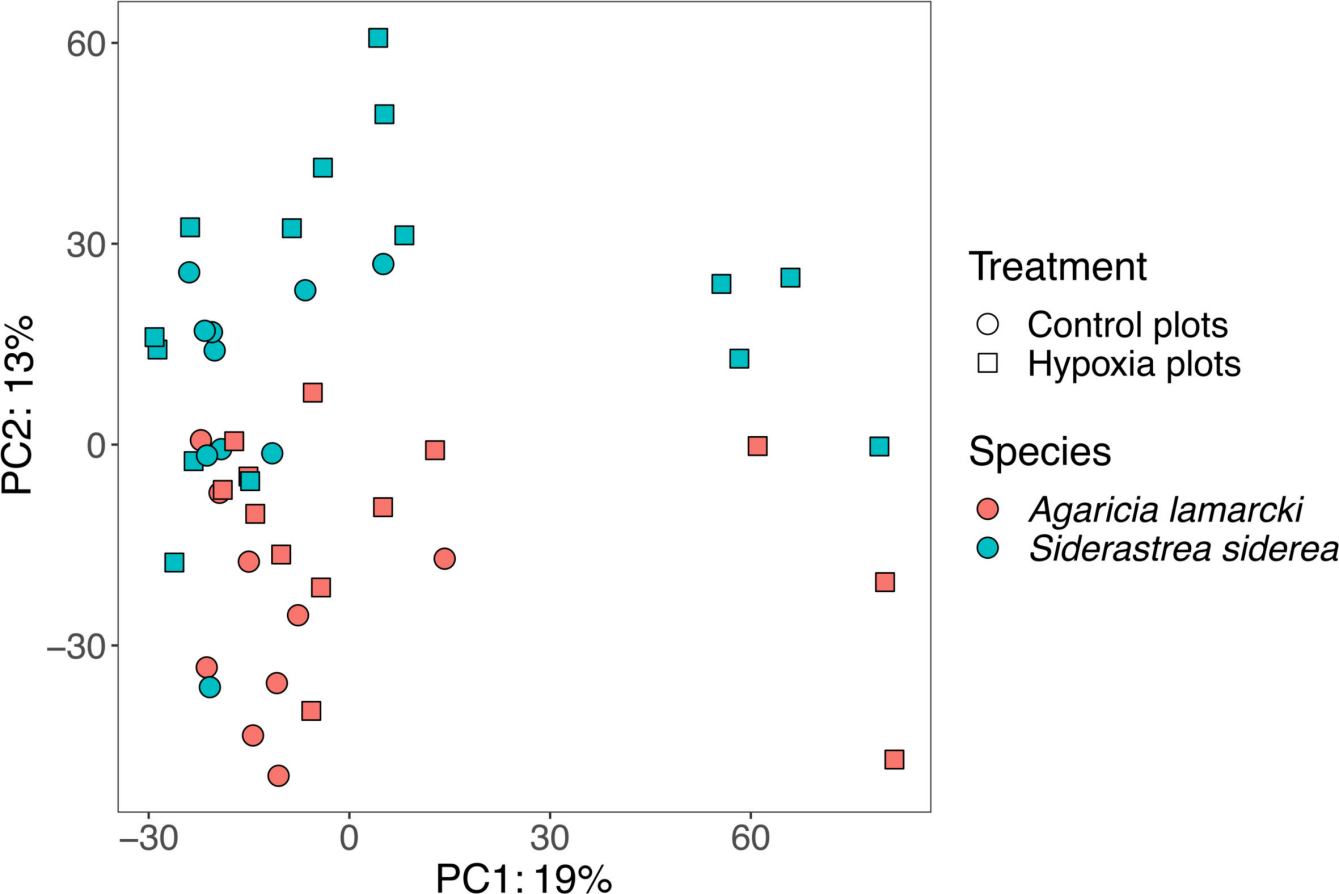
Principal component analysis of microbial community structure in corals in the control plots and corals in the hypoxia plots.

Overall, the microbial community structure in the experimental plots differed by coral species, although the effect size was small (PERMANOVA, *P* = 0.001, *R* ^2^ = 0.08; Fig. 3). Additionally, the microbial community structure differed among corals in the control plots and the hypoxia plots, although the effect size was small (PERMANOVA, *P* = 0.001, *R* ^2^ = 0.06; Fig. 3). The interaction between coral species and treatment was not significant (PERMANOVA, *P* > 0.05, *R* ^2^ = 0.02). Additionally, there was no significant difference in coral microbial community structure between the unmanipulated *S. siderea* sampled in Tierra Oscura (*n* = 3), which had previously experienced hypoxia, and Finca (*n* = 3), which had no documented hypoxia (ANOSIM, *P* > 0.05, *R* ^2^ = 0.63).

**Fig 4:**
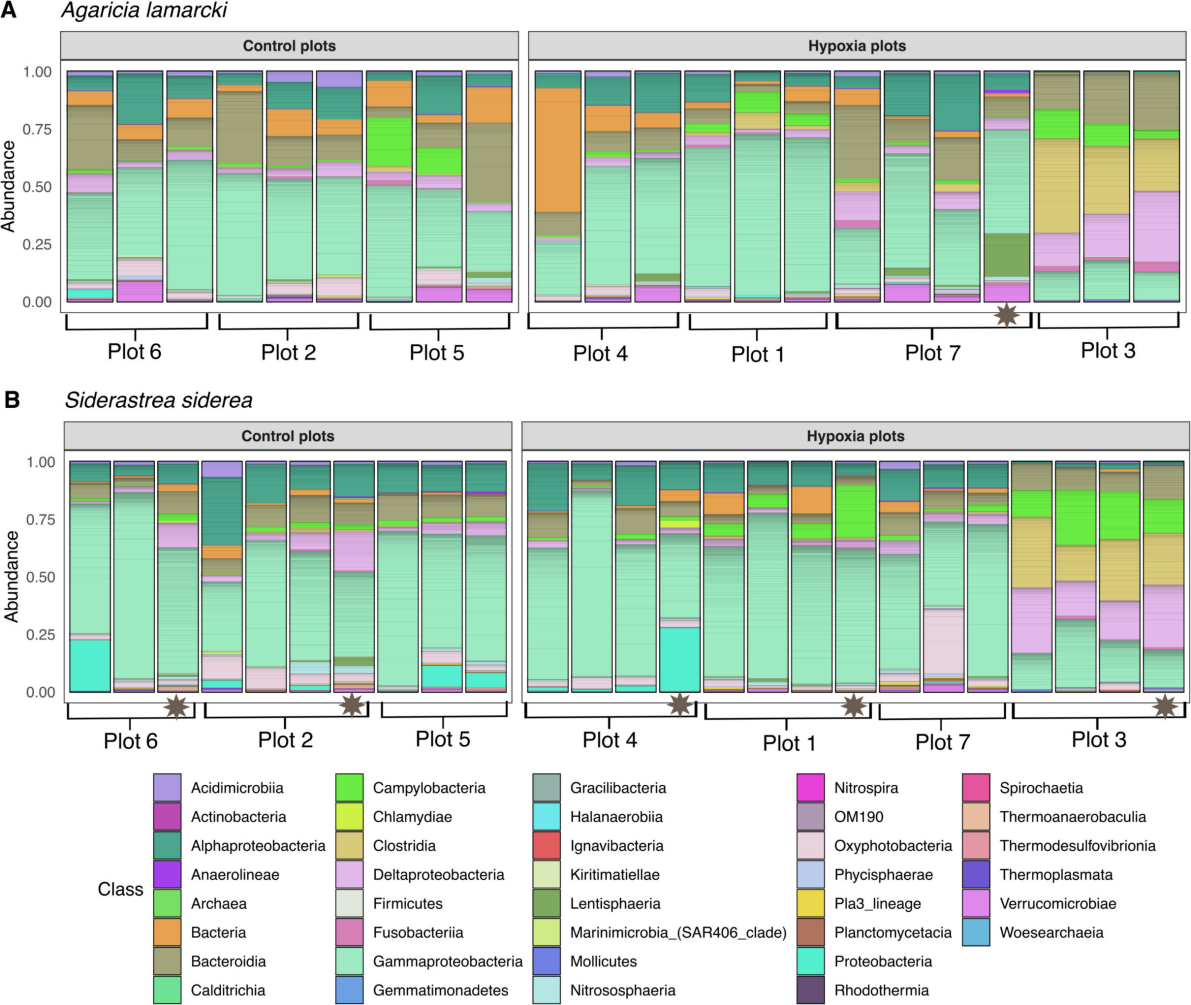
Relative abundance of amplicon sequence variants, colored by class, in corals in the control plots and corals in the hypoxia plots for *Agaricia lamarcki* (**A**) and *Siderastrea siderea* (**B**). Gray stars indicate local Punta Caracol coral colonies in the incubation chambers.

Bacterial taxa belonging to the *Alphaproteobacteria*, *Gammaproteobacteria*, and *Bacteroidia* were commonly detected in all samples, regardless of treatment and species (Fig. 4), consistent with previous studies of coral microbiomes (58). All ASVs classified only as “Bacteria” (*n* = 22) were searched with BLASTn, and sequences labeled as mitochondria by NCBI were removed from the data set. Of the 22 ASVs classified only as “Bacteria,” only one matched mitochondrial sequences (0.11% of ASVs). The most abundant ASV classified only as Bacteria in both species (Fig. 4) was 87% similar to an uncultivated bacterial sequence associated with the cold-water coral *Lophelia pertusa* sampled in Norway (GenBank Accession AM911366) (59) based on BLASTn searches. Additionally, the most abundant ASV classified only to class *Gammaproteobacteria* was 98% similar to a clone library sequence from an uncultivated Caribbean coral-associated bacterium (GenBank Accession KU243233) (60). The most abundant ASV classified only to phyla *Proteobacteria* in *S. siderea* (Fig. 4B) was 92% similar to a clone library sequence from an uncultivated *Deltaproteobacteria* associated with the coral *Pavona cactus* originating from the Red Sea (GenBank Accession EU847601) (61). Overall, there were no apparent patterns or differences in alpha diversity between the treatment types.

**Fig 5:**
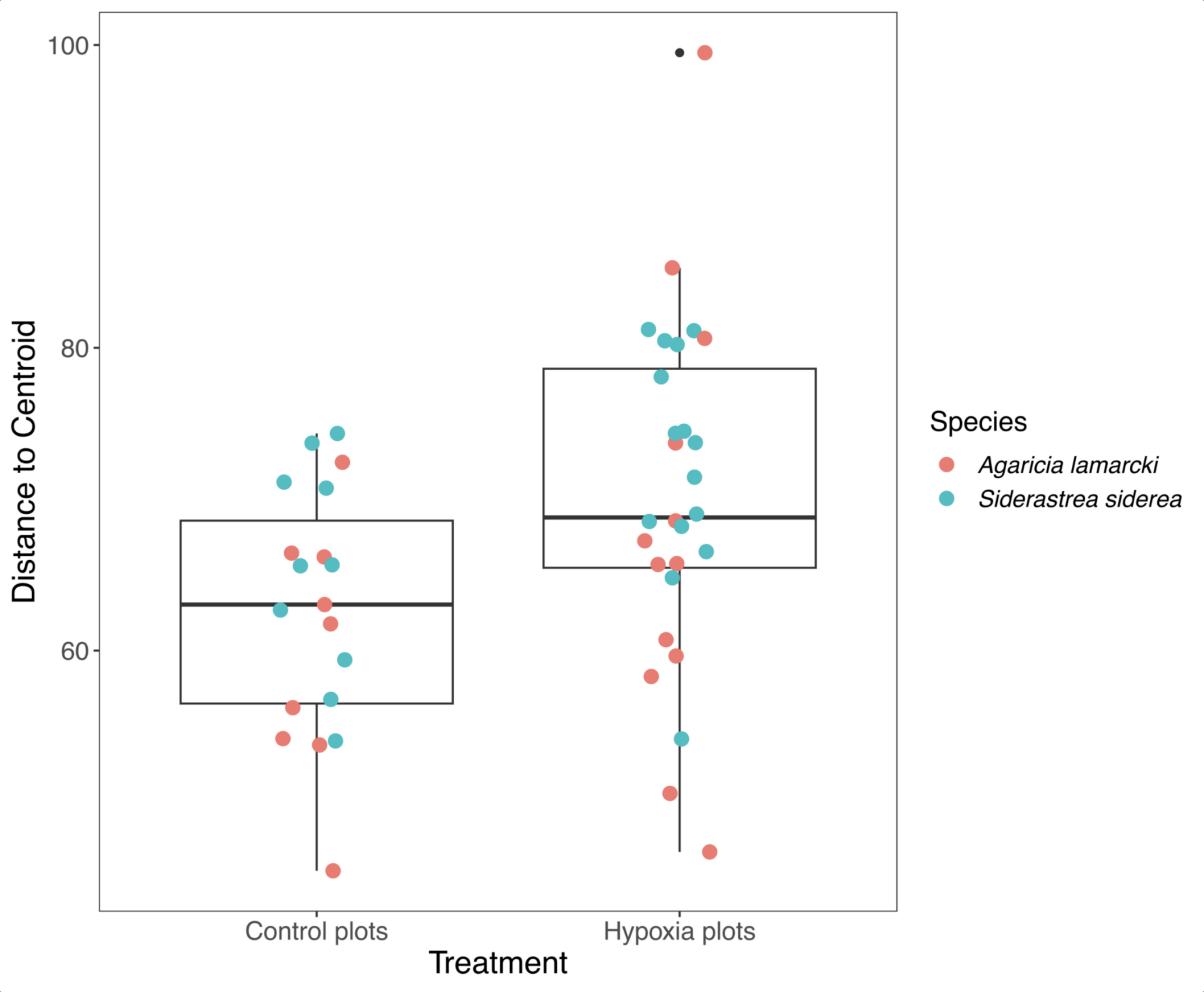
The dispersion of beta diversity shown as the distance to centroid in microbial communities from *S. siderea* and *A. lamarcki* colonies in control, normoxic plots, and hypoxic plots.

Because stress often has a stochastic effect on microbial community composition (62), we examined the dispersion of beta diversity according to treatment type (Fig. 5). In both *A. lamarcki* and *S. siderea*, hypoxia had a clear stochastic effect on microbiome composition, as affected colonies had higher variation in their microbiomes. In colonies that only experienced normoxia, microbial community composition had lower variability (Fig. 5). Analysis of variance of the linear model showed that beta diversity dispersion was significantly different between the hypoxic and control treatments (ANOVA, *P* = 0.02), but not for coral species (ANOVA, *P* = 0.09) or the combined factors (ANOVA, *P* = 0.88).

**Fig 6:**
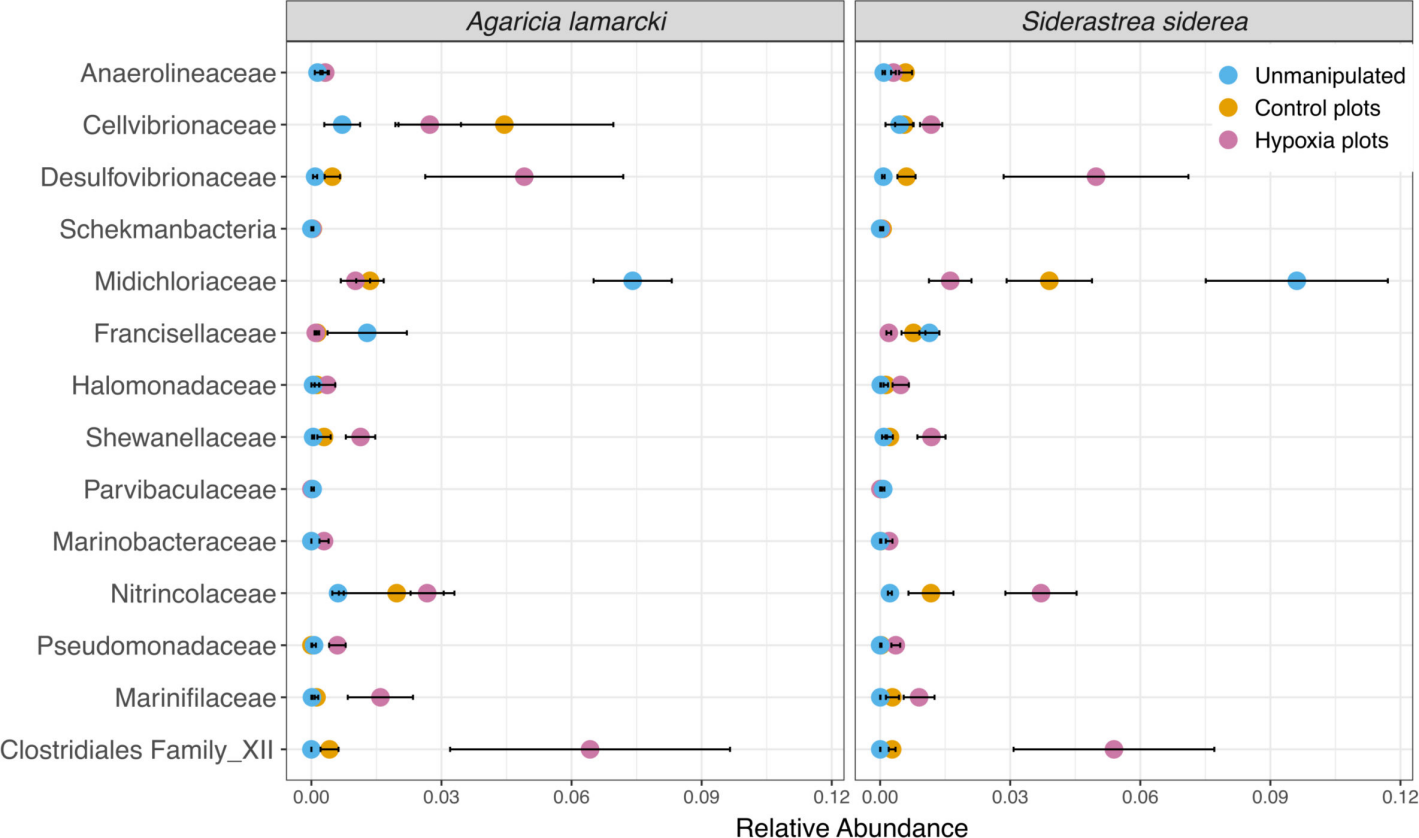
Mean relative abundance of 14 microbial families that were differentially abundant across treatment types: unmanipulated corals from Finca and Tierra Oscura, corals in the control plots, and corals in the hypoxic plots. Points represent the average relative abundance and error bars depict the standard error from analysis of all 56 coral samples. *Desulfovibrionaceae* and *Clostridiales* Family XII were each a magnitude more abundant in hypoxic plots than in control, oxygenated plots in both species.

Differences among treatments in the microbial community structure were primarily driven by 14 differentially abundant families (Fig. 6). These families were detected in at least 70% of the samples and were significantly different (ANOVA, *P* = 0.05) among unmanipulated corals from Tierra Oscura and Finca, control plots, and hypoxic plots (Fig. 6). The largest differences among treatment types were observed in families *Desulfovibrionaceae*, *Nitrincolaceae*, *Clostridiales* Family XII, and *Midichloriaceae*. The relative abundances of *Desulfovibrionaceae*, *Nitrincolaceae*, and *Clostridiales* Family XII were higher in the hypoxia treatment, whereas family *Midichloriaceae* was highest in the unmanipulated corals (Fig. 6). *Clostridiales* Family XII was more abundant in corals exposed to hypoxia and less abundant in unmanipulated and control plot corals.

Hypoxic chamber 3 experienced a sudden and dramatic drop in dissolved oxygen concentrations 5 hours following initiation of the incubation period and was completely hypoxic for 36 hours (Fig. 2A). This was associated with the largest magnitude response of the microbiome relative to the other plots. The seven microbial communities grouped on the right side of the PCA (Fig. 3) were from corals exposed to extremely low dissolved oxygen concentrations in chamber 3 (Fig. 2A) that ultimately bleached. The corals in this chamber included three colonies of *A. lamarcki* and four colonies of *S. siderea*, one of which was a local Punta Caracol *S. siderea* colony. Microbial community structure varied more by chamber (PERMANOVA, *P* = 0.001, *R* ^2^ = 0.37) than by either species or treatment. Increases in the typically anaerobic classes *Clostridia*, *Deltaproteobacteria*, and *Campylobacteria* were detected in both coral species in hypoxic chamber 3 (Fig. 4) and this trend was further explored.

**Fig 7:**
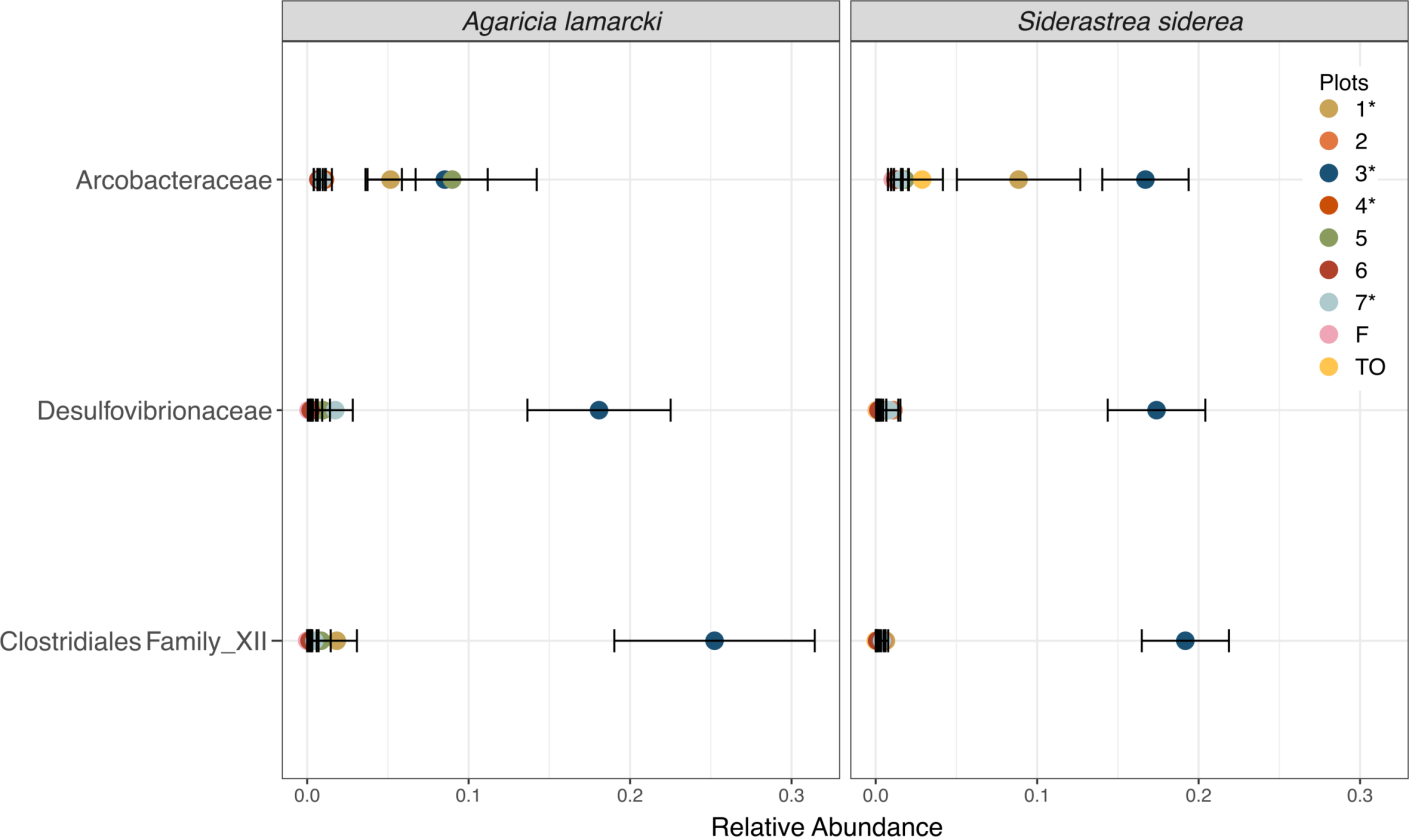
Mean relative abundance of 3 families that were differentially abundant across chambers and in corals sampled in Finca (**F**) and Tierra Oscura (TO). Colored points represent the average relative abundance of the families in each plot, and error bars depict the standard error from analysis of 56 coral samples. Asterisks next to plot numbers represent hypoxic plots. Families *Arcobacteraceae*, *Clostridiales Family XII*, and *Desulfovibrionaceae* increased significantly in corals that experienced hypoxia for the longest (36 hours). *Arcobacteraceae* was specifically highest in *S. siderea* colonies that experienced hypoxia for the longest.

Differences among the plots were primarily driven by 40 differentially abundant bacterial families. Those that were detected in higher abundances in both coral species from chamber 3, the plot with the most prolonged hypoxia, include *Arcobacteraceae*, *Prolixibacteraceae*, *Marinilabiliaceae*, *Desulfobacteraceae*, *Bacteroidales*, *Peptostreptococcaceae*, *Desulfovibrionaceae*, *Marinifilaceae*, and *Clostridiales* Family XII (Fig. S2). The relative abundances of *Midichloriaceae* were lowest in chamber 3, as were unclassified families of *Gammaproteobacteria* and *Proteobacteria* families (Fig. S2). Families *Colwelliaceae* and *Vibrionaceae* were detected in higher abundances in hypoxic chamber 1. Both coral species harbored several families in common that had similar responses to hypoxia, including *Arcobacteraceae*, *Desulfovibrionaceae*, and *Clostridiales* Family XII (Fig. 7). In *A. lamarcki* from hypoxic plot 3, families *Desulfovibrionaceae* and *Clostridiales* Family XII comprised an average of 18% and 25% of the microbiomes, respectively. In all other plots, *Desulfovibrionaceae* and *Clostridiales* Family XII comprised <2% of the microbiome from *A. lamarcki* (Fig. 7). These patterns are also reflected in *S. siderea* from hypoxic plot 3*,* in which families *Desulfovibrionaceae* and *Clostridiales* Family XII comprised an average of 17% and 19%, respectively. In all other plots, *Desulfovibrionaceae* and *Clostridiales* Family XII comprised <2% of the microbiome from *S. siderea* (Fig. 7). In *S. siderea*, family *Arcobacteraceae* comprised an average of 17% of the microbiome from hypoxic plot 3, 9% of the microbiome from hypoxic plot 1, and <2% of the microbiome from all other plots (Fig. 7).

To determine if differentially abundant families were driven by particular ASVs, an indicator species analysis was performed on all samples. Of the 878 ASVs tested, 144 ASVs were considered indicator species for hypoxia, but only four ASVs had a correlation statistic ≥0.50 (Fig. S3). These include an *Alteromonas* ASV, a *Neptuniibacter* ASV, an *Aestuariicella* ASV, and a *Marinobacter* ASV (Table S3). We detected no indicator ASVs common to both *A. lamarcki* and *S. siderea* when exposed to hypoxic stress. Therefore, although the two coral species exhibited similar shifts in differentially abundant microbial families, these patterns were not driven by individual taxa (ASVs) common to both *A. lamarcki* and *S. siderea*.

## DISCUSSION

We observed a shift in the microbial communities of corals *A. lamarcki* and *S. siderea* following just 48 hours of experimental deoxygenation. Though the overall microbiome shift was stochastic in response to hypoxic stress, certain bacterial groups responded deterministically in both coral species. In response to hypoxia, we saw increased variability of the coral microbial community composition, regardless of species. Hypoxic conditions resulted in an increase of anaerobic and potentially pathogenic bacteria in the classes *Deltaproteobacteria*, *Campylobacteria*, and *Clostridia* in the microbiome of both *A. lamarcki* and *S. siderea*. This is most apparent in corals that experienced the most severe hypoxia associated with plot 3. Moreover, both coral species exhibited changes of similar magnitude in the relative abundances of many families, most notably *Arcobacteraceae*, *Desulfovibrionaceae*, *Clostridiales* Family XII, *Nitrincolaceae*, and *Midichloriaceae*. Although we detected statistically significant differences in microbial communities between oxygen treatments for both species, the effect size of that difference was relatively small.

Prior studies performed have shown primarily stochastic shifts in the microbiome in response to other environmental pressures and have corroborated our results. For example, stressors including nutrient pollution, overfishing, and thermal stress on reefs were correlated with an increase in the dispersion of beta diversity dispersion in the coral microbiome (62). Because of this, the combination of deterministic and stochastic outcomes from our study may suggest some host regulation of the microbiome in response to hypoxic stress. These corals may have curated the members of their microbial community to better deal with the stress of deoxygenation (63). However, increases in beta diversity and destabilization of the microbiome have also been associated with host tissue loss (62), disease (34, 36), and mortality (34, 36, 62, 64). Because many taxa in our study are often associated with coral stress, it is likely that opportunistic taxa are being enriched in the microbiome under hypoxic conditions. Examining the functional role of these members may explain some uniformities of the microbiome across both coral species in response to hypoxia.

### Functional significance of microbiome shifts

Under experimentally induced hypoxia, we documented an increase in *Deltaproteobacteria*, specifically the family *Desulfovibrionaceae. Deltaproteobacteria* are known for their role as sulfate-reducing microorganisms (SRM) (65, 66). In marine ecosystems, *Deltaproteobacteria* are mainly found in sediment, where they are the predominant SRMs in terms of abundance and activity (67). *Desulfovibrionaceae*, a well-known family within *Deltaproteobacteria*, includes numerous sulfate-reducing species which produce hydrogen sulfide that can degrade coral health and result in disease (68, 69). Members of this family have been implicated in Black Band Disease as a producer of sulfide (68, 69). Further, *Desulfovibrionaceae* were detected in corals infected with stony coral tissue loss disease (SCTLD), and the genera *Desulfovibrio* and *Halodesulfovibrio* have been recently described as bioindicators of the disease (70, 71). *Deltaproteobacteria* in the coral microbiome are likely producing sulfide and playing an antagonistic role and may contribute to increased coral disease prevalence associated with reef hypoxia, but the definitive role of this class in the coral microbiome remains to be confirmed, particularly under environmental stressors like hypoxia.

We also documented an increase in the class *Campylobacteria* during experimental deoxygenation in the coral microbiome. Microbes within this taxonomic group, and many species of *Epsilonbacterota* in particular, play important roles in carbon, nitrogen, and sulfur cycling, especially in symbiosis with their host (72, 73). *Epsilonbacterota* thrive in anaerobic or microaerobic environments rich with sulfur (72), including hydrothermal vents (73) and sediments associated with seagrass roots (74). On corals experiencing hypoxia, members of *Campylobacteria* may alleviate stress by oxidizing some of the toxic sulfides produced by microbial respiration including *Deltaproteobacteria* in the holobiont. The increase in sulfur-oxidizing *Campylobacteria* during hypoxia may therefore be a form of rapid adaptation to this stressor, conferring resilience to deoxygenation stress for corals. For instance, family *Arcobacteraceae*, which were enriched under the most extreme low-oxygen conditions here, are known for the sulfide-oxidizing capabilities (75, 76), producing both sulfate and filamentous sulfur (76), and may help detoxify the surrounding sulfidic microenvironment around corals. *Arcobacteraceae* are associated with changes in the coral holobiont under stress conditions, growing rapidly in the microbiome in thermally stressed corals (77) and corals living in polluted waters (78). Though members of this group have also been associated with coral diseases, such as white syndrome (79), brown band disease (79), white plague disease (80), and stony coral tissue loss disease (71), the role of *Arcobacteraceae* during hypoxic stress in the coral holobiont remains unknown.

*Clostridia*, including *Clostridiales* Family XII, also increased in abundance on both species of coral host in response to deoxygenation. This change was especially prominent in chamber 3, where hypoxia was most severe and sustained. *Clostridia* is a large polyphyletic class of obligate and facultative anaerobes known for producing the highest number of toxins of any bacterial group and causing severe disease in humans and animals (81). However, the role of *Clostridia* in coral remains ambiguous. Most Gram-positive sulfate-reducing bacteria belong to the class *Clostridia*, so these taxa may play a similar role to the *Deltaproteobacteria* in the coral holobiont (82). Further, corals that harbor higher abundances of *Clostridia* ASVs are more often associated with disease (83). For example, *Clostridiales* ASVs are enriched in the surface mucus layer and tissue near stony coral tissue loss disease (SCTLD) lesions (71, 84, 85) and Black Band Disease mats (86, 87). An increase of *Clostridia* has also been documented in the microbiome when corals are exposed to thermal stress (88). Generally, higher abundances of *Clostridia* in the coral microbiome are often associated with host stress. In our study, members of *Clostridia* are likely playing an antagonistic role in the coral holobiont as sulfide producers (82) or as opportunistic pathogens as oxygen levels decline (83). However, *Clostridia* remains unsubstantiated as the causative agent of any coral disease, and it may simply respond opportunistically to stress-associated changes in the holobiont.

Family *Nitrincolaceae*, belonging to class *Gammaproteobacteria*, was more abundant in corals exposed to hypoxia. This increase in *Nitrincolaceae* is consistent with observations in the microbial community in the water column above a reef during the 2017 hypoxic event in Bahiá Almirante when *Nitrincolaceae* was found only in hypoxic water samples from that event, and not in oxygenated water samples at that site following the event or at a reference site (40). Species within this family have genes for nitrite reductase, nitric oxide reductase, and nitrous oxide reductase (89, 90). As such, members of *Nitrincolaceae* have the potential to produce nitrate (NO_3_), nitrous oxide (N_2_O), and dinitrogen (N_2_). The denitrification of bioavailable nitrogen to nitrogen gas in low-oxygen systems may aid in mitigating the eutrophication that usually precedes and occurs with hypoxia (31). Taxa within this family have also been described as following short-term “feast and famine” dynamics of nutrient uptake and are aggressive heterotrophs (90). During seasonal transitions in the Southern Ocean, *Nitrincolaceae* rapidly take up nutrients from phytoplankton-derived organic matter and iron (90). In hypoxic conditions on coral reefs, it is possible that our observed increase in *Nitrincolaceae* signified their role as opportunistic heterotrophs. Their increase in the holobiont may be due to coral tissue decay, as death of both coral and associated *Symbiodiniaceae* may supply the bacteria with the organic matter and iron they need to thrive in this environment. Their increase may also be an opportunistic response to degrading host health, as some taxa within *Nitrincolaceae* are considered bioindicators for stony coral tissue loss disease in *S. siderea* (70).

Family *Midichloriaceae* (order *Rickettsiales*) decreased in all corals associated with hypoxic conditions, including those in chamber 3. *Rickettsiales* are obligate intracellular bacteria of eukaryotes and include well-known zoonotic pathogens (91). Though previously implicated in white band disease (92, 93), many recent studies have detected the *Rickettsiales* genus MD3-55 (*Candidatus* Aquarickettsia rowherii) as an abundant member of the apparently healthy *Acropora cervicornis* microbiome in the Cayman Islands (94), the Florida Keys (95 – 97), and Panama (98, 99). *Rickettsiales* have previously been found in low abundances on six healthy coral species sampled in the Bocas del Toro region of Panama (99). In our study, family *Midichloriaceae* were detected at lower relative abundances under hypoxic conditions. This may be due to some tissue loss in corals that experienced severe hypoxia in chamber 3 and indicate that *Rickettsiales* has a dependence or preference for healthy corals. Though their role in the coral microbiome remains unclear, our study provides further evidence that *Rickettsiales* is a constituent of healthy holobiont that declines in abundance with stress.

### Holobiont response to hypoxic stress

Differences in hypoxia tolerance thresholds among coral species may be due to the regime of hypoxia exposure, host stress responses, or microbial function. Environmental history can also affect the survival of coral during subsequent exposures to low oxygen (100). Previous work has demonstrated that coral species vary in their susceptibility to hypoxia (6, 101 – 104). For example, *A. cervicornis* suffered tissue loss and mortality within a day of exposure to hypoxia in lab experiments, whereas *Orbicella faveolata* was unaffected after 11 days of continuous hypoxia exposure (101). *Stephanocoenia intersepta* from Bahiá Almirante exhibited a threefold greater hypoxia tolerance than *A. lamarcki* in lab-based experiments (6). Further, following a deoxygenation event in Morrocoy National Park, Venezuela, *Acropora* and some *Montastrea* colonies exhibited bleaching, while *S. siderea*, *Porites astreoides*, and *P. porites* did not suffer any damage (102). These data follow a trend: plating and branching corals typically have a higher mortality rate than massive and encrusting corals under hypoxic conditions (23, 28, 100, 102, 103). These differences in hypoxia tolerance have been observed in prior studies done in Bahiá Almirante, which record *Agaricia* species as hypoxia sensitive (6, 40) and *S. siderea* as hypoxia resilient (40).

In addition to innate resilience that appears to vary with morphology, transcriptomic analysis has revealed that corals possess a complete and active hypoxia-inducible factor (HIF)-mediated hypoxia response system (HRS) that confers some hypoxia resilience (104). The effectiveness of this hypoxia response system can differ between coral species. For example, *Acropora tenuis* was more resistant to hypoxic stress when compared to *Acropora selago. A. tenuis* exhibited bleaching resistance and showed a strong inducibility of HIF genes in response to hypoxic stress. In contrast, *A. selago* exhibited a bleaching phenotypic response and was accompanied by lower gene expression of the hypoxia-inducible factor (HIF)-mediated hypoxia response system (104). Therefore, differences in coral response to hypoxia are in part due to the effectiveness of their HIF-HRSs.

Though historic exposure and the HIF-HRS each contribute to host survival, it is likely a synergistic effect between historic exposure, the HIF-HRS, and the coral microbiome that confer the most resilience to the holobiont during hypoxia. Past research has demonstrated that corals may shuffle members of their holobiont to bring about the selection of a more advantageous microbiome in response to environmental stressors (35, 105, 106). This microbial shuffling may act as a form of rapid adaptation to changing environmental conditions rather than mutation and natural selection (63). In our results, we observed a rapid shift in the community composition of the microbiome in response to hypoxia associated with the survival of corals through a period of intense deoxygenation stress. We presume that some microbial taxa that increased in abundance with hypoxia may play a role in host survival and resilience by eliminating toxic natural products around the microenvironment of the coral or by filling some metabolic needs during stress. This appears to be a common overall strategy across coral species that has developed in response to the selective pressure of hypoxia given that we observed it across two species that are distantly related taxonomically and are at opposite ends of the spectrum with regard to hypoxia tolerance. However, the exact ASV constituents that contributed to the shifts at the family level differed between the corals, suggesting different co-evolutionary pathways which may contribute to the difference in hypoxia tolerance of the coral hosts.

### Conclusions

Marine deoxygenation will worsen with continued climate change, and with its potential to degrade coral reefs, it is essential to understand patterns of resilience revealed in the microbiome. Given the results of this study, we suspect that increased abundances in some microbial taxa with hypoxia may play a role in host resilience by detoxifying the microenvironment around the coral host, such as *Campylobacteria* (*Arcobacteraceae*). Other taxa, such as *Midichloriaceae* and *Clostridiales* Family XII, have more ambiguous roles in the coral microbiome, though their shifts in response to hypoxia warrant further investigation. Alternatively, the increases in these groups may indicate a shift in the coral microbial community towards opportunists exploiting host stress. We hypothesize that enhancement of these anaerobes, facultative anaerobes, or microaerophiles in the microbiome fill necessary and diverse metabolic niches in the holobiont during hypoxic stress while simultaneously indicating deoxygenation. Future studies that examine the functional roles of the coral microbiome through metagenomic or metatranscriptomic analyses can further advance our understanding by testing these hypotheses regarding how the microbiome can mitigate the degradation of coral reefs under hypoxic conditions.

## Data Availability

Sequences are available on the NCBI Sequence Read Archive (SRA) BioProject PRJNA641080 under accession numbers SAMN15298019-SAMN15298075.

## SUPPLEMENTAL MATERIAL

Supplemental file 1 aem.00577-23-s0001.pdf Supplemental figures and tables

ASM does not own the copyrights to Supplemental Material that may be linked to, or accessed through, an article. The authors have granted ASM a non-exclusive, world-wide license to publish the Supplemental Material files. Please contact the corresponding author directly for reuse.
