# Supplementary material for: Shifts in the coral microbiome in response to in situ experimental deoxygenation
Source: Appl Environ Microbiol. 2023 Nov 2;89(11):e00577-23. doi: 10.1128/aem.00577-23 (PMC10686059; doi:10.1128/aem.00577-23)
Supplement: Supplemental file 1 — Supplemental figures and tables [file aem.00577-23-s0001.pdf]

Table S1: Sample metadata for microbial communities collected from resident corals, corals in the control plots, and corals in the hypoxia plots. Reads with adapters removed are deposited in NCBI under Bioproject Accession # PRJNA641080.

**Sample**	**Species**	**Site**	**Treatment**	**Logger MiniDOT**	**Origin**	**Raw Reads**	**Quality-filtered Reads**	**Genbank Accession Number**
AA-1	*Siderastrea siderea*	Tierra Oscura	Unmanipulated	7	local	49259	42286	SAMN15298072
AA-10	*Agaricia lamarcki*	Punta Caracol	Control plots	8	transplant	72996	44527	SAMN15298025
AA-11	*Agaricia lamarcki*	Punta Caracol	Control plots	8	transplant	69136	51493	SAMN15298026
AA-12	*Agaricia lamarcki*	Punta Caracol	Control plots	8	transplant	55873	39565	SAMN15298027
AA-13	*Siderastrea siderea*	Punta Caracol	Control plots	8	transplant	72852	59879	SAMN15298028
AA-15	*Siderastrea siderea*	Punta Caracol	Control plots	8	transplant	124387	95963	SAMN15298029
AA-16	*Siderastrea siderea*	Punta Caracol	Control plots	8	local	72922	49242	SAMN15298030
AA-17	*Agaricia lamarcki*	Punta Caracol	Hypoxia plots	4	transplant	48825	32888	SAMN15298031
AA-18	*Agaricia lamarcki*	Punta Caracol	Hypoxia plots	4	transplant	23792	14001	SAMN15298032
AA-19	*Agaricia lamarcki*	Punta Caracol	Hypoxia plots	4	transplant	52732	36829	SAMN15298033
AA-2	*Siderastrea siderea*	Tierra Oscura	Unmanipulated	7	untreated	54452	43803	SAMN15298073
AA-20	*Siderastrea siderea*	Punta Caracol	Hypoxia plots	4	transplant	82902	63797	SAMN15298034

Sample	Species	Site	Treatment	Logger MiniDOT	Origin	Raw Reads	Quality-filtered Reads	Genbank Accession Number
AA-21	*Siderastrea siderea*	Punta Caracol	Hypoxia plots	4	transplant	60194	54633	SAMN15298035
AA-22	*Siderastrea siderea*	Punta Caracol	Hypoxia plots	4	transplant	95296	72904	SAMN15298036
AA-23	*Siderastrea siderea*	Punta Caracol	Hypoxia plots	4	local	109034	85811	SAMN15298037
AA-24	*Agaricia lamarcki*	Punta Caracol	Control plots	2	transplant	99942	78780	SAMN15298038
AA-25	*Agaricia lamarcki*	Punta Caracol	Control plots	2	transplant	55382	41772	SAMN15298039
AA-26	*Agaricia lamarcki*	Punta Caracol	Control plots	2	transplant	43425	27726	SAMN15298040
AA-27	*Siderastrea siderea*	Punta Caracol	Control plots	2	transplant	115867	101459	SAMN15298041
AA-28	*Siderastrea siderea*	Punta Caracol	Control plots	2	transplant	57511	48616	SAMN15298042
AA-29	*Siderastrea siderea*	Punta Caracol	Control plots	2	transplant	98000	74892	SAMN15298043
AA-3	*Siderastrea siderea*	Tierra Oscura	Unmanipulated	7	untreated	62139	27172	SAMN15298074
AA-30	*Siderastrea siderea*	Punta Caracol	Control plots	2	local	94755	63755	SAMN15298044
AA-31	*Agaricia lamarcki*	Punta Caracol	Hypoxia plots	1	transplant	26776	20705	SAMN15298045
AA-32	*Agaricia lamarcki*	Punta Caracol	Hypoxia plots	1	transplant	67885	51075	SAMN15298046
AA-33	*Agaricia lamarcki*	Punta Caracol	Hypoxia plots	1	transplant	58167	38100	SAMN15298047

Sample	Species	Site	Treatment	Logger MiniDOT	Origin	Raw Reads	Quality-filtered Reads	Genbank Accession Number
AA-34	*Siderastrea siderea*	Punta Caracol	Hypoxia plots	1	transplant	94219	70797	SAMN15298048
AA-35	*Siderastrea siderea*	Punta Caracol	Hypoxia plots	1	transplant	143046	102323	SAMN15298049
AA-36	*Siderastrea siderea*	Punta Caracol	Hypoxia plots	1	transplant	150572	116341	SAMN15298050
AA-37	*Siderastrea siderea*	Punta Caracol	Hypoxia plots	1	local	87073	64928	SAMN15298051
AA-38	*Agaricia lamarcki*	Punta Caracol	Control plots	5	transplant	38507	24081	SAMN15298052
AA-39	*Agaricia lamarcki*	Punta Caracol	Control plots	5	transplant	33881	20531	SAMN15298053
AA-4	*Siderastrea siderea*	Finca	Unmanipulated	6	untreated	64689	53031	SAMN15298019
AA-40	*Agaricia lamarcki*	Punta Caracol	Control plots	5	transplant	38022	20523	SAMN15298054
AA-41	*Siderastrea siderea*	Punta Caracol	Control plots	5	transplant	112253	77337	SAMN15298055
AA-42	*Siderastrea siderea*	Punta Caracol	Control plots	5	transplant	75539	56500	SAMN15298056
AA-43	*Siderastrea siderea*	Punta Caracol	Control plots	5	transplant	96692	69864	SAMN15298057
AA-45	*Agaricia lamarcki*	Punta Caracol	Hypoxia plots	9	transplant	42692	32250	SAMN15298058
AA-46	*Agaricia lamarcki*	Punta Caracol	Hypoxia plots	9	transplant	67374	44913	SAMN15298059
AA-47	*Agaricia lamarcki*	Punta Caracol	Hypoxia plots	9	transplant	56490	37120	SAMN15298060

**Sample**	**Species**	**Site**	**Treatment**	**Logger MiniDOT**	**Origin**	**Raw Reads**	**Quality-filtered Reads**	**Genbank Accession Number**
AA-48	*Siderastrea siderea*	Punta Caracol	Hypoxia plots	9	transplant	72691	55489	SAMN15298061
AA-49	*Siderastrea siderea*	Punta Caracol	Hypoxia plots	9	transplant	108976	81950	SAMN15298062
AA-5	*Agaricia lamarcki*	Finca	Unmanipulated	6	untreated	25278	15468	SAMN15298020
AA-50	*Siderastrea siderea*	Punta Caracol	Hypoxia plots	9	transplant	94783	73627	SAMN15298063
AA-51	*Agaricia lamarcki*	Punta Caracol	Hypoxia plots	9	local	54180	34588	SAMN15298064
AA-52	*Agaricia lamarcki*	Punta Caracol	Hypoxia plots	3	transplant	86355	65649	SAMN15298065
AA-53	*Agaricia lamarcki*	Punta Caracol	Hypoxia plots	3	transplant	135465	101822	SAMN15298066
AA-54	*Agaricia lamarcki*	Punta Caracol	Hypoxia plots	3	transplant	116023	87007	SAMN15298067
AA-55	*Siderastrea siderea*	Punta Caracol	Hypoxia plots	3	transplant	145825	117000	SAMN15298068
AA-56	*Siderastrea siderea*	Punta Caracol	Hypoxia plots	3	transplant	87590	71748	SAMN15298069
AA-57	*Siderastrea siderea*	Punta Caracol	Hypoxia plots	3	transplant	104641	86314	SAMN15298070
AA-58	*Siderastrea siderea*	Punta Caracol	Hypoxia plots	3	local	139808	105161	SAMN15298071
AA-6	*Siderastrea siderea*	Finca	Unmanipulated	6	untreated	72892	55117	SAMN15298021
AA-7	*Agaricia lamarcki*	Finca	Unmanipulated	6	untreated	23408	11276	SAMN15298022

Sample	Species	Site	Treatment	Logger MiniDOT	Origin	Raw Reads	Quality-filtered Reads	Genbank Accession Number
AA-8	*Agaricia lamarcki*	Finca	Unmanipulated	6	untreated	50153	37907	SAMN15298023
AA-9	*Siderastrea siderea*	Finca	Unmanipulated	6	untreated	67689	54461	SAMN15298024
BLANK	extraction control	extraction control	extraction control	extraction control	extraction control	28286	22860	SAMN15298075

Table S2: Amplicon sequence variants detected in one control sample. Blank 1 was a no template control from DNA extraction kit through sequencing. The total counts of these ASVs in all coral samples is also given for comparison.

Kingdom	Phylum	Class	Order	Family	Genus	Blank Total (n=1)	Sample Total (n=56)	Amplicon Sequence Variants (ASVs)
Bacteria	Proteobacteria	Gammaproteobacteria	Vibrionales	Vibrionaceae	Vibrio	2	10	TACGGAGGGTGCGAGCGTTAATCGGAATTACT GGGCGTAAAGCGCATGCAGGTGGTTTGTTAAG TCAGATGTGAAAGCCCGGGGCTCAACCTCGGA AGGCCATTTGAAACTGGCAAACTAGAGTACTG TAGAGGGGGGTAGAATTTCAGGTGTAGCGGTG AAATGCGTAGAGATCTGAAGGAATACCAGTGG CGAAGGCGGCCCCCTGGACAGATACTGACACT CAGATGCGAAAGCGTGGGGAGCAAACAGG
Bacteria	Proteobacteria	Deltaproteobacteria	SAR324_clade (Marine_group_ B)	SAR324_clade (Marine_group _B)	SAR324 _clade(Marine_ group_B)	5	5906	TACGTAGGGTGCGAGCGTTGTTCGGAATTACT GGGCGTAAAGGGCGTGCAGGCGGATTGGCAAG CCGGAGGTGAAAGCCCGGGGCTCAACCCCGGA GGGTCTTTCGGAACTGCCAGTCTTGAGAGGGT CAGGGGCCAGCGGAATTCCTGGTGTAGAGGTG AAATTCGTAGAGATCAGGAGGAACACCGGCGG CGAAAGCGGCTGGCTGGGGCCACTCTGACGCT GAGGCGCGAAAGCGTGGGGAGCAAACAGG
Bacteria	Proteobacteria	Gammaproteobacteria	JTB23	JTB23	JTB23	5	13	TACGGAGGGTGCGAGCGTTAATCGGAATTACT GGGCGTAAAGCGCGCGTAGGTGGCTTGGTCAG TCGGATGTGAAAGCCCCGGGCTTAACCTGGGA ATTGCATTCGATACTGCCTGGCTAGAGTATGAT AGAGGAAAGTGGAATTCCCGGTGTAGCGGTGA AATGCGTAGATATCGGGAGGAACATCAGTGGC GAAGGCGACTTTCTGGATCAATACTGACACTG AGGTGCGAAAGCGTGGGTAGCAAACAGG
Bacteria	Planctomycetes	Phycisphaerae	Phycisphaerales	Phycisphaerac eae	Urania- 1B- 19_mari ne_sedi	7	3429	TACGAGAGGTCCGAGCGTTACGCGGAATTACT GGGCTTAAAGCGTACGTAGGCGGATCTACAGG TATCCTGTGAAAGCCCACGGCTCAACCGTGGA ATTGCAGGGTAAACCGTAGATCTTGAGGTGGC

Kingdom	Phylum	Class	Order	Family	Genus	Blank Total (n=1)	Sample Total (n=56)	Amplicon Sequence Variants (ASVs)
					ment_gr oup			TAGGGGCTCTCGGAACGGTAGGTGGAGTGGTG AAATGCGTTGATATCTACCGGAACGCCAAAGG GGAAGCCAGGGAGCTGGGGCCATTCTGACGCT GAGGTACGAAAGCGTGGGTAGCGAACGGG
Bacteria	Proteobacteria	Gammaproteobacteria	SAR86_clade	SAR86_clade	SAR86_ clade	7	353	TACGGAAGGTGCAAGCGTTAATCGGAATTACT GGGCGTAAAGCGCGCGTAGGTGGTTTGTTAAG TTGGATGTGAAAGCCCTGGGCTCAACCTAGGA ACTGCATCCAAAACTAACTCACTAGAGTACGA TAGAGGGAGGTAGAATTCATAGTGTAGCGGTG GAATGCGTAGATATTATGAAGAATACCAGTGG CGAAGGCGGCCTCCTGGATCTGTACTGACACT AAGGTGCGAAAGCGTGGGTAGCGAACAGG
Bacteria	Bacteroidetes	Bacteroidia	Cytophagales	Cyclobacteriac eae	Cycloba cteriacea e	7	8	TACGGAGGGTGCAAGCGTTGTCCGGATTTACT GGGTTTAAAGGGTGCGTAGGCGGTTCTTTAAG TCAGTGGTGAAATCCTCCAGCTTAACTGGAGA GGGTCCATTGATACTGGAGAACTTGAGTACAT TAGGGGTATGCGGAATTGATGATGTAGCGGTG AAATGCATAGATATCATCAAGAACACCGATAG CGAAGGCAGCATACTGGACTGTAACTGACGCT GATGCACGAAAGCGTGGGTATCGAACAGG
Bacteria	Bacteroidetes	Bacteroidia	Bacteroidales	Rikenellaceae	Rikenell aceae	7	0	TACGGAGGATCCAAGCGTTATCCGGATTTATTG GGTTTAAAGGGTGCGTAGGCGGTGGATTAAGT TAGTCGTTAAATCGAGGGGCTCAAACTCTCAA TGGCGATTGATACTGGTTCACTAGAGATTATGT GCAGCGGGCGGAATGAGTAGTGTAGCGGTGAA ATGCTTAGATATTACTCAGAACACCGATTGCG AAGGCAGCTCGCTAACATATATCTGACGCTCA GCCACGAAAGCGTGGGTATCAAACAGG
Bacteria	Actinobacteria	Actinobacteria	Corynebacteriale s	Corynebacteria ceae	Coryneb acterium _1	7	0	TACGTAGGGTGCGAGCGTTGTCCGGAATTACT GGGCGTAAAGAGCTCGTAGGTGGTTTGTCGCG TCGTTTGTGTAATACCGCAGCTTAACTGTGGGG TTGCAGGCGATACGGGCATAACTTGAGTGCTG TAGGGGAGACTGGAATTCCTGGTGTAGCGGTG

Kingdom	Phylum	Class	Order	Family	Genus	Blank Total (n=1)	Sample Total (n=56)	Amplicon Sequence Variants (ASVs)
								GAATGCGCAGATATCAGGAGGAACACCGATGGCGAAGGCAGGTCTCTGGGCAGTAACTGACGCTGAGGAGCGAAAGCATGGGGAGCGAACAGG
Bacteria	Proteobacteria	Gammaproteobacteria	Vibrionales	Vibrionaceae	Catenococcus	7	0	TACGGAGGGTGCAAGCGTTAATCGGAATTACTGGGCGTAAAGCGCGCGTAGGTGGTTTGTTAAGCGGAATGTGAAAGCTCGGGGCTCAACCTCGGAATTGCATTTGAAACTGGCAGACTAGAGTACTGTAGAGGGGGGTAGAATTTCAGGTGTAGCGGTGAAATGCGTAGAGATCTGAAGGAATACCGGTGGCGAAGGCGGCCCCCTGGACAGATACTGACACTCAGATGCGAAAGCGTGGGGAGCAAACAGG
Bacteria	Proteobacteria	Alphaproteobacteria	Puniceispirillales	SAR116_clade	Candidatus_Puniceispirillum	7	0	TACGAAGGGGGCGAGCGTTGTTCGGAATTACTGGGCGTAAAGGGCGCGTAGGCGGTCCTTTAAGTTAGGCGTGAAAGCCCCGGGCTCAACCTGGGAACTGCGCTTGATACTGGAGGACTAGAAAACGGAAGAGGGTAGTGGAATTCCCAGTGTAGAGGTGAAATTCGTAGATATTGGGAAGAACACCAGTGGCGAAAGCGGCTACCTGGTCCGATTTTGACGCTGAGGCGCGAAAGCGTGGGGAGCAAACAGG
Bacteria	Planctomycetes	BD7-11	BD7-11	BD7-11	BD7-11	7	0	TACGTATGTGGCTAGCGTTGTTCGGAATCACTGGGCATAAAGAGTTCGTAGGCGGATGCGTCAGTTGGGTGTGAAATCCCGGGGCTTACCCTCGGAACTGCACTCAATACTGCGTATCTGGAGTCCTTCAGGGGTAAGTGGAATTCCATGTGTAGCGGTGAAATGCGTAGATATATGGGAGAACGCCAATGGCGAAGGCAGCTTACTGGGGAGTGACTGACGCTGAGGAACGAAAGTTAGGGTATCGAACGGG
Bacteria	Proteobacteria	Alphaproteobacteria	Thalassobaculales	Thalassobaculales	Thalassobaculales	8	193	TACGGAGGGGGCAAGCGTTGTTCGGAATTACTGGGCGTAAAGGGCGCGTAGGCGGCCGGTCGCGTCAGGCGTGAAAGCCCCGGGCTCAACCTGGGAACTGCGCTTGATACGGGCTGGCTTGAGATCGGAAGAGGAGTGTGGAATTCCCAGTGTAGAGGTGAAATTCGTAGATATTGGGAAGAACACCAGTGG

Kingdom	Phylum	Class	Order	Family	Genus	Blank Total (n=1)	Sample Total (n=56)	Amplicon Sequence Variants (ASVs)
								CGAAGGCGGCACTCTGGTCCGTATCTGACGCT GAGGCGCGAAAGCGTGGGGAGCAAACAGG
Bacteria	Bacteroidetes	Bacteroidia	Bacteroidales	Marinilabiliace ae	Labiliba cter	8	26	TACGGAGGATGCGAGCGTTATCCGGATTTATT GGGTTTAAAGGGTGCGTAGGCGGACTAATAAG TCAGTGGTGAAATCTTGCGGCTCAACCGTAAA ACTGCCATTGATACTGTTAGTCTTGAATTTAGT TGAGGTAGGCGGAATGTGTTGTGTAGCGGTGA AATGCATAGATATAACACAGAACACCGATTGC GAAGGCAGCTTACTAAGCTATAATTGACGCTG ATGCACGAAAGCGTGGGTAGCGAACAGG
Bacteria	Proteobacteria	Alphaproteobacteria	Rhodovibrionale s	Fodinicurvatac eae	Fodinicu rvatacea e	8	11	TACGAAGGGGGCAAGCGTTGTTCAGATTTACT GGGCGTAAAGGGCGCGTAGGCGGATAGTCATG TCAGAGGTGAAAGCCCCGGGCTCAACCTGGGA ACTGCCTTTGAAACGGGCTGTCTTGAGGACGG GAGAGGATGGTGGAATACCCAGTGTAGAGGTG AAATTCGTAGATATTGGGTGGAACACCGGTGG CGAAGGCGACCATCTGGCCCGTAACTGACGCT GAGGCGCGAAAGCGTGGGGAGCAAACAGG
Bacteria	Firmicutes	Bacilli	Lactobacillales	Lactobacillace ae	Lactoba cillus	8	2	TACGTAGGTGGCAAGCGTTGTCCGGATTTATTG GGCGTAAAGCGAGCGCAGGCGGAAGAATAAG TCTGATGTGAAAGCCCTCGGCTTAACCGAGGA ACTGCATCGGAAACTGTTTTTCTTGAGTGCAGA AGAGGAGAGTGGAACTCCATGTGTAGCGGTGG AATGCGTAGATATATGGAAGAACACCAGTGGC GAAGGCGGCTCTCTGGTCTGCAACTGACGCTG AGGCTCGAAAGCATGGGTAGCGAACAGG
Bacteria	Bacteroidetes	Bacteroidia	Flavobacteriales	Flavobacteriac eae	Kordia	8	0	TACGGAGGATCCAAGCGTTATCCGGAATCATT GGGTTTAAAGGGTCCGCAGGCTGTTGTTTAAGT CAGAGGTGAAAGTTTGCAGCTCAACTGTAAAA TTGCCTTTGATACTGAATGACTTGAGTTATAAT GAAGTGGTTAGAATATGTAGTGTAGCGGTGAA ATGCATAGATATTACATAGAATACCGATTGCG

Kingdom	Phylum	Class	Order	Family	Genus	Blank Total (n=1)	Sample Total (n=56)	Amplicon Sequence Variants (ASVs)
								AAGGCAGATCACTAATTATATACTGACGCTGAGGGACGAAAGCGTGGGGAGCGAACAGG
Bacteria	Proteobacteria	Gammaproteobacteria	Gammaproteobacteria	Gammaproteobacteria	Gammaproteobacteria	8	0	TACGGAGGGTGCAAGCGTTAATCGGAATTACTGGGCGTAAAGCGCGCGTAGGCGGTTTGATCAGTCAGATGTGAAAGCCCCGGGCTCAACCTGGGAACTGCGTTTGATACTGTCGGACTCGAATGTGGTAGAGGAAGGTGGAATTCCAAGTGTAGCGGTGAAATGCGTAGATATTTGGAGGAATACCGGTGGCGAAGGCGACTACCTGGCCAGATACTGACGCTGAGGTGCGAAAGCGTGGGGAGCAAACAGG
Bacteria	Proteobacteria	Deltaproteobacteria	Myxococcales	Eel-36e1D6	Eel-36e1D6	8	0	GACAGAGGGTGCAAACGTTGTTCGGAATTACTGGGCGTAAAGCGCGTGTAGGCTGTCACTCAAGTCGGGTGTGAAAGCCCAGGGCTCAACCCTGGAAGTGCACTCGAAACTGCGTGACTAGAATCTCGGAGAGGGTAGCGGAATTCTGGGTGTAGAGGTGAAATTCGTAGATATCCAGAGGAACACCGGTGGCGAAGGCGGCTACCTGGACGAGTATTGACGCTGAGACGCGAAAGTGTGGGGAGCAAACAGG
Bacteria	Proteobacteria	Gammaproteobacteria	Cellvibrionales	Cellvibrionaceae	Marinagarivorans	8	0	TACGAGGGGTGCAAGCGTTAATCGGAATTACTGGGCGTAAAGCGCGCGTAGGCGGCTATTCAAGCCAGATGTGAAATCCCAGGGCTTAACCTTGGAACTGCATCCGGAACTGGATAGCTAGAATACAGCAGAGGAGTGTGGAATTTCAGGTGTAGCGGTGAAATGCGTAGAGATCTGAAGGAACATCAGTGGCGAAGGCGACACTCTGGGCTGATATTGACGCTGAGGTGCGAAAGCGTGGGTAGCAAACAGG
Bacteria	Proteobacteria	Alphaproteobacteria	Holosporales	Holosporaceae	Candidatus_Paraholospora	9	240	TACGGGGGGAGCTAGCGTTATTCGGAATTATTGGGCGTAAAGGGTGCGTAGGTTGTTATATAAGTGAATTATTAAATCCCCGGGCTTAACCCTGGAATTGTAATTTATACTGTATGACTAGAATATGGTAGAGGAAAGTGGAATTCCTAGTGTAGAGGTGAAATTCGTAGATATTAGGAGGAACGTCGAAGGCG

Kingdom	Phylum	Class	Order	Family	Genus	Blank Total (n=1)	Sample Total (n=56)	Amplicon Sequence Variants (ASVs)
								AAGGCAGCTTTCTGGGCCATTATTGACACTGAGGCACGAAAGTGTGGGGAGCAAACAGG
Bacteria	Proteobacteria	Alphaproteobacteria	Rhodobacterales	Rhodobacteraceae	Rhodobacteraceae	9	95	TACGGAGGGGGCTAGCGTTGTTCGGAATTACTGGGCGTAAAGCGCGCGTAGGCGGATTGTTAAGTCGGGGGTGAAATCCCGGGGCTCAACCCCGGAACTGCCTCCGATACTGGCAATCTAGAGGTCGAGAGAGGTGAGTGGAATTCCGAGTGTAGAGGTGAAATTCGTAGATATTCGGAGGAACACCAGTGGCGAAGGCGGCTCACTGGCTCGATACTGACGCTGAGGTGCGAAAGCGTGGGGAGCAAACAGG
Bacteria	Firmicutes	Bacilli	Lactobacillales	Leuconostocaceae	Weissella	9	8	TACGTATGTTCCAAGCGTTATCCGGATTTATTGGGCGTAAAGCGAGCGCAGACGGTTATTTAAGTCTGAAGTGAAAGCCCTCAGCTCAACTGAGGAATTGCTTTGGAAACTGGATGACTTGAGTGCAGTAGAGGAAAGTGGAACTCCATGTGTAGCGGTGAAATGCGTAGATATATGGAAGAACACCAGTGGCGAAGGCGGCTTTCTGGACTGTAACTGACGTTGAGGCTCGAAAGTGTGGGTAGCAAACAGG
Bacteria	Actinobacteria	Actinobacteria	Actinomycetales	Actinomycetaceae	Actinomyces	9	3	TACGTAGGGCGCGAGCGTTGTCCGGAATTATTGGGCGTAAAGGGCTTGTAGGCGGTTGGTCGCGTCTGCCGTGAAATTCTCTGGCTTAACTGGGGGCGTGCGGTGGGTACGGGCTGACTTGAGTGCGGTAGGGGAGACTGGAACTCCTGGTGTAGCGGTGGAATGCGCAGATATCAGGAAGAACACCGGTGGCGAAGGCGGGTCTCTGGGCCGTTACTGACGCTGAGGAGCGAAAGCGTGGGGAGCGAACAGG
Bacteria	Proteobacteria	Alphaproteobacteria	Puniceispirillales	SAR116_clade	SAR116_clade	9	0	TACGAAGGGGGCGAGCGTTATTCGGAATTATTGGGCGTAAAGGGCTCGCAGGCTGCTTGAACAGTTAGACGTGAAATCCCCGGGCTCAACCTGGGAACTGCGTTTAATACTAGCAAGCTAGAGAAATAGAGAGGAAAGTGGAACTCCCAGTGTAGAGGTGAAATTCGTAGATATTGGGAAGAACACCAGTGG

Kingdom	Phylum	Class	Order	Family	Genus	Blank Total (n=1)	Sample Total (n=56)	Amplicon Sequence Variants (ASVs)
								CGAAAGCGGCTATCTGGTCTGATTTTGACGCTGAGGCTCGAAAGCGTGGGGAGCAAACAGG
Bacteria	Cyanobacteria	Oxyphotobacteria	Nostocales	Nostocales	Nostocales	9	0	TACGGAGGATGCAAGCGTTATCCGGAATTATTGGGCGTAAAGCGTTCGCAGGCGGCTTTGTAAGTCTGCTGTTAAAGACCAGAGCTTAACTCTTGGCCGGCGGTGGAAACTGCAGAGCTAGAGTACGGTAGGGGTAGAGGGAATTCCCAGTGTAGCGGTGAAATGCGTAGAGATTGGGAAGAACACCGGTGGCGAAAGCGCTCTACTGGGCCGTAACTGACGCTCAGGGACGAAAGCTAGGGTAGCGAAAGGG
Bacteria	Proteobacteria	Gammaproteobacteria	Oceanospirillales	Saccharospirillaceae	Saccharospirillaceae	9	0	TACGGAGGGTGCAAGCGTTACTCGGAATCACTGGGCGTAAAGCGCATGCAGGTGGTTTGTTAAGTCAGATGTGAAAGCCCGGGGCTCAACCTCGGAATTGCATTTGAAACTGGCAGACTAGAGTACAGTAGAGGGTGGCGGAATTTCCTGTGTAGCGGTGAAATGCGTAGAGATGGGAAGGAACATCAGTGGCGAAGGCGACTGCCTGGACTGATACTGACACTGAGGTGCGAAAGCGTGGGGAGCAAACAGG
Bacteria	Bacteroidetes	Bacteroidia	Flavobacteriales	Cryomorphaceae	Cryomorphaceae	9	0	TACGGAGGATGCAAGCGTTATCCGGATTCATTGGGTTTAAAGGGTCCGTAGGCGGGCTTTTAAGTCAGAGGTGAAAGCCTACAGCTTAACTGTAGAACTGCCTTTGATACTGGAAGTCTTGAGTATAGTTGAAGTGGGCGGAATACGTCATGTAGCGGTGAAATGCATAGATATGACGTAGAACACCTATTGCGAAGGCAGCTCACTAAGCTATAACTGACGCTGAGGGACGAAAGCGTGGGGAGCGAACAGG
Bacteria	Proteobacteria	Gammaproteobacteria	Vibrionales	Vibrionaceae	Catenococcus	9	0	TACGGGGGGTCCGAGCGTTAATCGGAATTACTGGGCGTAAAGGGTCTGTAGGCGGATTATTAAGTCAGATGTGAAAGCCCGGGGCTCAACCTCGGAACTGCATTTGAAACTGGCAGACTAGAGTACTGTAGAGGGGGGTAGAATTTCAGGTGTAGCGGTGAAATGCGTAGAGATCTGAAGGAATACCGGTGG

Kingdom	Phylum	Class	Order	Family	Genus	Blank Total (n=1)	Sample Total (n=56)	Amplicon Sequence Variants (ASVs)
								CGAAGGCGGCCCCCTGGACAGATACTGACACTCAGATGCGAAAGCGTGGGGAGCAAACAGG
Bacteria	Actinobacteria	Actinobacteria	Micrococcales	Micrococcaceae	Rothia	9	0	TACGTAGGGCGCGAGCGTTGTCCGGAATTATTGGGCGTAAAGAGCTTGTAGGCGGTTTGTCGCGTCTGCTGTGAAAGGCCGGAGCTTAACTCCGTGTATTGCAGTGGGTACGGGCAGACTAGAGTGCAGTAGGGGAGACTGGAATTCCTGGTGTAGCGGTGGAATGCGCAGATATCAGGAGGAACACCGATGGCGAAGGCAGGTCTCTGGGCTGTAACTGACGCTGAGGAGCGAAAGCATGGGGAGCGAACAGG
Bacteria	Proteobacteria	Alphaproteobacteria	Caulobacterales	Hyphomonadaceae	Algimonas	9	0	TACGGAGGGGGCTAGCGTTGTTCGGAATTACTGGGCGTAAAGCGTGCGTAGGCGGACTGGAAAGTCAGATGTGAAATCCCGGGGCTCAACCCCGGAACTGCATTTGAAACTATCAGTCTAGAGTTCTGGAGAGGTAAGTGGAATTCCTAGTGTAGAGGTGAAATTCGTAGATATTAGGAGGAACACCAGAGGCGAAGGCGGCTTACTGGACAGATACTGACGCTGAGGCATGAAAGTGTGGGGAGCAAACAGG
Bacteria	Proteobacteria	Alphaproteobacteria	Caulobacterales	Hyphomonadaceae	Hyphomonas	10	2768	TACGAAGGGGGCTAGCGTTGTTCGGAATTACTGGGCGTAAAGCGCACGTAGGCGGACTTTTAAGTCAGATGTGAAATCCCGAGGCTCAACCTCGGAACTGCATTTGAAACTGGGAGTCTAGAGATCAGGAGAGGTTAGCGGAATACCGAGTGTAGAGGTGAAATTCGTAGATATTCGGTGGAACACCAGTGGCGAAGGCGGCTAACTGGACTGATACTGACGCTGAGGTGCGAAAGTGTGGGGAGCAAACAGG
Bacteria	Bacteroidetes	Bacteroidia	Chitinophagales	Saprospiraceae	Phaeodactylibacter	10	13	TACGGAGGGTGCAAGCGTTATCCGGAATCACTGGGTTTAAAGGGTGCGCAGGCGGCGCGCTAAGTCAGGGGTGAAATCCCACCGCCCAACGGTGGAACTGCCCTTGATACTGGCGTGCTCGGATTGGCGCGAGGCAGGCGGAACGCGGCATGTAGCGGTGAAATGCTTAGATATGCCGCAGAACACCGATTGC

Kingdom	Phylum	Class	Order	Family	Genus	Blank Total (n=1)	Sample Total (n=56)	Amplicon Sequence Variants (ASVs)
								GTAGGCAGCCTGCCAGAGCCATATCGACGCTGAGGCACGAAAGCGTGGGGAGCGAACAGG
Bacteria	Proteobacteria	Gammaproteobacteria	Pasteurellales	Pasteurellaceae	Haemophilus	10	0	TACGGAGGGTGCGAGCGTTAATCGGAATAACTGGGCGTAAAGGGCACGCAGGCGGTGACTTAAGTGAGGTGTGAAAGCCCCGGGCTTAACCTGGGAATTGCATTTCATACTGGGTCGCTAGAGTACTTTAGGGAGGGGTAGAATTCCACGTGTAGCGGTGAAATGCGTAGAGATGTGGAGGAATACCGAAGGCGAAGGCAGCCCCTTGGGAATGTACTGACGCTCATGTGCGAAAGCGTGGGGAGCAAACAGG
Bacteria	Proteobacteria	Alphaproteobacteria	Rhodobacterales	Rhodobacteraceae	Rhodobacteraceae	10	0	TACGGAGGGGGTTAGCGTTGTTCGGAATTACTGGGCGTAAAGCGCACGTAGGCGGATCGGAAAGTTGGGGGTGAAATCCCGGGGCTCAAACCCGGAACTGCCCTCGATACTGGCAGCCTAGAGGATGAGAGAGGCGAGTGGAATTCCGAGTGTAGAGGTGAAATTCGTAGATATTCGGAGGAACACCAGTGGCGAAGGCGGCTCGCTGGCTCATTTCTGACGCTGAGGTGCGAAAGCGTGGGGAGCAAACAGG
Bacteria	Proteobacteria	Alphaproteobacteria	Puniceispirillales	SAR116_clade	SAR116_clade	10	0	TACGAAGGGGGCGAGCGTTGTTCGGAATTACTGGGCGTAAAGGGAGCGCAGGCGGTTCAATTAGTTAGGCGTGAAAGCCCCGGGCTCAACCTGGGAACTGCGCTTAATACTGTTGAGCTAGAATACAGAAGAGGATAGTGGAATTCCCAGTGTAGAGGTGAAATTCGTAGATATTGGGAAGAACACCGGTGGCGAAAGCGGCTATCTGGTCTGTTATTGACGCTGAGGCTCGAAAGCGTGGGGAGCAAACAGG
Bacteria	Proteobacteria	Alphaproteobacteria	Rhodobacterales	Rhodobacteraceae	Rhodobacteraceae	10	0	TACGGAGGGGGTTAGCGTTGTTCGGAATTACTGGGCGTAAAGCGCACGTAGGCGGACTAGTCAGTCAGAGGTGAAATCCCGAGGCTCAACCTCGGAACTGCCTTTGATACTGCTAGTCTAGAGTTCGAGAGAGGTGAGTGGAATTCCGAGTGTAGAGGTGAAATTCGTAGATATTCGGAGGAACACCAGTGGC

Kingdom	Phylum	Class	Order	Family	Genus	Blank Total (n=1)	Sample Total (n=56)	Amplicon Sequence Variants (ASVs)
								GAAGGCGGCTCACTGGATCGATACTGACGCTG AGGTGCGAAATCGTGGGGAGCAAACAGG
Bacteria	Proteobacteria	Gammaproteobacteria	Betaproteobacteriales	Burkholderiaceae	Aquabacterium	10	0	TACGTAGGGTGCGAGCGTTAATCGGAATTACT GGGCGTAAAGCGTGCGCAGGCGGCTTTGCAAG ACAGATGTGAAATCCCCGGGCTCAACCTGGGA ACTGCATTTGTGACTGCAAGGCTAGAGTACGG CAGAGGGGGATGGAATTCCGCGTGTAGCAGTG AAATGCGTAGATATGCGGAGGAACACCGATGG CGAAGGCAATCCCCTGGGCCTGTACTGACGCT CATGCACGAAAGCGTGGGGAGCAAACAGG
Bacteria	Bacteroidetes	Bacteroidia	Bacteroidia	Bacteroidia	Bacteroidia	10	0	TACGGAGGGTGCAAGCGTTATCCGAATTTATT GGGTTTAAAGGGCATTTAGGCGGTCTTTTAAGT CAGAGGTGAAAGTTCAGGGCTCAACTCTGAAA AGTCCTTTGATACTGTAAGGCTGGAGTGATATG GGCGTAGGCGGAATTTGAGGTGTAGCGGTGAA ATGCATAGATCTCTCAAGGAATACCGAAAGCG AAGGCAGCTTACGATGTATCAACTGACGCTGA AGTTCGAAAGCGTGGGTAGCGAATGGG
Bacteria	Cyanobacteria	Oxyphotobacteria	Limnotrichales	Limnotrichaceae	Limnothrix	10	0	GACGGAGGATGCAAGCGTTATCCGGAATTATT GGGCGTAAAGGGTCCGCAGGTGGCTATTCAAG TCTGCTTTCAAAGAGTGGAGCTCAACTCCGTAG AGGGAGTGGAAACTGAGTAGCTAGAGTAAGG AAGGGGTAAGAGGAATTCCCAGTGTAGCGGTG AAATGCGTAGATATTGGGAAGAACACCAGCAG CGAAGGCGTCTTACTGGTCCTTAACTGACACTG AGGGACGAAAGCTAGGGGAGCGAAAGGG
Bacteria	Proteobacteria	Alphaproteobacteria	Sphingomonadales	Sphingomonadaceae	Altererythrobacter	10	0	TACGGAGGGAGCTAGCGTTGTTCGGAATTACT GGGCGTAAAGCGCGCGTAGGCGGCGATCCAAG TCAGGGGTGAAATCCCGGGGCTCAACCCCGGA ACTGCCCTTGAAACTAGATTGCTAGAATATTGG AGAGGTAAGTGGAATTCCGAGTGTAGAGGTGA AATTCGTAGATATTCGGAAGAACACCAGTGGC

Kingdom	Phylum	Class	Order	Family	Genus	Blank Total (n=1)	Sample Total (n=56)	Amplicon Sequence Variants (ASVs)
								GAAGGCGACTTACTGGACAATTATTGACGCTGAGGTGCGAAAGCGTGGGGAGCAAACAGG
Bacteria	Actinobacteria	Actinobacteria	Micrococcales	Microbacteriaceae	Microbacteriaceae	10	0	TACGTAGGGCGCAAGCGTTGTCCGGAATTATTGGGCGTAAAGAGCTCGTAGGCGGTCTGTCGCGTCTGCTGTGAAATCCCGAGGCTCAACCTCGGGCTTGCAGTGGGTACGGGCAGACTAGAGTGCGGTAGGGGAGAATGGAATTCCTGGTGTAGCGGTGGAATGCGCAGATATCAGGAGGAACACCGATGGCGAAGGCAGTTCTCTGGGCCGTAACTGACGCTGAGGAGCGAAAGCGTGGGGAGCGAACAGG
Bacteria	Proteobacteria	Alphaproteobacteria	Thalassobaculales	Thalassobaculales	Thalassobaculales	11	14	TACGGAGGGGGCAAGCGTTGTTCGGAATTACTGGGCGTAAAGGGCGCGTAGGCGGCGCACTGCGTCAGGTGTGAAATCCCCGGGCTCAACCCGGGAACTGCACTTGATACGGGTGTGCTTGAGACCGGAAGAGGTGAGTGGAATTCCCAGTGTAGAGGTGAAATTCGTAGATATTGGGAAGAACACCAGTGGCGAAGGCGGCTCACTGGTCCGGTTCTGACGCTGAGGCGCGAAAGCGTGGGGAGCAAACAGG
Bacteria	Proteobacteria	Alphaproteobacteria	Acetobacterales	Acetobacteraceae	Craurococcus	11	0	TACGAAGGGGGCTAGCGTTGCTCGGAATCACTGGGCGTAAAGGGCGCGTAGGCGGCGGCTCTAGTCGGGCGTGAAATCCCCGGGCTCAACCCGGGGGCTGCGCCCGATACGGGGCCGCTCGAGGCAAGGAGAGGCTCGCGGAATTCCCAGTGTAGAGGTGAAATTCGTAGATATTGGGAAGAACACCGGTGGCGAAGGCGGCGAGCTGGCCTTGATCTGACGCTGATGCGCGACAGCGTGGGGAGCAAACAGG
Bacteria	Proteobacteria	Gammaproteobacteria	Xanthomonadales	Xanthomonadaceae	Stenotrophomonas	11	0	TACGAAGGGTGCAAGCGTTACTCGGAATTACTGGGCGTAAAGCGTGCGTAGGTGGTTGTTTAAGTCTGTTGTGAAAGCCCTGGGCTCAACCTGGGAACTGCAGTGGAAACTGGACGACTAGAGTGTGGTAGAGGGTAGCGGAATTCCTGGTGTAGCAGTGAAATGCGTAGAGATCAGGAGGAACATCCATGG

Kingdom	Phylum	Class	Order	Family	Genus	Blank Total (n=1)	Sample Total (n=56)	Amplicon Sequence Variants (ASVs)
								CGAAGGCAGCTACCTGGACCAACACTGACACTGAGGCACGAAAGCGTGGGGAGCAAACAGG
Bacteria	Proteobacteria	Alphaproteobacteria	Kordiimonadales	Kordiimonadales_Incertae_Sedis	Eilatimonas	11	0	TACGGAGGGAGCTAGCGTTGTTCGGAATTACTGGGCGTAAAGTGCGCGTAGGCGGCTTAGCAAGTTGGGGGTGAAAGCCCGGGGCTCAACCCCGGAACTGCCCTCAAAACTGCTAAGCTAGAGATTTGGAGAGGTGAGTGGAATTCCTAGTGTAGAGGTGAAATTCGTAGATATTAGGAAGAACACCAGTGGCGAAGGCGGCTCACTGGCCAAATACTGACGCTGAGGTGCGAAAGCGTGGGGAGCAAACAGG
Bacteria	Proteobacteria	Gammaproteobacteria	Oceanospirillales	Saccharospirillaceae	Thalassolituus	11	0	TACGGAGGGTGCGAGCGTTAATCGGAATTACTGGGCGTAAAGCGCATGCAGGCGGTTAGTTAAGTCAGATGTGAAAGCCCCGGGCTCAACCTGGGAACTGCATTGCGAACTGGCAAGCTAGAGTACAGTAGAGGGTAGTGGAATTTCCTGTGTAGCGGTGAAATGCGTAGAGATGGGAAGGAACATCAGTGGCGAAGGCGACTGCCTGGACTGATACTGACACTGAGGTGCGAAAGCGTGGGGAGCAAACAGG
Bacteria	Proteobacteria	Gammaproteobacteria	Alteromonadales	Pseudoalteromonadaceae	Algicola	11	0	TACGGAGGGTGCGAGCGTTAATCGGAATTACTGGGCGTAAAGCGTACGCAGGCGGTTGATTAAGTCAGATGTGAAAGCCCCGGGCTCAACCTGTGAACTGCATTTGAAACTGGTCAACTAGAGTGCGGTAGAGGGTGGTAGAATTTCAGGTGTAGCGGTGAAATGCGTAGAGATCTGAAGGAATACCGATGGCGAAGGCAGCCACCTGGACCGACACTGACGCTCATGTACGAAAGCGTGGGTAGCAAACAGG
Bacteria	Proteobacteria	Alphaproteobacteria	Puniceispirillales	SAR116_clade	SAR116_clade	11	0	TACGAAGGGGGCGAGCGTTGTTCGGAATTACTGGGCGTAAAGGGCGCGCAGGCGGCCAAATCAGTTAGGTGTGAAAGCCCCGGGCTTAACCTGGGAACTGCACTTAATACTGTTTGGCTAGAAAACGGAAGAGGAGAGTGGAATTCCCAGTGTAGAGGTGAAATTCGTAGATATTGGGAAGAACACCAGTG

Kingdom	Phylum	Class	Order	Family	Genus	Blank Total (n=1)	Sample Total (n=56)	Amplicon Sequence Variants (ASVs)
								GCGAAAGCGTCTCTCTGGTCCGATTTTGACGCTGAGCCGCGAAAGCGTGGGGAGCAAACAGG
Bacteria	Proteobacteria	Alphaproteobacteria	Rickettsiales	Rickettsiaceae	Rickettsiaceae	12	78	TACGGGGGGAGCTAGCGTTTTTCGGATTTACTGGGCGTAAAGGGTGTGTAGGCGGTTAATAAAGTTGAAAGTGAAATCCTCAGGCTTAACCTGAGAACTGCTTTCAAAACTCATTGACTAGAGAACGATAGAGGATAACAGAATTCCTAGTGTAGAGGTGAAATTCTTAGATATTAGGAGGAATACCGGTGGCGAAGGCGGTTATCTGGGTCGTTTCTGACGCTGAGGCACGAAAGCGTGGGGAGCAAACAGG
Bacteria	Proteobacteria	Alphaproteobacteria	Rhodobacterales	Rhodobacteraceae	Rhodobacteraceae	12	24	TACGGAGGGGGCTAGCGTTGTTCGGAATTACTGGGCGTAAAGCGCGCGTAGGCGGACCTTTAAGTCGGGGGTGAAATCCCGGGGCTCAACCCCGGAACTGCCTTCGATACTGGGGGTCTAGAGGTCTGGAGAGGCGAGTGGAATGTCGAGTGTAGAGGTGAAATTCGTAGATATTCGACGGAACACCAGTGGCGAAGGCGGCTCGCTGGCCAGATACTGACGCTGAGGTGCGAAAGCGTGGGGAGCAAACAGG
Bacteria	Proteobacteria	Gammaproteobacteria	Oceanospirillales	Nitrincolaceae	Nitrincolaceae	12	9	TACGGAGGGTGCGAGCGTTAATCGGAATTACTGGGCGTAAAGCGCGCGTAGGCTGATAGGTCAGTCAGATGTGAAAGCCCCGGGCTCAACCTGGGAACTGCACCTGATACTGCCTATCTAGAGTACGGTAGAGGGTAGTGGAATTTCCTGTGTAGCGGTGAAATGCGTAGATATAGGAAGGAACACCAGTGGCGAAGGCGACTACCTGGACCGATACTGACGCTGAGGTGCGAAAGCGTGGGGAGCAAACAGG
Bacteria	Bacteroidetes	Bacteroidia	Flavobacteriales	Flavobacteriaceae	NS5_marine_group	12	7	TACGGAGGATCCAAGCGTTATCCGGAATCATTGGGTTTAAAGGGTCCGTAGGTGGTCTATTAAGTCAGAGGTGAAAGCCTATCGCTCAACGATAGAACTGCCTTTGATACTGATGGACTTGAGTTATTGTGAAGTAGTTAGAATATGTAGTGTAGCGGTGAAATGCATAGATATTACATAGAATACCGATTGC

Kingdom	Phylum	Class	Order	Family	Genus	Blank Total (n=1)	Sample Total (n=56)	Amplicon Sequence Variants (ASVs)
								GAAGGCAGATTACTAACAATATACTGACACTG AGGGACGAAAGCGTGGGTAGCGAACAGG
Bacteria	Actinobacteria	Acidimicrobiia	Microtrichales	Microtrichaceae	Sva0996_marine_group	12	0	TACGTAGGGGGCGAGCGTTGTCCGGAATTATT GGGCGTAAAGAGCTCGTAGGCGGTTCGGTAAG TCGGGTGTGAAAATCCAGGGCTCAACCCTGGG ACGCCACTCGATACTGCTGTGACTAGAGTCCG GTAGAGGAGCGTGGAATTCCTGGTGTAGCGGT GAAATGCGCAGATATCAGGAGGAACACCAGCG GCGAAGGCGGCGCTCTGGGCCGGTACTGACGC TGAGGTGCGAAAGCGTGGGGAGCAAACAGG
Bacteria	Cyanobacteria	Oxyphotobacteria	Phormidesmiales	Phormidesmiaceae	Acrophormium_PCC-7375	12	0	TACGGAGGGTGCAAGCGTTGTCCGGATTTATT GGGCGTAAAGCGTCCGTAGGCGGTTAATTAAG TCAGTTGTTAAAGCCTGCAGCTCAACTGTGGA GGAGCAACTGAAACTGGTTAACTAGAGTATGG TAGGGGTAGAGGGAATTCCTAGTGTAGCGGTG AAATGCGTAGATATTAGGAAGAACACCAGTGG CGAAGGCGCTCTACTGGGCCAAGACTGACGCT GATGGACGAAAGCTAGGGGAGCGAAAGGG
Bacteria	Spirochaetes	Spirochaetia	Spirochaetales	Spirochaetaceae	Sediminispirochaeta	12	0	CACGTAAGGGGCGAGCGTTGTTCGGATTTATT GGGCGTAAAGGGCGCGTAGGCGGTGAGGTAA GTCTGGTGTTAAATACTTCGGCTCAACCGGAG GGGTGCGCTGGAAACTGTCTTGCTTGAGTGAT GGAGGGGGAACTGGAATTCCTGGTGTAGGGGT GAAATCTGTAGAGATCAGGAAGAACACCGGAG GCGAAGGCGAGTTCCTGGCCATCTACTGACGC TGAGGCGCGAAAGCGTGGGGAGCAAACGGG
Bacteria	Bacteroidetes	Bacteroidia	Cytophagales	Cyclobacteriaceae	Cyclobacteriaceae	12	0	TACGGAGGGTGCAAGCGTTGTCCGGATTTACT GGGTTTAAAGGGTACGTAGGCGGCTTATTAAG TCAGTGGTGAAATGCACGAGCTCAACTGGTGA AGTGCCATTGATACTGATAGGCTTGAGTATTGT AAAGGTAGGCGGAATTGATGGTGTAGCGGTGA AATGCATAGATACCATCAAGAACACCAATTGC

Kingdom	Phylum	Class	Order	Family	Genus	Blank Total (n=1)	Sample Total (n=56)	Amplicon Sequence Variants (ASVs)
								GAAGGCAGCTTACTGGCAAATACTGACGCTGAGGTACGAAAGCGTGGGGAGCGAACAGG
Bacteria	Proteobacteria	Alphaproteobacteria	Rhodobacterales	Rhodobacteraceae	Ruegeria	13	14954	TACGGAGGGGGTTAGCGTTGTTCGGAATTACTGGGCGTAAAGCGCACGTAGGCGGATCGGAAAGTTGGGGGTGAAATCCCGGGGCTCAACCCCGGAACTGCCTCCAAAACTATCGGTCTAGAGTTCGAGAGAGGTGAGTGGAATTCCGAGTGTAGAGGTGAAATTCGTAGATATTCGGAGGAACACCAGTGGCGAAGGCGGCTCACTGGCTCGATACTGACGCTGAGGTGCGAAAGTGTGGGGAGCAAACAGG
Bacteria	Proteobacteria	Deltaproteobacteria	Desulfobacterales	Desulfobulbaceae	Desulfobulbaceae	13	2097	TACGGAGGGTGCAAGCGTTGTTCGGAATTACTGGGCGTAAAGCGCGCGTAGGCGGCTATCTAAGTCAGATGTGAAAGCCCGCGGCTCAACCGTGGAAGTGCATTTGAAACTGGGTAGCTTGAGTACTGGAGGGGGTAGTGGAATTCCCGGTGTAGAGGTGAAATTCGTAGATATCGGGAGGAATACCGGTGGCGAAGGCGACTACCTGGCCAGATACTGACGCTGAGGTGCGAAAGCGTGGGGAGCAAACAGG
Bacteria	Proteobacteria	Alphaproteobacteria	Parvibaculales	Parvibaculaceae	Parvibaculaceae	13	196	TACGGAGGGGGCTAGCGTTGTTCGGAATTACTGGGCGTAAAGCGCGCGTAGGCGGATTAGTAAGTCAGTGGTGAAAGCCCGGGGCTCAACCTCGGAACTGCCTTTGATACTGCTAGTCTTGAGTTCGAGAGAGGTGAGTGGAATTTCTAGTGTAGAGGTGAAATTCGTAGATATTAGAAAGAACACCAGTGGCGAAGGCGGCTCACTGGCTCGATACTGACGCTGAGGTGCGAAAGCGTGGGGAGCAAACAGG
Bacteria	Bacteroidetes	Bacteroidia	Flavobacteriales	Crocinitomicaceae	Crocinitomix	13	85	TACGGAGGGTCCAAGCGTTATCCGGAATCATTGGGTTTAAAGGGTCCGCAGGCGGTTTGTTAAGTCAGTGGTGAAATCCTATCGCTCAACGATAGAACTGCCATTGATACTGACAGACTTGAATTTAGTTGGAGTAGGCGGAATGTGTAGTGTAGCGGTGAAATGCTTAGATATTACACAGAACACCGATAGC

Kingdom	Phylum	Class	Order	Family	Genus	Blank Total (n=1)	Sample Total (n=56)	Amplicon Sequence Variants (ASVs)
								GAAGGCAGCTTACTAAACTAATATTGACGCTC ATGGACGAAAGCGTGGGGAGCGAACAGG
Bacteria	Bacteroidetes	Bacteroidia	Chitinophagales	Saprospiraceae	Saprospi raceae	13	78	TACGGAGGGTGCAAGCGTTATCCGGAATCACT GGGTTTAAAGGGTGCGTAGGCGGCGTTATAAG TCAGAAGTGAAAGTTGGCAGCTTAACTGCAAA ATTGCTTTTGATACTGTAGTGCTTGAATCAAGT TGAGGCTGGCGGAATGTGGCATGTAGCGGTGA AATGCATAGATATGCCATAGAACACCAATTGC GAAGGCAGCTGGCTAAGCTTGTATTGACGCTG AGGCACGAAAGCGTGGGGAGCGAACAGG
Bacteria	Proteobacteria	Gammaproteobacteria	Gammaproteoba cteria	Gammaproteo bacteria	Gamma proteoba cteria	13	0	TACGGAGGGTGCAAGCGTTAATCGGAATTACT GGGCGTAAAGCGCGCGTAGGCGGCTTATTAAG TCTGATGTGAAAGCCCCGGGCTCAACCTGGGA ACTGCATTGGATACTGGTAGGCTAGAATATGT CAGAGGAAAGCGGAATTCCGGGTGTAGCGGTG AAATGCGTAGATATCCGGAGGAACATCAATGG CGAAGGCAGCTTTCTGGGACAATATTGACGCT GAGGTGCGAAAGCGTGGGGAGCAAACAGG
Bacteria	Bacteroidetes	Bacteroidia	Flavobacteriales	Flavobacteriac eae	Flavoba cteriacea e	13	0	TACGGAGGATGCAAGCTTTATCCGGAATCATT GGGTTTAAAGGGTCTGTAGGCGGTTTGTTAAGT TAGTAGTGAAAGTTTGTGGCTCAACCATAAAA TTGCTATTAAAACTGATAGACTTGAGTTACAGT GAAGTAAGTAGAATGTGTAGTGTAGAGGTAAA ATTCATAGATATTACACAGAATACCGATTGCG AAGGCAGCTTACTAACTGTATACTGACTCTGA GGGACGAAAGCGTGGGGAGCGAACAGG
Bacteria	Proteobacteria	Alphaproteobacteria	Rhodobacterales	Rhodobacterac eae	HIMB1 1	13	0	TACGAAGGGGGCAAGCGTTGTTCGGAATTACT GGGCGTAAAGCGCGCGTAGGCGGATTAGAAAG TTGGGGGTGAAATCCCGGGGCTCAACCTCGGA ACTGCCTCCAAAACTGCTAGTCTAGAGTTCGA GAGAGGTGAGTGGAATTCCGAGTGTAGAGGTG AAATTCGTAGATATTCGGAGGAACACCAGTGG

Kingdom	Phylum	Class	Order	Family	Genus	Blank Total (n=1)	Sample Total (n=56)	Amplicon Sequence Variants (ASVs)
								CGAAGGCGGCTCACTGGCTCGATACTGACGCTGTTGCACGAAAGCGTGGGGAGCAAACAGG
Bacteria	Bacteroidetes	Bacteroidia	Flavobacteriales	Flavobacteriales	Flavobacteriales	13	0	TACGGAGGATTCGAGCGTTATCCGGATTCATTGGGTTTAAAGGGTCTGTAGGCGGATTTATAAGTCAGTGGTGAAATCCCGAGGCTCAACCTCGGAACTGCCATTGAAACTGTAAGTCTAGAGTGTGATAGAAGTTGGCGGAATGTGTGGTGTAGCGGTGAAATGCATAGAGATCACACAGAACACCAATTGCGAAGGCAGCTGACTATGTATATACTGACGCTGAGAGACGAAAGCGTGGGGAGCGAACAGG
Bacteria	Proteobacteria	Alphaproteobacteria	Puniceispirillales	SAR116_clade	SAR116_clade	13	0	TACGAAGGGGGCGAGCGTTGTTCGGAATTACTGGGCGTAAAGGGCGCGTAGGCGGCTCTTTAAGTTATGCGTGAAAGCCCCGGGCTCAACCTGGGAACTGCGCTTAAGACTGGAGAGCTAGAAAACGGAAGAGGGTAGTGGAATTCCCAGTGTAGAGGTGAAATTCGTAGATATTGGGAAGAACACCAGTGGCGAAAGCGGCTACCTGGTCCGGATTTGACGCTGAGGCGCGAAAGCGTGGGGAGCAAACAGG
Bacteria	Proteobacteria	Gammaproteobacteria	HOC36	HOC36	HOC36	13	0	TACGGAGGGTGCGAGCGTTAATCGGAATTACTGGGCGTAAAGCGCGCGTAGGCGGCGTGTTAAGTCAGCTGTGAAAGCCCCGGGCTTAACCTGGGAATGGCAGTTGATACTGGCAGGCTAGAGTGTGATAGAGGACAGTGGAATTCCCGGTGTAGCGGTGAAATGCGTAGAGATCGGGAGGAACACCGATGGCGAAGGCAGCTGTCTGGGTCGACACTGACGCTGAGGTGCGAAAGCGTGGGGAGCAAACAGG
Bacteria	Bacteroidetes	Bacteroidia	Cytophagales	Flammeovirgaceae	Flammeovirgaceae	13	0	TACGGAGGGTGCAAGCGTTGTCCGGATTTATTGGGTTTAAAGGGTACGTAGGCGGTTTGATAAGTCAGTGGTGAAATTTTGCAGCTTAACTGTAAAAGTGCCATTGATACTGTCAGACTAGAATTTTGTAGAGGTGGACGGAATTCCTCATGTAGCGGTGAAATGCATAGATATGAGGAGGAACGCCGACAGCG

Kingdom	Phylum	Class	Order	Family	Genus	Blank Total (n=1)	Sample Total (n=56)	Amplicon Sequence Variants (ASVs)
								AAGGCAGTTCACTGGGCAATAATTGACGCTGAGGTACGAAAGCGTGGGGAGCGAACAGG
Bacteria	Proteobacteria	Gammaproteobacteria	Oceanospirillales	Saccharospirillaceae	Oleibacter	13	0	TACGGAGGGTGCAAGCGTTATTCGGAATCACTGGGCGTAAAGCGTACGTAGGTTGTTTGCTAAGCGAGATGTGAAAGCCCCGGGCTTAACCTGGGAACTGCATTTCGAACTGGCAAGCTAGAGTACAGTAGAGGGTGGCGGAATTTCCTGTGTAGCGGTGAAATGCGTAGAGATGGGAAGGAACATCAGTGGCGAAGGCGGCCACCTGGACTGATACTGACACTGAGGTGCGAAAGCGTGGGTAGCGAACAGG
Bacteria	Actinobacteria	Actinobacteria	Bifidobacteriales	Bifidobacteriaceae	Bifidobacterium	13	0	TACGTAGGGTGCAAGCGTTATCCGGAATTATTGGGCGTAAAGAGCTCGTAGGCGGTCCGTCGCGTCTGGTGTGAAAGTCCATCGCTTAACGGTGGATCTGCGCCGGGTACGGGCGGGCTGGAGTGCGGTAGGGGAGACTGGAATTCCCGGTGTAACGGTGGAATGTGTAGATATCGGGAAGAACACCAATGGCGAAGGCAGGTCTCTGGGCCGTTACTGACGCTGAGGAGCGAAAGCGTGGGGAGCGAACAGG
Bacteria	Proteobacteria	Alphaproteobacteria	Rhodobacterales	Rhodobacteraceae	HIMB11	14	1363	TACGGAGGGGGTTAGCGTTGTTCGGAATTACTGGGCGTAAAGCGCGCGTAGGCGGATTAGAAAGTTGGGGGTGAAATCCCGGGGCTCAACCTCGGAACTGCCTCCAAAACTGCTAGTCTAGAGTTCGAGAGAGGTGAGTGGAATTCCGAGTGTAGAGGTGAAATTCGTAGATATTCGGAGGAACACCAGTGGCGAAGGCGGCTCACTGGCTCGATACTGACGCTGAGGTGCGAAAGTGTGGGGAGCAAACAGG
Bacteria	Proteobacteria	Gammaproteobacteria	SAR86_clade	SAR86_clade	SAR86_clade	14	1016	TACGGAGGGTCCAAGCGTTAATCGGAATTACTGGGCGTAAAGCGCGCGTAGGCGGTTTGTCAAGTTGGATGTGAAATCCCTGGGCTCAACCTAGGAACTGCATTCAAAACTTATAGACTAGAGTACGATAGAGGAGAGTAGAATTCCTGGTGTAGCGGTGAAATGCGTAGATATCAGGAGGAATACCAATGG

Kingdom	Phylum	Class	Order	Family	Genus	Blank Total (n=1)	Sample Total (n=56)	Amplicon Sequence Variants (ASVs)
								CGAAGGCAACTCTCTGGATCGACACTGACGCTGAGGTGCGAAAGCGTGGGTAGCAAACAGG
Bacteria	Proteobacteria	Alphaproteobacteria	Rhodobacterales	Rhodobacteraceae	Rhodobacteraceae	14	477	TACGGAGGGGGTTAGCGTTGTTCGGAATTACTGGGCGTAAAGCGCGCGTAGGCGGATTGGAAAGTTAGGGGTGAAATCCCAGGGCTCAACCCTGGAACTGCCCTTAAAACTCCCAGTCTAGAGTTCGAGAGAGGTGAGTGGAATTCCGAGTGTAGAGGTGAAATTCGTAGATATTCGGAGGAACACCAGTGGCGAAGGCGGCTCACTGGCTCGATACTGACGCTGAGGTGCGAAAGTGTGGGGAGCAAACAGG
Bacteria	Cyanobacteria	Oxyphotobacteria	Nostocales	Geitlerinemaceae	Geitlerinema_PCC-9228	14	370	TACGGAGGAGGCAAGCGTTATCCGGAATCATTGGGCGTAAAGCGTTCGTAGGGGGCATTACAAGTCTGCTGTCAAAGGTCGGGGCTCAACTCCGGAAAGGCAGTGGAAACTGTAAAGCTAGAGGGCGGTAGGGGCAAAGGGAATTCCCGGTGTAGCGGTGAAATGCGTAGATATCGGGAAGAACACCGGTGGCGAAAGCGCTTTGCTGGGCCGCACCTGACCCTGAGGGACGAAAGCTAGGGGAGCGAATGGG
Bacteria	Firmicutes	Negativicutes	Selenomonadales	Veillonellaceae	Veillonella	14	16	TACGTAGGTGGCAAGCGTTGTCCGGAATTATTGGGCGTAAAGCGCGCGCAGGCGGATAGGTCAGTCTGTCTTAAAAGTTCGGGGCTTAACCCCGTGATGGGATGGAAACTGCCAATCTAGAGTATCGGAGAGGAAAGTGGAATTCCTAGTGTAGCGGTGAAATGCGTAGATATTAGGAAGAACACCAGTGGCGAAGGCGACTTTCTGGACGAAAACTGACGCTGAGGCGCGAAAGCCAGGGGAGCGAACGGG
Bacteria	Actinobacteria	Actinobacteria	Micrococcales	Micrococcaceae	Kocuria	14	0	TACGTAGGGCGCAAGCGTTGTCCGGAATTATTGGGCGTAAAGAGCTCGTAGGCGGTTTGTCGCGTCTGCTGTGAAAGCCCGGGGCTTAACCCCGGGTGTGCAGTGGGTACGGGCAGACTTGAGTGCAGTAGGGGAGACTGGAATTCCTGGTGTAGCGGTGAAATGCGCAGATATCAGGAGGAACACCGATGG

Kingdom	Phylum	Class	Order	Family	Genus	Blank Total (n=1)	Sample Total (n=56)	Amplicon Sequence Variants (ASVs)
								CGAAGGCAGGTCTCTGGGCTGTTACTGACGCTGAGGAGCGAAAGCATGGGGAGCGAACAGG
Bacteria	Proteobacteria	Alphaproteobacteria	Micavibrionales	Micavibrionaceae	Micavibrionaceae	14	0	TACGAATGGAGCAAGCGTTGTTCGGAATCACTGGGCTTAAAGCGTATGTAGGCGGACTTGAAAGTCAGGAGTGAAATCCCAGGGCTCAACCCTGGAACTGCTCTTGAAACTCCAAGTCTAGAATCCTGGAGAGGTTAGGAGAATTCCGAGTGTAGAGGTGAAATTCGCAGATATTCGGAGGAATACCAGTGGCGTAGGCGCCTAACTGGACAGGTATTGACGCTCAGATACGAAAGCGTGGGGAGCAAACAGG
Bacteria	Proteobacteria	Gammaproteobacteria	Chromatiales	Chromatiales	Chromatiales	14	0	TACGGAGGGTGCAAGCGTTAATCGGAATTACTGGGCGTAAAGCGCGCGTAGGCGGTTAGGTCAGTCAGATGTGAAATCCCCGGGCTCAACCTGGGAACTGCATTTGATACTGCCTGACTAGAGTGTGGTAGAGGGAAGTGGAATTCCAGGTGTAGCGGTGAAATGCGTAGATATCTGGAGGAACACCAGTGGCGAAGGCGACTTCCTGGACCAACACTGACGCTGAGGTGCGAAAGCGTGGGTAGCAAACAGG
Bacteria	Proteobacteria	Gammaproteobacteria	Vibrionales	Vibrionaceae	Catenococcus	14	0	TACGGAGGGTGCGAGCGTTAATCGGAATTACTGGGCGTAAAGCGCATGCAGGTGGTTTGTTAAGTCAGATGTGAAAGCCCGGGGCTCAACCTCGGAATTGCATTTGAAACTGGCAGACTAGAGTACAGTAGAGGGTAGTGGAATTTCCTGTGTAGCGGTGAAATGCGTAGAGATGGGAAGGAACATCAGTGGCGAAGGCGACTGCCTGGACAGATACTGACACTCAGATGCGAAAGCGTGGGGAGCAAACAGG
Bacteria	Bacteroidetes	Bacteroidia	Flavobacteriales	Flavobacteriaceae	Flavobacteriaceae	14	0	TACGGAGGATCCAAGCGTTATCCGGAATCATTGGGTTTAAAGGGTCCGTAGGTGGATAATTAAGTCAGAGGTGAAAGTCTGCAGCTCAACTGTAGAATTGCCTTTGATACTGGTTATCTTGAGTCATTGTGAAGTAGTTAGAATATGTAGTGTAGCGGTGAAATGCATAGATATTACATAGAATACCAATTGC

Kingdom	Phylum	Class	Order	Family	Genus	Blank Total (n=1)	Sample Total (n=56)	Amplicon Sequence Variants (ASVs)
								GAAGGCAGATTACTAACAATGTACTGACACTGATGGACGAAAGCGTGGGGAGCAAACAGG
Bacteria	Proteobacteria	Alphaproteobacteria	Rhodobacterales	Rhodobacteraceae	Ascidiaceihabitans	14	0	TACGGAGGGGGTTAGCGTTGTTCGGAATTACTGGGCGTAAAGCGCGCGTAGGCGGATTGGAAAGTTAGAGGTGAAATCCCGGGGCTCAACCCCGGAACTGCCTTTAAAACTCCCAGTCTTGAGTTCGAGAGAGGTGAGTGGAATTCCAAGTGTAGAGGTGAAATTCGTAGATATTTGGAGGAACACCAGTGGCGAAGGCGGCTCACTGGCTCGATACTGACGCTGAGGTGCGAAAGTGTGGGGAGCAAACAGG
Bacteria	Proteobacteria	Alphaproteobacteria	Rhodobacterales	Rhodobacteraceae	Rhodobacteraceae	14	0	TACGGAGGGGGTTAGCGTTGTTCGGAATTACTGGGCGTAAAGCGCGCGTAGGCGGATTAGAAAGTTGGGGGTGAAATCCCGGGGCTCAACCTCGGAACTGCCTCCAAAACTGCTAGTCTAGAGTTCGAGAGAGGTGAGTGGAATTCCGAGTGTAGAGGTGAAATTCGTAGATATTCGGAGGAAAACCAATTGCGAAGGCAGCTCACTGGGCCGCTATCGACGCTGAGGAGCGAAAGCTAGGGGAGCAAACAGG
Bacteria	Proteobacteria	Gammaproteobacteria	UBA10353_marine_group	UBA10353_marine_group	UBA10353_marine_group	14	0	TACGGAGGGTGCAAGCGTTAATCGGAATCACTGGGCGTAAAGCGCACGCAGGCGGTTTGTTAAGTCAGATGTGAAAGCCCCGGGCTCAACCTGGGAACTGCATTTGATACTGGCAAACTAGAGTATGGTAGAGAGAAGTAGAATTCCACATGTAGCGGTGAAATGCGTAGAGATGTGGAGGAATATCAGTGGCGAAGGCGACTTCTTGGACCAATACTGACGCTCAGGTGCGAAAGCGTGGGGAGCAAACAGG
Bacteria	Patescibacteria	Gracilibacteria	JGI_0000069-P22	JGI_0000069-P22	JGI_0000069-P22	15	22	TACGTATGTCTCAAATGTTACCCGGAATCACTGGGTGTAAAGCGTTTGTAGGCGGTTTAATAAGTTGGATATGAAAGACCAAGGCTCAACCTTGTGTTTGTATTCAAAACTGTTAAACTAGAATTAGGGAGAGGTAAGCGGAATTCTGAGTGTAGGGGTGCAATCCGTAGATACTCAGAGGAACACCAAAAGCG

Kingdom	Phylum	Class	Order	Family	Genus	Blank Total (n=1)	Sample Total (n=56)	Amplicon Sequence Variants (ASVs)
								AAGGCAGCTTACTGGAACTATATTGACGCTCA TAAACGAAAGCGTGGGTAGCGAAAAGG
Bacteria	Proteobacteria	Gammaproteobacteria	Gammaproteobacteria	Gammaproteobacteria	Gammaproteobacteria	15	3	TACGGAGAGGGCTAGCGTTGTTCGGAATTATT GGGCGTAAAGAGTACGCAGGTGGTTATGAAAG TTAACTGTGAAATCCCTAAGCTTAACTTAGGAA CTGCAGCTAATACTACATGACTTGAGTGTGAA AGAGGATAGTAGAATTTCTAGTGTAGCGGTAA AATGCGTAGATATTAGAAGGAATACCAACAGC GAAGGCAGCTATCTGGTTCACTACTGACACTG AGGTACGAAAGCATGGGGAGCAAACAGG
Bacteria	Cyanobacteria	Oxyphotobacteria	Nostocales	Microcystaceae	Crocosphaera_WH_0003_(UCYN-B)	15	0	TACGGAGGATGCAAGCGTTATCCGGAATTATT GGGCGTAAAGCGTCCGCAGGTGGTCTTTCAAG TCTGCTGTTAAATACCAGAGCTTAACTCTGGGC CGGCGGTGGAAACTGGAAGACTAGAGTTCGGT AGGGGTAGCGGGAATTCCCAGTGTAGCGGTGA AATGCGTAGATATTGGGAAGAACATCGGTGGC GAAGGCGCGCTACTGGACCGAAACTGACACTC AGGGACGAAAGCTAGGGTAGCGAAAGGG
Bacteria	Proteobacteria	Gammaproteobacteria	Gammaproteobacteria	Gammaproteobacteria	Gammaproteobacteria	15	0	TACGGAGGGGGTTAGCGTTGTTCGGAATTACT GGGCGTAAAGCGCGCGTAGGCGGATTAGTAAG TTAGAGGTGAAATCCCGGGGCTCAACCTCGGA ACTGCATTTCGAACTGGCTGTCTAGAGTCTTGT AGAGGGTGGTGGAATTTCCAGTGTATCGGTGA AATGCGCAGAGATTGGAAGGAACATCAGTGGC GAAGGCGGCCACCTGGACAAAGACTGACGCTG AGGCACGAAAGCGTGGGGAGCGAACAGG
Bacteria	Proteobacteria	Gammaproteobacteria	Betaproteobacteriales	Burkholderiaceae	Massilia	15	0	TACGTAGGGTGCAAGCGTTAATCGGAATTACT GGGCGTAAAGCGTGCGCAGGCGGTTTTGTAAG TCTGACGTGAAAGCCCCGGGCTCAACCTGGGA ATTGCGTTGGAGACTGCAAGGCTTGAATCTGG CAGAGGGGGGTAGAATTCCACGTGTAGCAGTG AAATGCGTAGAGATGTGGAGGAACACCGATGG

Kingdom	Phylum	Class	Order	Family	Genus	Blank Total (n=1)	Sample Total (n=56)	Amplicon Sequence Variants (ASVs)
								CGAAGGCAGCCCCCTGGGTCAAGATTGACGCTCATGCACGAAAGCGTGGGGAGCAAACAGG
Bacteria	Proteobacteria	Gammaproteobacteria	Oceanospirillales	Litoricolaceae	Litoricola	15	0	TACGAAGGGGGCAAGCGTTACTCGGAATTATTGGGCGTAAAGCGTACGTAGGCGGTTGATTAAGCAGGATGTGAAAGCCCCGAGCTCAACTTGGGAACTGCATCCTGAACTGGTCAACTAGAGTACAGCAGAGGAGAGTGGAATTTCTCGTGTAGCGGTGAAATGCGTAGATATGAGAAGGAACACCAGTGGCGAAGGCGACTCTCTGGGTTGATACTGACGCTGAGGTACGAAAGCGTGGGGAGCAAACAGG
Bacteria	Proteobacteria	Gammaproteobacteria	Alteromonadales	Alteromonadaceae	Agaribacter	15	0	TACGGAGGGTGCGAGCGTTAATCGGAATTACTGGGCGTAAAGCGCACGCAGGCGGTCTGTTAAGCTAGAAGTGAAAGCCCTGCGCTCAACGTGGGAAGGCCTTTTAGAACTGGCAGACTAGAGTATTGGAGAGGGGAGTGGAATTCCACGTGTAGCGGTGAAATGCGTAGATATGTGGAGGAACATCGGTGGCGAAGGCGACTCTCTGGCCAAATACTGACGCTCATGTGCGAAAGTGTGGGTAGCGAACAGG
Bacteria	Proteobacteria	Alphaproteobacteria	Rhizobiales	Rhizobiales	Rhizobiales	15	0	TACGGAGGGGGCTAGCGTTGTTCGGAATTACTGGGCGTAAAGCGCGCGTAGGCGGGTCGGTCAGTCAGGGGTGAAAGCCCAGAGCTCAACTCTGGAACTGCCTTTGATACTGCCGACCTAGAGTGTGGGAGAGGTAAGCGGAATTCCGAGTGTAGAGGTGAAATTCGCAGATATTCGGAGGAACACCAGAGGCGAAGGCGGCTTACTGGACCATTACTGACGCTGAGGTGCGAAAGCGTGGGGAGCAAACAGG
Bacteria	Bacteroidetes	Bacteroidia	Flavobacteriales	Flavobacteriaceae	Flavobacteriaceae	15	0	TACGGAGGATGCAAGCGTTATCCGGAATCATTGGGTTTAAAGGGTCCGTAGGCGGGCTGGTAAGTCAGAGGTGAAAGTCTTGAGCTTAACTGGAGAATTGCCTTTGATACTGTTAGTCTTGAATCATTGTGAAGTAGTTAGAATGTGTAGTGTAGCGGTGAAATGCATAGATATTACACAGAATACCGATTGC

Kingdom	Phylum	Class	Order	Family	Genus	Blank Total (n=1)	Sample Total (n=56)	Amplicon Sequence Variants (ASVs)
								GAAGGCATGCTGCTAATGATGTATTGACGCTG ATGGACGAAAGCGTGGGTAGCGAACAGG
Bacteria	Proteobacteria	Deltaproteobacteria	NB1-j	NB1-j	NB1-j	15	0	TACGGAGGGTGCAAGCGTTGTTTGGAATCACT GGGCGTAAAGCGCGTGTAGGCGGCTTGGCAAG TCGGTTGTGAAATCCCGAGGCTCAACCTCGGA ACTGCAACCGAAACTGCCTCGCTAGAGTCCCG GAGAGGAGAGCGGAATTTCCGGTGTAGAGGTG AAATTCGTAGATATCGGAAGGAACACCAGTGG CGAAGGCGGCTCTCTGGACGGTGACTGACGCT GAGACGCGAAAGTGTGGGGAGCAAACAGG
Bacteria	Proteobacteria	Gammaproteobacteria	Vibrionales	Vibrionaceae	Vibriona ceae	15	0	TACGAAGGGGGCAAGCGTTACTCGGAATTATT GGGCGTAAAGCGTGCGTAGGCGGATAGTTAAG CGAGATGTGAAAGCCCGGGGCTCAACCTCGGA ACTGCATTTGAAACTGGCAAACTAGAGTACTG TAGAGGGGGGTAGAATTTCAGGTGTAGCGGTG AAATGCGTAGAGATCTGAAGGAATACCAGTGG CGAAGGCGGCCCCCTGGACAGATACTGACACT CAGATGCGAAAGCGTGGGGAGCAAACAGG
Bacteria	Proteobacteria	Gammaproteobacteria	Oceanospirillale s	Endozoicomon adaceae	Endozoi comonas	15	0	TACGGAGGGTGCAAGCGTTAATCGGAATTACT GGGCGTAAAGCGTGCGTAGGCGGCCTTTTAAG TTGGATGTGAAAGCCCCGGGCTCAACCTGGGA ACGGCATCCAAAACTGAGAGGCTCGAGTGCGG AAGAGGAGTGTGGAATTTCCTGTGTAGCGGTG AAATGCGTAGAGATTGGAAGGAACATCAGTGG CGAAGGCGGCCACCTGGACTGATACTGACGCT GAGGTGCGAAAGCGTGGGGAGCAAACAGG
Bacteria	Proteobacteria	Gammaproteobacteria	Betaproteobacter iales	Burkholderiace ae	Burkhol deriacea e	15	0	TACGTAGGGTGCGAGCGTTAATCGGAATTACT GGGCGTAAAGCGTGCGCAGGCGGTTGTGCAAG ACAGATGTGAAATCCCCGGGCTCAACCTGGGA ATTGCGCTTGTGACTGCATAGCTAGAGTATGTC AGAGGGGGGTAGAATTCCGCGTGTAGCAGTGA AATGCGTAGATATGCGGAGGAACACCGATGGC

Kingdom	Phylum	Class	Order	Family	Genus	Blank Total (n=1)	Sample Total (n=56)	Amplicon Sequence Variants (ASVs)
								GAAGGCAATCCCCTGGACCTGTACTGACGCTCATGCACGAAAGCGTGGGGAGCAAACAGG
Bacteria	Actinobacteria	Acidimicrobiia	Actinomarinales	Actinomarinaceae	Candidatus_Actinomarina	15	0	TACGGAGGGTGCGAGCGTTGTCCGGATTTATTGGGTTTAAAGGGTGCGTAGGCGGTTCAACAAGTCGGTCGTGAAAGTTCAGGGCTCAACCCTGAAATGTCGATCGATACTGTTGTGACTAGGATACGGTAGAGGTGAGTGGAATTCCGAGTGTAGCGGTGAAATGCGTAGATATTCGGAGGAACACCAATTGCGAAGGCAGCTCACTGGGCCGCTATCGACGCTGAGGAGCGAAAGCTAGGGGAGCAAACAGG
Bacteria	Proteobacteria	Alphaproteobacteria	Puniceispirillales	SAR116_clade	SAR116_clade	15	0	TACGAAGGGGGCGAGCGTTGTTCGGAATTACTGGGCGTAAAGGGAGCGCAGGCGGTTCATTTAGTTAGGCGTGAAAGCCCCGGGCTCAACCTGGGAACTGCGCTTAATACTGATGAACTAGAAAACAGAAGAGGATAGTGGAATTCCCAGTGTAGAGGTGAAATTCGTAGATATTGGGAAGAACACCGGTGGCGAAAGCGGCTATCTGGTCTGATTTTGACGCTGATGCTCGAAAGCGTGGGGAGCAAACAGG
Bacteria	Proteobacteria	Gammaproteobacteria	SAR86_clade	SAR86_clade	SAR86_clade	16	698	TACGGAAGGTCCAAGCGTTAATCGGAATTACTGGGCGTAAAGCGCGCGTAGGTGGTTTTTTAAGTTGGATGTGAAAGCCCTGGGCTCAACCTAGGAACTGCATCCAAAACTAGATGACTAGAGTACGAAAGAGGGAAGTAGAATTCACAGTGTAGCGGTGGAATGCGTAGATATTGTGAAGAATACCAATGGCGAAGGCAGCTTCCTGGTTCTGTACTGACACTGAGGTGCGAAAGCGTGGGTAGCGAACAGG
Bacteria	Proteobacteria	Proteobacteria	Proteobacteria	Proteobacteria	Proteobacteria	16	320	AACTTAAGGGCTAGTTTTGTGGGCAAAAGCGTAAAGGGTGTGATAGGCCGTTTTAATTAAAAAGGGTTTTATAATTTAAGAAGGTAAATGGAATTTTTTTGTAGCGGTGGAATGTGTAAATAGAAAAAAGAACTTTAGACGTGAAGACTATTTATTTTTGAGATTATAGTGTGATGGCTAAAACACGAGAGTATGGGGAGCAAACAGG

Kingdom	Phylum	Class	Order	Family	Genus	Blank Total (n=1)	Sample Total (n=56)	Amplicon Sequence Variants (ASVs)
Bacteria	Proteobacteria	Deltaproteobacteria	Desulfobacterales	Desulfobacteraceae	Sva0081_sediment_group	16	58	TACGGGGGGTGCAAGCGTTATTCGGAATTACTGGGCGTAAAGAGCGCGTAGGCGGTCTCTTAAGTCAGGTGTGAAAGCCCGGGCTTAACCCCGGAAGTGCACTTGAAACTAAGGGACTTGAGTATGGGAGAGGGAAGTGGAATTCCTGGTGTAGCGGTGAAATGCGTAGATATCAGGAGGAACATCGGTGGCGAAGGCGACTTCCTGGACCAATACTGACGCTGAGGCGCGAAGGCGTGGGGAGCAAACAGG
Bacteria	Proteobacteria	Gammaproteobacteria	Cellvibrionales	Halieaceae	OM60(NOR5)_clade	16	19	TACGGAGGGTGCAAGCGTTAATCGGAATTACTGGGCGTAAAGCGCGCGTAGGCGGTTTTTTAAGTCGGATGTGAAAGCCCCGGGCTCAACCTGGGAACTGCATCCGATACTGGATCACTAGAATGCGGGAGAGGGAGGTAGAATTCCATGTGTAGCAGTGAAATGCGTAGATATATGGAGGAATACCAGTGGCGAAGGCGGCCTCCTGGCTCGACATTGACGCTGAGGTGCGAAAGCGTGGGGAGCAAACGGG
Bacteria	Proteobacteria	Deltaproteobacteria	Desulfobacterales	Desulfobacteraceae	Sva0081_sediment_group	16	0	TACGGGGGGTGCAAGCGTTATTCGGAATTACTGGGCGTAAAGAGCGCGTAGGCGGTCTCTTAAGTCAGGTGTGAAAGCCCGGGCTCAACCCCGGAAGTGCACTTGAAACTATGGGACTTGAGTATGGGAGAGGGAAGTGGAATTCCTGGTGTAGCGGTGAAATGCGTAGATATCAGGAGGAACACCGGTGGCGAAGGCGACTTCCTGGACCAATACTGACGCTGAGGCGCGAAGGCGTGGGGAGCAAACAGG
Bacteria	Proteobacteria	Alphaproteobacteria	Puniceispirillales	SAR116_clade	SAR116_clade	16	0	TACGAAGGGGGCGAGCGTTATTCGGAATTATTGGGCGTAAAGGGCTCGCAGGCTGCTTGAACAGTTAGACGTGAAATCCCCGGGCTCAACCTGGGAACTGCGTTTAATACTAGCAAGCTAGAGTATTAGAGAGGATAGTGGAATTCCCAGTGTAGAGGTGAAATTCGTAGATATTGGGAAGAACACCAGTGGCGAAAGCGGCTCTCTGGTCCGATTTTGACGCTGAGGCGCGAAAGCGTGGGGAGCAAACAGG

Kingdom	Phylum	Class	Order	Family	Genus	Blank Total (n=1)	Sample Total (n=56)	Amplicon Sequence Variants (ASVs)
Bacteria	Proteobacteria	Alphaproteobacteria	SAR11_clade	Clade_IV	Clade_IV	16	0	TACGGAGGGGTCTAGCGTTATTCGGAATTACTGGGCGTAAAGCGAGCGTAGGCGGATTTGTAAGTTGGAGGTGAAATCCCAGAGCTTAACTCTGGAACTGCCTTCAAAACTACATTTCTTGAGTTTGGTAGAGGAGAGTGGAATTCCTAGTGTAGAGGTGAAATTCGTAGATATCAGGAGGAATATCGAAGGCGAAAGCATCTGTCTGGCTAAATACTGACGCTGTTGCACGAAAGCGTGGGGAGCAAACAGG
Bacteria	Proteobacteria	Gammaproteobacteria	SAR86_clade	SAR86_clade	SAR86_clade	16	0	TACGGAAGGTCCAAGCGTTAATCGGAATTACTGGGCGTAAAGCGCGCGTAGGTGGTTTATTAAGTTGGGTATGAAAGCCCCGGGCTCAACCTGGGAACTGCATCCAAAACTGATTCACTAGAGTACGAAAGAGGAAAGTAGAATTCACAGTGTAGCGGTGGAATGCGTAGATATTGTGAAGAATACCGATGGCGAAGGCAGCTTTCTGGTTCTGTACTGACACTGAGGTGCGAAAGCGTGGGTAGCGAACAGG
Bacteria	Bacteroidetes	Rhodothermia	Rhodothermales	Rhodothermaceae	Rhodothermaceae	16	0	TACGGAGGGTGCAAGCGTTGTCCGGATTCACTGGGTGTAAAGGGTGTGTAGGCGGGGCGGTAAGTCAGAGGTGAAAGCCCACGGCCCAACCGTGGAATTGCCTTTGATACTGCCGTTCTTGAGTCTCGGAGAGGTCGCTGGAATTCGTGGTGTAGCGGTGAAATGCGTAGATATCACGAGGAACACCAGAGGCGTAGGCGGGCGACTGGACGAGAACTGACGCTGAGGCACGAAAGCGTGGGGAGCAAACAGG
Bacteria	Proteobacteria	Gammaproteobacteria	Gammaproteobacteria	Gammaproteobacteria	Gammaproteobacteria	17	311	TACGGAGGGTCCAAGCGTTAATCGGAATTACTGGGCGTAAAGCGCGCGTAGGCGGCTTGTTAAGTCAGATGTGAAAGCCCCGGGCTCAACCTGGGAACTGCATTTGAAACTGGCAAGCTAGAGTGTGTGAGAGGTGAGTGGAATTTCCAGTGTAGCGGTGAAATGCGTAGAGATTGGAAGGAACATCAGTGGCGAAGGCGACTCACTGGCATAACACTGACGCTGAGGTGCGAAAGCGTGGGTAGCAAACAGG

Kingdom	Phylum	Class	Order	Family	Genus	Blank Total (n=1)	Sample Total (n=56)	Amplicon Sequence Variants (ASVs)
Bacteria	Proteobacteria	Gammaproteobacteria	Alteromonadales	Alteromonadaceae	Agaribacter	17	85	TACGGAGGGTGCGAGCGTTAATCGGAATTACTGGGCGTAAAGCGCACGCAGGCGGTCTGTTAAGCTAGAAGTGAAAGCCCCGGGCTCAACCTGGGAAGGCCTTTTAGAACTGGCAGACTAGAGTCTTGGAGAGGGGAGTGGAATTCCACGTGTAGCGGTGAAATGCGTAGATATGTGGAGGAACATCAGTGGCGAAGGCGACTCCCTGGCCAAAGACTGACGCTCATGTGCGAAAGTGTGGGTAGCGAACAGG
Bacteria	Bacteroidetes	Bacteroidia	Cytophagales	Flammeovirgaceae	Flexithrix	17	65	TACGGAGGGTGCAAGCGTTGTCCGGATTTATTGGGTTTAAAGGGTACGTAGGCGGTATATTAAGTCAGTGGTGAAAGTCTTCAGCTTAACTGAAGAAGTGCCATTGATACTGGTATACTTGAGTGTTGTAAGGGTGGGCGGAATTCCGCATGTAGCGGTGAAATGCATAGATATGCGGAGGAACACCAAAAGCGAAGGCAGCTCACTAGGCAACAACTGACGCTGAGGTACGAAAGCGTGGGGAGCGAACAGG
Bacteria	Bacteroidetes	Bacteroidia	Cytophagales	Cyclobacteriaceae	Imperialibacter	17	18	TACGGAGGGTGCAAGCGTTGTCCGGATTTATTGGGTTTAAAGGGTGCGTAGGCGGTCAATTAAGTCAGTGGTGAAAGGTAGCAGCTCAACTGTTAAATTGCCATTGATACTGGTTGACTGGAGTGCAGTTGAGGTAGGCGGAATTTATGGTGTAGCGGTGAAATGCATAGATACCATAAAGAACACCGATAGTGAAGACAGCTTACCAAGTTGTAACTGACGCTGAGGCACGAAAGCATGGGGAGCGAACAGG
Bacteria	Cyanobacteria	Oxyphotobacteria	Oxyphotobacteria	Oxyphotobacteria	Oxyphotobacteria	17	0	TACGGAGGGTGCAAGCGTTAATCGGAATTACTGGGCGTAAAGCGTCCGTAGGCGGCCAATCAAGTCTGCTGTTAAAGCGTGCAGCTTAACTGCATACCGGCAGTGGAAACTGATTGGCTTGAGTATGGTAGGGGTAGAGGGAATTCCCGGTGTAGCGGTGAAATGCGTAGATATCGGGAAGAACACCAGTGGCGAAGGCGCTCTACTGGGCCATAACTGACGCTGATGGACGAAAGCTAGGGGAGCGAAAGGG

Kingdom	Phylum	Class	Order	Family	Genus	Blank Total (n=1)	Sample Total (n=56)	Amplicon Sequence Variants (ASVs)
Bacteria	Proteobacteria	Proteobacteria	Proteobacteria	Proteobacteria	Proteobacteria	17	0	TATGGAGGGGGCGAGCGTTGTTCGGATTTACTGGGCGTAAAGGGTCCGTAGGTGGTTTTGTAAGCGAGATGTGAAAGCCCTGGGCTCAACCTAGGAACTGCATTTCGAACTGCATCACTAGAGTCATGGAGAGGATAGCGGAATTTCCAGTGTAGAGGTGAAATTCGTAGATATTGGAAGGAACACCGAAGGCGAAGGCAGCTATCTGGACATGTACTGACACTGAGGGACGAAAGCGTGGGGAGCAAACAGG
Bacteria	Proteobacteria	Gammaproteobacteria	Gammaproteobacteria	Gammaproteobacteria	Gammaproteobacteria	17	0	TACGGAGGGTGCGAGCGTTAATCGGAATTACTGGGCGTAAAGCGCGCGTAGGTGGCTTGGTCAGTCGGATGTGAAAGCCCCGGGCTTAACCTGGGAATTGCATTCGATACTGCCTGGCTAGAGTATGATAGAGGAAAGTGGAATTCCCGGTGTAGCGGTGAAATGCGTAGAGATCTGAAGGAATACCGGTGGCGAAGGCGGCCCCCTGGACAGATACTGACACTCAGATGCGAAAGCGTGGGGAGCAAACAGG
Bacteria	Proteobacteria	Alphaproteobacteria	Rhodobacterales	Rhodobacteraceae	Rhodobacteraceae	17	0	TACGGAGGGGGTTAGCGTTGTTCGGAATTACTGGGCGTAAAGCGCGCGTAGGCGGACCAGAAAGTTAGAGGTGAAATCCCAGGGCTCAACCCTGGAACTGCCTTTAAAACTCCTGGTCTTGAGTTCGAGAGAGGTGAGTGGAATTCCGAGTGTAGAGGTGAAATTCGTAGATATTCGGAGGAACACCAGTGGCGAAGGCGGCTCACTGGCTCGATACTGACGCTGAGGTGCGAAAGTGTGGGGAGCAAACAGG
Bacteria	Bacteroidetes	Bacteroidia	Cytophagales	Cyclobacteriaceae	Ekhidna	17	0	TACGGAGGGTGCAAGCGTTGTCCGGATTTATTGGGTTTAAAGGGTACGTAGGCGGATTTTTAAGTCAGTGGTGAAAGCCTGCAGCTTAACTGTAGAACTGCCATTGATACTGAAAATCTTGAGTTCAGTTGAGGTAAGCGGAATTTATGATGTAGCGGTGAAATGCATAGATATCATAAAGAACACCTATTGCGAAGGCAGCTTGCTAAGCTTGGACTGACGCTAAGGTACGAAAGCGTGGGTAGCGAACAGG

Kingdom	Phylum	Class	Order	Family	Genus	Blank Total (n=1)	Sample Total (n=56)	Amplicon Sequence Variants (ASVs)
Bacteria	Proteobacteria	Gammaproteobacteria	Thiohalorhabdales	Thiohalorhabdaceae	Thiohalorhabdaceae	18	428	TACGGAAGGTGCAAGCGTTAATCGGAATTACTGGGCGTAAAGCGCACGTAGGCGGTTGGGTTAGTCGGATGTGAAAGCCCCGGGCTTAACCTGGGAATTGCATTCGATACTGCCCGACTAGAGTATGGGAGAGGGTAGCGGAATTCCGGGTGTAGCGGTGAAATGCGTAGATATCCGGAGGAACATCAGTGGCGAAGGCGGCTGCCTGGCCAATACTGACGCTGAGGTGCGAAAGCGTGGGGAGCAAACAGG
Bacteria	Proteobacteria	Gammaproteobacteria	Gammaproteobacteria	Gammaproteobacteria	Gammaproteobacteria	18	22	TACGGAAGGTGCGAGCGTTAATCGGAATCACTGGGCGTAAAGCGCGCGTAGGCGGTTTGGTAAGTCAGATGTGAAAGCCCTGGGCTCAACCTGGGAACTGCATTTGATACTGCCTGACTAGAGTGTGGTAGAGGGAAGTGGAATTCCACATGTAGCGGTGAAATGCGTAGAGATGTGGAGGAACACCAATGGCGAAGGCAGCTTCCTGGACCAACACTGACGCTGAGGCGCGAAAGCGTGGGGAGCAAACAGG
Bacteria	Bacteroidetes	Bacteroidia	Cytophagales	Cyclobacteriaceae	Fulvivirga	18	18	TACGGAGGGTGCAAGCGTTGTCCGGATTTATTGGGTTTAAAGGGTGCGTAGGCGGTCGATTAAGTCAGTGGTGAAATCCTGCAGCTCAACTGTAGAACTGCCATTGATACTGGTTGACTTGAGTACAGACGAGGTAGGCGGAATTTATGGTGTAGCGGTGAAATGCATAGATACCATAAAGAACACCGATAGCGAAGGCAGCTTACTAGACTGTAACTGACGCTGAGGCACGAAAGCGTGGGGAGCGAACAGG
Bacteria	Proteobacteria	Alphaproteobacteria	Alphaproteobacteria	Alphaproteobacteria	Alphaproteobacteria	18	10	TACGGAGGGGGCAAGCGTTGCTCGGAATTACTGGGCGTAAAGCGAGCGTAGGCGGTTAATCAAGTTGGTGGTGAAAGCCCGGAGCTCAACTCCGGAACTGCCATCAAAACTGATTAACTTGAGATTGGTAGAGGAAAATGGAATTCCTAGTGTAGAGGTGAAATTCGTAGATATTGGGAAGAACATCGGTGGCGAAGGCGATTTTCTGGGCCATATCTGACGCTGAGGTTCGAAAGCGTGGGTAGCAAACAGG

Kingdom	Phylum	Class	Order	Family	Genus	Blank Total (n=1)	Sample Total (n=56)	Amplicon Sequence Variants (ASVs)
Bacteria	Bacteroidetes	Bacteroidia	Flavobacteriales	Flavobacteriales	Flavobacteriales	18	0	TACGGAGGGTGCAAGCGTTATCCGGATTCATTGGGTTTAAAGGGTCCGCAGGCTGTTAGATAAGTCAGAGGTGAAATCCCATCGCTCAACGATGGAACTGCCTTTGAAACTGTCTGACTAGAATGTATGTGAAGTAGGCGGAATGAGTAGTGTAGCGGTGAAATGCATAGATATTACTCAGAACACCGATTGCGAAGGCAGCTTACTAACATATTATTGACGCTGATGGACGAAAGCGTGGGTAGCGAACAGG
Bacteria	Proteobacteria	Gammaproteobacteria	Vibrionales	Vibrionaceae	Catenococcus	18	0	TACGGAGGGTGCAAGCGTTAATTGGAATTACTGGGCGTAAAGCGCGCGTAGGTGGTTTGTTAAGTCAGATGTGAAAGCCCGGGGCTCAACCTCGGAATTGCATTTGAAACTGGCAGACTAGAGTACTGTAGAGGGGGGTAGAATTTCAGGTGTAGCGGTGAAATGCGTAGAGATCTGAAGGAATACCGGTGGCGAAGGCGGCCCCCTGGACAGATACTGACACTCAGATGCGAAAGCGTGGGGAGCAAACAGG
Bacteria	Proteobacteria	Gammaproteobacteria	Alteromonadales	Pseudoalteromonadaceae	Algicola	18	0	TACGGAGGGTGCGAGCGTTAATCGGAATTACTGGGCGTAAAGCGTACGCAGGCGGTTAGTTAAGTCAGATGTGAAAGCCCCGGGCTCAACCTGGGAACTGCATTTGAAACTGGCTAACTAGAGTGCGGCAGAGGGGGGTAGAATTTCAGGTGTAGCGGTGAAATGCGTAGAGATCTGAAGGAATACCGGTGGCGAAGGCGGCCCCCTGGACAGATACTGACACTCAGATGCGAAAGCGTGGGTAGCAAACAGG
Bacteria	Proteobacteria	Alphaproteobacteria	Rhodobacterales	Rhodobacteraceae	Rhodobacteraceae	18	0	TACGGAGGGGGTTAGCGTTGTTCGGAATTACTGGGCGTAAAGCGCACGTAGGCGGATTAATTAGTCAGGGGTGAAATCCCGAGGCTCAACCTCGGAACTGCCTTTGATACTGTTAATCTTGAGTTCGAGAGAGGTGAGTGGAATTCCGAGTGTAGAGGTGAAATTCGTAGATATTCGGAGGAACACCAGTGGCGAAGGCGGCTCACTGGCTCGATACTGACGCTGAGGTGCGAAAGCGTGGGGAGCAAACAGG

Kingdom	Phylum	Class	Order	Family	Genus	Blank Total (n=1)	Sample Total (n=56)	Amplicon Sequence Variants (ASVs)
Bacteria	Proteobacteria	Gammaproteobacteria	Alteromonadales	Psychromonadaceae	Agarivorans	19	2232	TACGGAGGGTGCGAGCGTTAATCGGAATTACTGGGCGTAAAGCGTACGCAGGCGGCTAAGTCAGTCAGATGTGAAAGCCCGGGGCTTAACCTCGGAACGGCATTTGATACTGCTTAGCTAGAGTTTTGTAGAGGGTGGTAGAATTTCAGGTGTAGCGGTGAAATGCGTAGAGATCTGAAGGAATACCAGTGGCGAAGGCGGCCACCTGGACAAGAACTGACGCTCATGTACGAAAGCGTGGGGAGCAAACAGG
Bacteria	Proteobacteria	Gammaproteobacteria	Alteromonadales	Alteromonadaceae	Salinimonas	19	190	TACGGAGGGTGCAAGCGTTAATCGGAATTACTGGGCGTAAAGCGCACGCAGGCGGTTTGTTAAGCTAGATGTGAAAGCCCCGGGCTCAACCTGGGATGGTCATTTAGAACTGGCAGACTAGAGTTTTGGAGAGGGGAGTGGAATTCCAGGTGTAGCGGTGAAATGCGTAGATATCTGGAGGAACATCAGTGGCGAAGGCGACTCTCTGGCCAAAGACTGACGCTCATGTGCGAAAGTGTGGGTAGCGAACAGG
Bacteria	Bacteria	Bacteria	Bacteria	Bacteria	Bacteria	19	2	TACGTAAGTGGCGAACGTTGTCCGGAATCACTGGGCGTAAAGAGCACGTAGGCCGCCGGGTAAGTCGGAGGTGAAATCCAGGGGCTCAACCCCTGAACTGCCTCGGAAACTGCCTGGCTAGAGGTCCGGAGGGGAAAGTGGAATTCCGGGTGTAGCGGTGAAATGCGCAGATATCCGGGAGAACATCAATGGCGAAGGCAGCTTTCTGGCCGGATCCTGACGCTGAGGTGCGAAAGCCAGGGTAGCGAACGGG
Bacteria	Bacteria	Bacteria	Bacteria	Bacteria	Bacteria	19	0	TACGGGGGGTGCAAGCGTTATTCGGAATCACTGGGCATAAAGCGCGCGTAGGCGTTTTAGTAAGTCTCTTGTGAAATCCTATGGCTTAACCATAGCACTGCAAGGGAAACTACTAAAATAGAGTGCAGAAGAGGTAGCTGGAATTTCTAGTGTAGGGGTAAAATCCGTTAATATTAGAAGGAATACCGATGGCGAAAGCAAGCTACTGGGATGTTACTGACGCTAAGGTGCGAAAGCGTGGGGAGCAAACAGG

Kingdom	Phylum	Class	Order	Family	Genus	Blank Total (n=1)	Sample Total (n=56)	Amplicon Sequence Variants (ASVs)
Bacteria	Bacteroidetes	Bacteroidia	Flavobacteriales	Flavobacteriales	Flavobacteriales	19	0	TACGGAGGGTGCAAGCGTTATCCGGATTTATTGGGTTTAAAGGGTGCGCAGGCGGGATGCTAAGTCAGTGGTGAAATCCTTCAGCTCAACTGGAGAACTGCCATTGATACTGGTATCCTTGAGTATAGATGAAGTGGGCGGAATGTGTCATGTAGCGGTGAAATGCATAGATATGACACAGAACACCGATTGCGAAGGCAGCTCACTAAACTATAACTGACGCTGAGGCACGAAAGCGTGGGGAGCGAACAGG
Bacteria	Lentisphaerae	Lentisphaeria	Lentisphaerales	Lentisphaeraceae	Lentisphaera	19	0	TACGTGTGGGGCAAGCGTTGTTCGGAATCACTGGGCATAAAGGGTACGTAGGGGGATAAATAAGTTTGATGTGAAATTTCGAGGCTCAACCTCGAACCTGCATTGAAAACTGTTTATCTAGAATTCGGTAGAGGTAAGTGGAATTTGTGGTGTAGCGGTGGAATGCGTAGATATCACAAGGAACATCAAAGGCGAAAGCAGCTTACTGGGCCGATATTGACCCTGAGGGACGAAAGCTAAGGTAGCGAAAAGG
Bacteria	Cyanobacteria	Oxyphotobacteria	Nostocales	Oscillatoriaceae	Planktothricoides_SR001	19	0	TACGGAGGAGGCAAGCGTTATCCGGAATTATTGGGCGTAAAGGGTCCGCAGGTGGTAGTGCAAGTCGGCGGTTAAAGACTGGGGCTTAACTCCAGAAAGGCCGTGGAAACTGCACAACTAGAGAGCGGTAGGGGTAGAGGGAATTCCCGGTGTAGCGGTGAAATGCGTAGAGATCGGGAAGAACACCAGTGGCGAAGGCGCTCTACTGGACCGCAACTGACACTGAGGGACGAAAGCTAGGGGAGCGAATGGG
Bacteria	Proteobacteria	Gammaproteobacteria	Vibrionales	Vibrionaceae	Catenococcus	19	0	TACGGAGGGTGCAAGCGTTAATCGGAATTACTGGGCGTAAAGCGCATGCAGGTGGTTTGTTAAGTCAGATGTGAAAGCCCTGGGGCTCAACCTCGGAATAGCATTTGAAACTGGCAGACTAGAGTACTGTAGAGGGGGGTAGAATTTCAGGTGTAGCGGTGAAATGCGTAGAGATCTGAAGGAATACCGGTGGCGAAGGCGGCCCCCTGGACAGATACTGACACTCAGATGCGAAAGCGTGGGGAGCAAACAGG

Kingdom	Phylum	Class	Order	Family	Genus	Blank Total (n=1)	Sample Total (n=56)	Amplicon Sequence Variants (ASVs)
Bacteria	Bacteroidetes	Bacteroidia	Flavobacteriales	Cryomorphaceae	Cryomorphaceae	20	840	TACGGAGGATCCAAGCGTTATCCGGATTCATTGGGTTTAAAGGGTCCGTAGGCGGATTTTTAAGTCAGTGGTGAAAGCCGACAGCTCAACTGTCGAACTGCCATTGATACTGGAAATCTTGAGTACAAATGAAGTAGGCGGAATGAGTCATGTAGCGGTGAAATGCATAGATATGACTCAGAACACCGATTGCGAAGGCAGCTTACTAACATGTAACTGACGCTGAGGGACGAAAGCGTGGGGAGCGAACAGG
Bacteria	Proteobacteria	Alphaproteobacteria	Rhodobacterales	Rhodobacteraceae	Rhodobacteraceae	20	229	TACGGAGGGGGTTAGCGTTGTTCGGAATTACTGGGCGTAAAGCGCGCGTAGGCGGACTATTAAGTGAGGGGTGAAATCCCGGGGCTCAACCCCGGAACTGCCTTTCATACTGGTAGTCTTGAGTTCGAGAGAGGTGAGTGGAATTCCGAGTGTAGAGGTGAAATTCGTAGATATTCGGAGGAACACCAGTGGCGAAGGCGGCTCACTGGCTCGATACTGACGCTGAGGTGCGAAAGTGTGGGGAGCAAACAGG
Bacteria	Proteobacteria	Alphaproteobacteria	Puniceispirillales	SAR116_clade	SAR116_clade	20	0	TACGAAGGGGGCGAGCGTTGTTCGGAATTATTGGGCGTAAAGGGCGCGCAGGCGGTTTGGATAGTTAGACGTGAAAGCCCTGGGCTTAACCCAGGAACTGCGTTTAATACTGCCAAACTAGAGAACTAGAGAGGATAGCGGAACTCCCAGTGTAGAGGTGAAATTCGTAGATATTGGGAAGAACATCAGTGGCGAAAGCGGCTGTCTGGCTAGTTTCTGACGCTGAGGCGCGAAAGCGTGGGGAGCAAACAGG
Bacteria	Bacteroidetes	Bacteroidia	Cytophagales	Flammeovirgaceae	Flexithrix	20	0	TACGGAGGGTGCGAGCGTTGTCCGGATTTATTGGGTTTAAAGGGTACGTAGGCGGTATATTAAGTCAGTGGTGAAATCCTGCAGCTCAACTGTGGACGTGCCATTGATACTGATATACTTGAGTGTTGTAAGGGTGGGCGGAATTCCGCATGTAGCGGTGAAATGCATAGATATGCGGAGGAACACCGAAAGCGAAGGCAGCCCACTAGGCAACAACTGACGCTGAGGTACGAAAGCGTGGGGAGCGAACAGG

Kingdom	Phylum	Class	Order	Family	Genus	Blank Total (n=1)	Sample Total (n=56)	Amplicon Sequence Variants (ASVs)
Bacteria	Proteobacteria	Gammaproteobacteria	Alteromonadales	Pseudoalteromonadaceae	Pseudoalteromonas	20	0	TACGGAGGGTGCGAGCGTTAATCGGAATTACTGGGCGTAAAGCGTACGCAGGCGGTTTGTTAAGCGAGATGTGAAAGCCCCGGGCTCAACCTGGGAACTGCATTTCGAACTGGCAAACTAGAGTGTGATAGAGGGTGGTAGAATTTCAGGTGTAGCGGTGAAATGCGTAGAGATCTGAAGGAATACCGATGGCGAAGGCAGCCACCTGGGTCAACACTGACGCTCATGTACGAGAGTGTGGGGAGCAAACAGG
Bacteria	Proteobacteria	Gammaproteobacteria	Steroidobacterales	Woeseiaceae	Woeseia	20	0	TACGGAGGGTGCGAGCGTTAATCGGAATTACTGTGCGTAAAGCGCGCGTAGGCGGTTTTGTAAGTCGGATGTGAAATCCCCGGGCTTAACCTGGGAACTGCATTCGATACTGCATAACTAGAGTATGGTAGAGGGAAGTGGAATTTCCGGTGTAGCGGTGAAATGCGTAGATATCGGAAGGAACACCAGTGGCGAAGGCGACTTCCTGGGCCAATACTGACGCTGAGGTGCGAAAGCGTGGGGAGCAAACAGG
Bacteria	Proteobacteria	Alphaproteobacteria	SAR11_clade	Clade_I	Clade_Ia	21	4901	TACGAAGGGACCTAGCGTAGTTCGGAATTACTGGGCTTAAAGAGTTCGTAGGTGGTTGAAAAAGTTGGTGGTGAAATCCCAGAGCTTAACTCTGGAACTGCCATCAAAACTTTTCAGCTAGAGTTTGATAGAGGAAAGCAGAATTTCTAGTGTAGAGGTGAAATTCGTAGATATTAGAAAGAATACCAATTGCGAAGGCAGCTTTCTGGATCATTACTGACACTGAGGAACGAAAGCATGGGTAGCGAAGAGG
Bacteria	Bacteroidetes	Bacteroidia	Cytophagales	Amoebophilaceae	Candidatus_Amoebophilus	21	40	TACGGAGGGTGCAAGCGTTATCCGGATTTACTGGGTTTAAAGAGTGCGTAGGCGGCTTTTTAAGTCAGTGGTGAAAGCCTTGGCGCTTAACGCTAGAAGTGCCACTGATACTAGAAAGCTTGAGTCAAGAAGAGGTAAGCAGAATTCATAGTGTAGCAGTGAAATGCTTAGATACTATGAGGAATACCAACAGCGAAAGCAGCTTACTGGTCTTGTACTGACGCTGAGGCACGAAAGCGTGGGTAGCGAACAGG

Kingdom	Phylum	Class	Order	Family	Genus	Blank Total (n=1)	Sample Total (n=56)	Amplicon Sequence Variants (ASVs)
Bacteria	Proteobacteria	Alphaproteobacteria	Rickettsiales	Rickettsiaceae	Rickettsiaceae	21	19	TACGGAGGGTGCTAGCGTTGTTCGGATTTACTGGGCGTAAAGGGCACGTAGGCTGTCTATCAAGTTGGGAGTGAAATCCCGGGGCTCAACCTCGGAACTGCTCTCAAAACTGATAGACTAGAGTTAAGTAGAGGATAGTGGAATTCCCAGTGTAGAGGTGAAATTCGTAGATATTGGGAGGAACACCAGAAGCGAAGGCGACTATCTGGGCTTATACTGACGCTGAGGTGCGAAAGTGTGGGGAGCAAACAGG
Bacteria	Chlamydiae	Chlamydiae	Chlamydiales	Simkaniaceae	Simkaniaceae	21	0	TACGGAGGGTGCAAGCGTTAATCGGATTTATTGGGCGTAAAGAGCGCGTAGGCGGGAACTTAAGTCAGATGTGAAATTCCAGAGCTCAACTTTGGAGCTGCATTTGAAACTGAGTTTCTTGAGGACGGACGGAGAAAACGGAATTCCGAGTGTAGCGGTGAAATGCGTAGATATTCGGAAGAACACCCGTGGCGAAAGCGGTTTTCTAGTTTGTGCCTGACGCTGAGGCGCGAAAGCAAGGGGAGCGAACAGG
Bacteria	Bacteroidetes	Bacteroidia	Chitinophagales	Saprospiraceae	Saprospira	21	0	TACGGAGGGTGCGAACGTTATTCGGATTTACTGGGCGTAAAGCGTCTGTAGGCGGTTCAGTAAGTCAGATGTGAAATGTCGGAGCTCAACTTCGAACTTGCATTTGAAACTGTTGAACTTGAATTAGGTGGAAGTGTGCGGAATGTATCATGTAGCGGTGAAATGCATAGATATGATATAGAACACCAATAGCGAAGGCAGCACACTACGCTTTGATTGACGCTGAGGTACGAAAGCGTGGGGAGCGAACAGG
Bacteria	Planctomycetes	vadinHA49	vadinHA49	vadinHA49	vadinHA49	21	0	TACGTGTGGAGCAAGCGTTGTCCGGAATCACTGGGCATAAAGGGTACGTAGGCGGCCTTGTAAGTCAGTGGTGAAATACCCCGGCCCAACCGGTGGAATTGCCATTGATACTGCTTGGCTTGAGTGTGTGAGGGGTGAGCGGAACGTTTGGTGTAGCGGTGAAATGCGTAGATATCAAACGGAAGGCCGGAGGCGAAGGCGGCTCACTGGCACACAACTGACGCTGAGGTACGAAAGCGTGGGGAGCAAACAGG

Kingdom	Phylum	Class	Order	Family	Genus	Blank Total (n=1)	Sample Total (n=56)	Amplicon Sequence Variants (ASVs)
Bacteria	Proteobacteria	Alphaproteobacteria	Puniceispirillales	SAR116_clade	SAR116_clade	21	0	TACGAAGGGGGCGAGCGTTATTCGGAATTATTGGGCGTAAAGGGCTCGCAGGCTGCTTGAACAGTTAGACGTGAAATCCCCGGGCTCAACCTGGGAACTGCGTTTAATACTAGCAAGCTAGAGAAATAGAGAGGAAAGTGGAACTCCCAGTGTAGAGGTGAAATTCGTAGATATTGGGAAGAACACCAGTGGCGAAAGCGACTTTCTGGCTATTTTCTGACGCTCAGGAGCGAAAGCGTGGGGAGCAAACAGG
Bacteria	Proteobacteria	Alphaproteobacteria	Rhodospirillales	AEGEAN-169_marine_group	AEGEAN-169_marine_group	22	448	TACGAAGGGGGCTAGCGTTGTTCGGAATCACTGGGCGTAAAGCGTATGTAGGCGGATCAAAAAGTTAGATGTGAAATCCCTGGGCTCAACCCAGGAACTGCATTTAAAACTAGTGATCTAGAATTTGGTAAAGGTTAGTAGAATTTCCAGTGTAGAGGTGAAATTCGTAGATATTGGAAAGAATACCAGAAGCGAAGGCGACTAACTAGGCCATTTTTGACGCTGAGATACGAAAGCGTGGGTAGCAAACAGG
Bacteria	Proteobacteria	Gammaproteobacteria	Alteromonadales	Alteromonadaceae	Agaribacter	22	89	TACGGAGGGTGCGAGCGTTAATCGGAATTACTGGGCGTAAAGCGCACGCAGGCGGTTAATTAAGCTAGAAGTGAAAGCCCTGCGCTCAACGTGGGAAGGCCTTTTAGAACTGGTTAACTAGAGTATTGGAGAGGGGAGTGGAATTCCAGGTGTAGCGGTGAAATGCGTAGATATCTGGAGGAACACCGGTGGCGAAGGCGACTCTCTGGCCACATACTGACGCTCATGTGCGAAAGTGTGGGTAGCGAACAGG
Bacteria	Bacteroidetes	Bacteroidia	Cytophagales	Cyclobacteriaceae	Algoriphagus	22	3	TACGGAGGGTGCAAGCGTTGTCCGGATTTATTGGGTTTAAAGGGTGCGTAGGCGGCTAATTAAGTCAGCGGTGAAAGTTTTGGGCTCAACCCAGAAATTGCCATTGATACTGATTAGCTTGAGTATTGGAGGGGTACATGGAATTGATGGTGTAGCGGTGAAATGCATAGATACCATCAGGAACACCGATAGCGAAGGCATTGTACTGGCCAATAACTGACGCTGATGCACGAAAGCATGGGTAGCGAACAGG

Kingdom	Phylum	Class	Order	Family	Genus	Blank Total (n=1)	Sample Total (n=56)	Amplicon Sequence Variants (ASVs)
Bacteria	Proteobacteria	Gammaproteobacteria	Xanthomonadales	Xanthomonadaceae	Thermomonas	22	0	TACGAAGGGTGCAAGCGTTACTCGGAATTACTGGGCGTAAAGCGTGCGTAGGTGGTTCGTTAAGTCTGATGTGAAAGCCCTGGGCTCAACCTGGGAATTGCATTGGATACTGGCGGGCTAGAGTGCGGTAGAGGGTAGTGGAATTCCCGGTGTAGCAGTGAAATGCGTAGAGATCGGGAGGAACATCTGTGGCGAAGGCGACTGCCTGGACCAGCACTGACACTGAGGCACGAAAGCGTGGGGAGCAAACAGG
Bacteria	Proteobacteria	Alphaproteobacteria	Rhizobiales	Rhizobiales	Rhizobiales	22	0	TACGGAGGGGGTTAGCGTTGTTCGGAATTACTGGGCGTAAAGCGCACGTAGGCGGATTAATAAGTCAGGGGTGAAATCCCGAGGCTCAACCTCGGAACTGCCTTTGATACTGTTAATCTTGAGTTCGAGAGAGGTGAGTGGAATTCCATGTGTAGCAGTGAAATGCGTAGATATATGGAGGAATACCAGTGGCGAAGGCGGCCTCCTGGCTCGACATTGACGCTGAGGTGCGAAAGCGTGGGGAGCAAACGGG
Bacteria	Proteobacteria	Alphaproteobacteria	SAR11_clade	Clade_I	Clade_Ib	22	0	TACGAAGGGACCTAGCGTAGTTCGGAATTACTGGGCTTAAAGAGTTCGTAGGTTGTTAAAATAGTTGGTGGTGAAATCCCAGAGCTTAACTCTGGAACTGCCATCAAAACTTTTTAGCTAGAGTATGATAGAGGAAAGCAGAATTTCTAGTGTAGAGGTGAAATTCGTAGATATTAGAAAGAATACCAATTGCGAAGGCAGCTTTCTGGATCATTACTGACACTGAGGAACGAAAGCATGGGTAGCGAAGAGG
Bacteria	Proteobacteria	Gammaproteobacteria	Cellvibrionales	BD2-7	BD2-7	22	0	TACGGAGGGTGCAAGCGTTAATCGGAATTACTGGGCGTAAAGCGCGCGTAGGCGGTTTGTTAAGTCGGATGTGAAAGCCCCGGGCTCAACCTGGGAATTGCATTCGATACTGGCAGACTGGAATGCGGGAGAGGGAGGTAGAATTCCATGTGTAGCGGTGAAATGCGTAGATATATGGAGGAATACCAGTGGCGAAGGCGGCCTCCTGGCCTGACATTGACGCTGAGGTGCGAAAGCGTGGGGAGCAAACAGG

Kingdom	Phylum	Class	Order	Family	Genus	Blank Total (n=1)	Sample Total (n=56)	Amplicon Sequence Variants (ASVs)
Bacteria	Verrucomicrobia	Verrucomicrobiae	Opitutales	Puniceicoccaceae	Coraliomargarita	23	721	TACGGAGACTGCAAGCGTTACTCGGATTCACTGGGCGTAAAGGGTGCGTAGGCCGCTAAGTGTGTCAGGTGTGAAATCTCGGGGCTCAACCTCGAAACTGCGCCTGAAACTACTTAGCTTGAGTTTCGGAGAGGTAAGCGGAATTTCTGGTGTAGCGGTGAAATGCGTAGATATCAGAAGGAACACCAATGGCGAAGGCAGCTTACTGGACGACAACTGACGCTGAGGCACGAAAGCGTGGGTAGCGAAAGGG
Bacteria	Proteobacteria	Alphaproteobacteria	Rhodobacterales	Rhodobacteraceae	Ascidiaceihabitans	23	416	TACGGAGGGGGTTAGCGTTGTTCGGAATTACTGGGCGTAAAGCGCGCGTAGGCGGATTGGAAAGTTGGGGGTGAAATCCCGGGGCTCAACCCCGGAACTGCCTCCAAAACTATCAGTCTAGAGTTCGAGAGAGGTGAGTGGAATTCCAAGTGTAGAGGTGAAATTCGTAGATATTTGGAGGAACACCAGTGGCGAAGGCGGCTCACTGGCTCGATACTGACGCTGAGGTGCGAAAGTGTGGGGAGCAAACAGG
Bacteria	Proteobacteria	Gammaproteobacteria	Alteromonadales	Alteromonadaceae	Alteromonadaceae	23	170	TACGGAGGGTGCGAGCGTTAATCGGAATTACTGGGCGTAAAGCGCACGCAGGCGGTTACATAAGTCAGATGTGAAAGCCCCGGGCTTAACCTGGGAATAGCATTTGAAACTGTGTAACTAGAGTCTCGGAGAGGGGAGTGGAATTCCAGGTGTAGCGGTGAAATGCGTAGAGATCTGGAGGAACATCAATGGCGAAGGCAGCTCTCTGGCCGAAGACTGACGCTCATGTGCGAAAGTGTGGGTAGCGAACGGG
Bacteria	Actinobacteria	Actinobacteria	Corynebacteriales	Corynebacteriaceae	Corynebacterium	24	6	TACGTAGGGTGCGAGCGTTGTCCGGAATTACTGGGCGTAAAGAGCTCGTAGGTGGTTTGTCGCGTCGTCTGTGAAATTCCGGGGCTTAACTTCGGGCGTGCAGGCGATACGGGCATAACTTGAGTGCTGTAGGGGAGACTGGAATTCCTGGTGTAGCGGTGGAATGCGCAGATATCAGGAGGAACACCGATGGCGAAGGCAGGTCTCTGGGCAGTAACTGACGCTGAGGAGCGAAAGCATGGGGAGCGAACAGG

Kingdom	Phylum	Class	Order	Family	Genus	Blank Total (n=1)	Sample Total (n=56)	Amplicon Sequence Variants (ASVs)
Bacteria	Verrucomicrobia	Verrucomicrobiae	Verrucomicrobiales	DEV007	DEV007	24	0	TACGAAGGTCCCGAGCGTTGTTCGGATTTACTGGGCGTAAAGCGTCTGTAGGCCGCATGGTAAGTCAGGTGTGAAATCCCGGGCTCAACCCCGGAACTGCATCCGATACTGCCGTGCTAGAGGGTCGGAGGGGAGTCTGGAATTCTCGGTGTAGCAGTGAAATGCGTAGATATCGAGAGGAACACTAGTGGCGAAGGCGAGACTCTGGACGACACCTGACGCTGAGAGACGAAGGCTAGGGTAGCGAAAGG
Bacteria	Kiritimatiellaeota	Kiritimatiellae	WCHB1-41	WCHB1-41	WCHB1-41	24	0	TACAGAGGTGGCAAGCGTTGTTCGGATTTACTGGGCGTAAAGGGTGCGTAGGCGGTTCTGTGTGTCGGATGTGAAATCTCACCGTCCAACGGTGAACGTGCATTCGAAACTGCAGGACTCGAGTACAGGAGAGGAAAGTGGAATTCTCGGTGTAGCGGTGAAATGCGTAGATATCGAGAAGAACGCCGACGGCGAAGGCAACTTTCTAGACTGATACTGACGCTGAGGCACGAAAGCTGGGGTAGCGAAGAGG
Bacteria	Cyanobacteria	Oxyphotobacteria	Synechococcales	Cyanobiaceae	Prochlorococcus_MIT9313	24	0	TACGGGAGTGGCAAGCGTTATCCGGAATTATTGGGCGTAAAGCGTCCGCCGGCGGCTTTTCAAGTCTGCTGTTAAAGCGTGGAGCTTAACTCCATCATGGCAGTGGAAACTGAAAGGCTTGAGTATGGTAGGGGCAGAGGGAATTCCCGGTGTAGCGGTGAAATGCGTAGATATCGGGAAGAACACCAGTGGCGAAGGCGCTCTGCTGGGCCATTACTGACGCTCATGGACGAAAGCCAGGGGAGCGAAAGGG
Bacteria	Proteobacteria	Gammaproteobacteria	Oceanospirillales	Saccharospirillaceae	Thalassolituus	24	0	TACGGAGGGTGCAAGCGTTACTCGGAATCACTGGGCGTAAAGAGCGTGTAGGTGGTTTGTTAAGCGGAATGTGAAAGCCCCGGGCTCAACCTGGGAACTGCATTGCGAACTGGCAGACTAGAGTACAGTAGAGGGTAGTGGAATTTCCTGTGTAGCGGTGAAATGCGCAGAGATGGGAAGGAACATCAGTGGCGAAGGCGACTATCTGGCATATTATTGACGCTGAGGCGCGAAAGTGTGGGGAGCAAACAGG

Kingdom	Phylum	Class	Order	Family	Genus	Blank Total (n=1)	Sample Total (n=56)	Amplicon Sequence Variants (ASVs)
Bacteria	Proteobacteria	Alphaproteobacteria	Rhodobacterales	Rhodobacteraceae	Rhodobacteraceae	25	1384	TACGGAGGGGGTTAGCGTTGTTCGGAATTACTGGGCGTAAAGCGCACGTAGGCGGATTAATAAGTCAGGGGTGAAATCCCGAGGCTCAACCTCGGAACTGCCTTTGATACTGTTAATCTTGAGTTCGAGAGAGGTGAGTGGAATTCCGAGTGTAGAGGTGAAATTCGTAGATATTCGGAGGAACACCAGTGGCGAAGGCGGCTCACTGGCTCGATACTGACGCTGAGGTGCGAAAGCGTGGGGAGCAAACAGG
Bacteria	Proteobacteria	Alphaproteobacteria	Thalassobaculales	Thalassobaculales	Thalassobaculales	25	241	TACGGAGGGGGCAAGCGTTGTTCGGAATTACTGGGCGTAAAGGGCGCGTAGGCGGCCGATTGCGTCAGGTGTGAAATCCCCGGGCTCAACCTGGGAACTGCACTTGATACGGATTGGCTCGAGTATGTCAGAGGATGGTGGAATTCCCAGTGTAGAGGTGAAATTCGTAGATATTGGGAAGAACACCGATGGCGAAGGCAGCCATCTGGGGCATTACTGACGCTGAGGCGCGAAAGCGTGGGGAGCAAACAGG
Bacteria	Proteobacteria	Alphaproteobacteria	Caulobacterales	Hyphomonadaceae	Hyphomonadaceae	25	57	TACGAAGGGGGCTAGCGTTGTTCGGAATTACTGGGCGTAAAGCGCGCGTAGGCGGATTTTTAAGTCAGAGGTGAAATCCCGGTGCTCAACATCGGAACTGCCTTTGAAACTGGAAATCTAGAGGCCAGGAGAGGTCAGTGGAATACCGAGTGTAGAGGTGAAATTCGTAGATATTCGGTGGAACACCAGTGGCGAAGGCGACTGACTGGACTGGTACTGACGCTGAGGTGCGAAAGTGTGGGGAGCAAACAGG
Bacteria	Bacteroidetes	Bacteroidia	Flavobacteriales	Flavobacteriaceae	Aquibacter	25	17	TACGGAGGATGCAAGCGTTATCCGGAATCATTGGGTTTAAAGGGTCCGTAGGTGGACAGATAAGTCAGAGGTGAAATCCTGCAGCTCAACTGTAGAATTGCCTTTGATACTGTTTGTCTTGAGTCATTATGAAGTGGTTGGAATGAGTAGTGTAGCGGTGAAATGCATAGATATTACTCAGAACACCGATTGCGAAGGCAGATCACTAATAATGTACTGACACTGATGGACGAAAGCGTGGGGAGCGAACAGG

Kingdom	Phylum	Class	Order	Family	Genus	Blank Total (n=1)	Sample Total (n=56)	Amplicon Sequence Variants (ASVs)
Bacteria	Bacteroidetes	Bacteroidia	Chitinophagales	Saprospiraceae	Saprospira	25	0	TACGGAGGGTGCGAGCGTTAATCGGAATTACTGGGCGTAAAGCGTACGCAGGCGGCCATTTAAGTCAGATGTGAAATGTCGGAGCTCAACTTCGAACTTGCATTTGAAACTGTTGAACTTGAATTAGGTGGAAGTGTGCGGAATGTATCATGTAGCGGTGAAATGCATAGATATGATATAGAACACCAATAGCGAAGGCAGCACACTACGCTTTGATTGACGCTGAGGTACGAAAGCGTGGGGAGCGAACAGG
Bacteria	Bacteroidetes	Bacteroidia	Chitinophagales	Saprospiraceae	Lewinella	25	0	TACGGAGGGTGCAAGCGTTATCCGGAATCACTGGGTTTAAAGGGTGCGTAGGCGGACCATTAAGTCAGAGGTGAAATCCCGCAGCTTAACTGCGGAACTGCCTTTGATACTGATGGTCTTGAATTAGGTTGAGGTTAGCGGAATGTGGCATGTAGCGGTGAAATGCTTAGATATGCCATAGAACACCGATTGCGAAGGCAGCTAACTGGCCCTTGATTGACGCTGAGGCACGAAAGCGTGGGGAGCGAACAGG
Bacteria	Proteobacteria	Gammaproteobacteria	Alteromonadales	Alteromonadaceae	Agaribacter	25	0	TACGGAGGGTGCGAGCGTTAATCGGAATTACTGGGCGTAAAGCGCACGCAGGCGGTCTGTTAAGCTAGAAGTGAAAGCCCCGGGCTCAACCTGGGAAGGCCTTTTAGAACTGGCAGACTAGAGTCTTGGAGAGGGGAGTGGAATTCCACGTGTAGCGGTGAAATGCGTAGATATGTGGAGGAACATCAGTGGCGAAGGCGACTCCCTGGACAAAGACTGACGCTCAGATGCGAAAGCGTGGGTAGCAAACAGG
Bacteria	Proteobacteria	Gammaproteobacteria	Cellvibrionales	Cellvibrionaceae	Candidatus_Endobugula	25	0	TACGGAGGGTGCGAGCGTTAATCGGAATTACTGGGCGTAAAGCGCGCGTAGGCGGTTTGTTAAGCGGGATGTGAAATCCCCGGGCTCAACCTGGGAACTGCATTCCGAACTGGCAGGCTAGAGTACAAGAGAGGGTGGTGGAATTTCCAGTGTAGCGGTGAAATGCGTAGAGATTGGAAGGAACATCAGTGGCGAAGGCGGCCACCTGGATTGATACTGACGCTGAGGCGCGAAAGCGTGGGGAGCAAACAGG

Kingdom	Phylum	Class	Order	Family	Genus	Blank Total (n=1)	Sample Total (n=56)	Amplicon Sequence Variants (ASVs)
Bacteria	Actinobacteria	Actinobacteria	Micrococcales	Micrococcaceae	Micrococcus	26	164	TACGTAGGGTGCGAGCGTTATCCGGAATTATTGGGCGTAAAGAGCTCGTAGGCGGTTTGTCGCGTCTGTCGTGAAAGTCCGGGGCTTAACCCCGGATCTGCGGTGGGTACGGGCAGACTAGAGTGCAGTAGGGGAGACTGGAATTCCTGGTGTAGCGGTGGAATGCGCAGATATCAGGAGGAACACCGATGGCGAAGGCAGGTCTCTGGGCTGTAACTGACGCTGAGGAGCGAAAGCATGGGGAGCGAACAGG
Bacteria	Proteobacteria	Alphaproteobacteria	Rhodospirillales	AEGEAN-169_marine_group	AEGEAN-169_marine_group	26	0	TACGAAGGGGGCAAGCGTTACTCGGAATTATTGGGCGTAAAGCGTATGTAGGCGGATCAAAAAGTTAGATGTGAAATCCCTGGGCTTAACCCAGGAACTGCATTTAAAACTAGTGATCTAGAATTTGGTAAAGGTTAGTAGAATTTCCAGTGTAGAGGTGAAATTCGTAGATATTGGAAAGAATACCAGAAGCGAAGGCGACTAACTAGGCCATTTTTGACGCTGAGATACGAAAGCGTGGGTAGCAAACAGG
Bacteria	Proteobacteria	Gammaproteobacteria	Chromatiales	Sedimenticolaceae	Sedimenticolaceae	26	0	TACGGAGGGTGCGAGCGTTAATCGGAATTACTGGGCGTAAAGCGCACGTAGGCGGTTTGGTAAGTCAGATGTGAAAGCCCCGGGCTTAACCTGGGAACTGCATTTGATACTGCTGAACTAGAGTATGGTAGAGGCAAGTGGAATTCCAGGTGTAGCGGTGAAATGCGTAGATATCTGGAGGAACATCAGTGGCGAAGGCGACTTGCTGGACCAATACTGACGCTGAGGTGCGAAAGCGTGGGGAGCAAACAGG
Bacteria	Proteobacteria	Alphaproteobacteria	Rickettsiales	Midichloriaceae	MD3-55	26	0	TACGAAGGGGGCAAGCGTTACTCGGAATTATTGGGCGTAAAGCGTGCGTAGGCGGTTTTATAAGTTGAAAGTGAAAGCCTTTGGCTCAACCAAAGAATTGCTTACAAAACTGTAAAACTAGAGTATTAGAGAGGATAGAAGAATTCCTGATGTAGGGGTGAAATCCGTAGATATCAGGAGGAATATCGAAGGCGAAAGCATCTGTCCGGCTAAATACTGACGCTGTTGCACGAGAGTGTGGGGAGCAAACAGG

Kingdom	Phylum	Class	Order	Family	Genus	Blank Total (n=1)	Sample Total (n=56)	Amplicon Sequence Variants (ASVs)
Bacteria	Proteobacteria	Alphaproteobacteria	SAR11_clade	Clade_II	Clade_II	26	0	TACGAAGGGACCTAGCGTAGTTCGGAATTACTGGGCTTAAAGAGCTCGTAGGTGGTTAAAAAAGTTGATTGTGAAATCCCAAGGCTCAACCTTGGAACTGCAATCAAAACTTTTTAGCTAGAGTGTGACAGGGGAAAGTGGAATTTCTAGTGTAGAGGTGAAATTCGTAGATATTAGAAAGAACACCAAAAGCGAAGGCAACTTTCTGGGTCACTACTGACACTGAGGAGCGAAAGCATGGGTAGCGAAGAGG
Bacteria	Proteobacteria	Deltaproteobacteria	Oligoflexales	Oligoflexaceae	Oligoflexaceae	26	0	TACGAAAGGTGCAAGCGTTGTTCGGAATCATTGGGCGTAAAGCGCACGTAGGCGGGGACATATGTCGATTGTGAAAGCCCAGGGCTCAACCTTGGAATTGCAGTCGAAACTGTGTCTCTTGAATATCTGAGAGGGTGGGGGAATTCCTGGTGAAGCAGTGAAATGCGTAGATATCAGGAGGAACACCGGAGGCGAAGGCGCCTGCCTGGCAGAATATTGACGTTGAGGTGCGAAAGCGTGGGGAGCAAACAGG
Bacteria	Bacteroidetes	Bacteroidia	Bacteroidia	Bacteroidia	Bacteroidia	27	0	TACGGAGGGTGCAAGCGTTATCCGGATTCATTGGGTTTAAAGGGTGCGTAGGCGGAATGATAAGTCAGTGGTGAAATCCCATAGCTCAACTATGGAACTGCCATTGATACTGTCGTTCTTGAGTCTGGCTGAGGTTGGCGGAATGTGGCATGTAGCGGTGAAATGCTTAGATATGCCACAGAACACCGATCGCGAAGGCAGCTAACCAAACCAGTACTGACGCTGAGGCACGAAAGCGTGGGGAGCAAACAGG
Bacteria	Cyanobacteria	Oxyphotobacteria	Oxyphotobacteria	Oxyphotobacteria	Oxyphotobacteria	27	0	TACGGAGGATGCAAGCGTTATCCGGAATTATTGGGCGTAAAGGGTCCGCAGGTGGCCGTTCAAGTCAGTTGTTAAAGGCTGGGGCTTAACTCCGGACGAGCAATTGAAACTGAAGGGCTAGAGTACGGTAGGGGTAGAGGGAATTCCTAGTGTAGCGGTGAAATGCGTAGATATTAGGAAGAACACCAGTGGCGAAGGCGCTCTACTGGGCCTGTACTGACACTGAGGGACGAAAGCTAGGGGAGCGAAAGGG

Kingdom	Phylum	Class	Order	Family	Genus	Blank Total (n=1)	Sample Total (n=56)	Amplicon Sequence Variants (ASVs)
Bacteria	Gemmatimonadetes	BD2-11_terrestrial_group	BD2-11_terrestrial_group	BD2-11_terrestrial_group	BD2-11_terrestrial_group	27	0	GACGGAGGGCGCAAGCGTTGTTCGGAATTACTGGGCGTAAAGCGCACGCAGGCGGTTTGCTTAGTCCTTTGTGAAAGCCCGGGGCTCAACCCCGGAATTGCATGGGATACTGGCAGACTGGAGACAGGTAGAGGCTAGCGGAATTCCTGGTGTAGCGGTGGAATGCGTAGATATCAGGAAGAACACCTGTGGCGAAGGCGTCTAGCTGGGCCTGTTCTGACGCTCATGTGCGAAAGCGTGGGGAGCAAACAGG
Bacteria	Proteobacteria	Gammaproteobacteria	SAR86_clade	SAR86_clade	SAR86_clade	28	84	TACGGAGGGTCCAAGCGTTAATCGGAATTACTGGGCGTAAAGCGCGCGTAGGCGGTACGCTAAGTTGGGTGTGAAATCCCCGGGCTTAACCTGGGAACTGCATTCAAAACTTGTGTACTGGAGTACGAAAGAGGCGAGTAGAATTCATGGTGTAGCGGTGAAATGCGTAGATATCATGAGGAATACCAATGGCGAAGGCAACTCGCTGGTTCGTAACTGACGCTGAGGTGCGAAAGCGTGGGTAGCAAACAGG
Bacteria	Proteobacteria	Alphaproteobacteria	Puniceispirillales	SAR116_clade	SAR116_clade	29	310	TACGAAGGGGGCGAGCGTTATTCGGAATTACTGGGCGTAAAGGGAGCGCAGGCGGTTCATTTAGTCAGACGTGAAAGCCCCGGGCTCAACCCGGGAGGTGCGTTTGATACTGGTGAGCTAGAAATCAGAAGAGGATAGTGGAATTCCCAGTGTAGAGGTGAAATTCGTAGATATTGGGAAGAACACCAGTGGCGAAAGCGGCTATCTGGTCTGTATTTGACGCTGAGGCTCGAAAGCGTGGGGAGCAAACAGG
Bacteria	Proteobacteria	Gammaproteobacteria	SAR86_clade	SAR86_clade	SAR86_clade	29	29	TACGGAGGGTCCAAGCGTTAATCGGAATTACTGGGCGTAAAGCGCGCGTAGGCGGTTTGACAAGTTGGGTGTGAAATCCCCGGGCTTAACCTGGGAACTGCATTCAAAACTTTTAAACTGGAGTACGGGAGAGGAGAGTAGAATTCATGGTGTAGCGGTGAAATGCGTAGATATCATGAGGAATACCAATGGCGAAGGCAACTCTCTGGACCGTAACTGACGCTGAGGTGCGAAAGCGTGGGTAGCAAACAGG

Kingdom	Phylum	Class	Order	Family	Genus	Blank Total (n=1)	Sample Total (n=56)	Amplicon Sequence Variants (ASVs)
Bacteria	Proteobacteria	Alphaproteobacteria	Rhodobacterales	Rhodobacteraceae	Shimia	30	6425	TACGGAGGGGGTTAGCGTTGTTCGGAATTACTGGGCGTAAAGCGCGCGTAGGCGGGTTGTTAAGTTAGAGGTGAAATCCCGGGGCTCAACCCCGGAACTGCCTTTAATACTGGCAACCTAGAGTTCGAGAGAGGTGAGTGGAATTCCGAGTGTAGAGGTGAAATTCGTAGATATTCGGAGGAACACCAGTGGCGAAGGCGGCTCACTGGCTCGATACTGACGCTGAGGTGCGAAAGTGTGGGGAGCAAACAGG
Bacteria	Epsilonbacteraeota	Campylobacteria	Campylobacterales	Arcobacteraceae	Arcobacter	30	590	TACGGAGGGCGCAAGCGTTACTCGGAATCACTGGGCGTAAAGAGCGTGTAGGCGGGTTATTAAGTCAGATGTGAAAGCCTGTGGCTCAACCATAGAACTGCATTTGAAACTGATAACCTAGAGTATGGGAGAGGTAGATGGAATTTCTGGTGTAGGGGTAAAATCCGTAGAGATCAGAAGGAATACCGAGTGCGAAGGCGATCTACTGGAACATTACTGACGCTGAGACGCGAAAGCGTGGGGAGCAAACAGG
Bacteria	Verrucomicrobia	Verrucomicrobiae	Verrucomicrobiales	Verrucomicrobiales	Verrucomicrobiales	31	73	TACGAAGGTCCCAAGCGTTATTCGGAATCACTGGGCGTAAAGGGAGCGTAGGCGGCGTGGAAAGTCAGATGTGAAATCCCGGGGCTCAACCCTGGAACTGCATCCGATACTCCCATGCTAGAGGACTGGAGAGGTGTTTGGAATTTTCGGTGTAGCAGTGAAATGCGTAGAGATCGAAAGGAAGACCAATGGCGAAGGCAGAACACTGGACAGTACCTGACGCTGAGGCTCGAAGGCTAGGGGAGCGAAAGGG
Bacteria	Proteobacteria	Gammaproteobacteria	Gammaproteobacteria	Gammaproteobacteria	Gammaproteobacteria	31	0	TACGGAGGGTGCGAGCGTTAATCGGAATTACTGGGCGTAAAGCGCATGCAGGCGGCTTGTTAAGCCAGATGTGAAAGCCCGGGGCTCAACCTCGGAATAGCATTTGGAACTGGCAGGCTAGAGTCTTGTAGAGGGGGGTAGAATTTCAGGTGTAGCGGTGAAATGCGTAGATATGTGGAGGAATACCGGTGGCGAAGGCGGCCTCCTGGCTCGACACTGACGCTGAGGTGCGAAAGCGTGGGGAGCAAACAGG

Kingdom	Phylum	Class	Order	Family	Genus	Blank Total (n=1)	Sample Total (n=56)	Amplicon Sequence Variants (ASVs)
Bacteria	Proteobacteria	Gammaproteobacteria	Alteromonadales	Alteromonadaceae	Aestuariibacter	32	9774	TACGGAGGGTGCGAGCGTTAATCGGAATTACTGGGCGTAAAGCGCACGCAGGCGGATTGTTAAGCTAGAGGTGAAAGCCCCGCGCTCAACGTGGGAATTGCCTTTAGAACTGGCAGTCTAGAGTCTTGGAGAGGGGAGTGGAATTCCAGGTGTAGCGGTGAAATGCGTAGAGATCTGGAGGAACATCAGTGGCGAAGGCGACTCCCTGGCCAAAGACTGACGCTCATGTGCGAAAGTGTGGGTAGCGAACAGG
Bacteria	Proteobacteria	Gammaproteobacteria	Vibrionales	Vibrionaceae	Vibrio	32	3537	TACGGAGGGTGCGAGCGTTAATCGGAATTACTGGGCGTAAAGCGCATGCAGGTGGTTAATTAAGTCAGATGTGAAAGCCCGGGGCTCAACCTCGGAAGGCCATTTGAAACTGGTTAACTAGAGTACTGTAGAGGGGGGTAGAATTTCAGGTGTAGCGGTGAAATGCGTAGAGATCTGAAGGAATACCGGTGGCGAAGGCGGCCCCCTGGACAGATACTGACACTCAGATGCGAAAGCGTGGGGAGCAAACAGG
Bacteria	Proteobacteria	Gammaproteobacteria	Alteromonadales	Alteromonadaceae	Alteromonadaceae	32	1168	TACGGAGGGTGCGAGCGTTAATCGGAATTACTGGGCGTAAAGCGCACGCAGGCGGTTTGTTAAGCAAGATGTGAAAGCCCCGGGCTCAACCTGGGAACTGCATTTTGAACTGGCAGACTAGAGTCTTGGAGAGGGGGGTGGAATTCCAGGTGTAGCGGTGAAATGCGTAGAGATCTGGAGGAACATCAGTGGCGAAGGCGGCCCCCTGGCCAAAGACTGACGCTCATGTGCGAAAGTGTGGGTAGCGAACAGG
Bacteria	Proteobacteria	Gammaproteobacteria	Oceanospirillales	Saccharospirillaceae	Thalassolituus	32	581	TACGGAGGGTGCAAGCGTTAATCGGAATTACTGGGCGTAAAGCGCGCGTAGGTGGCTTGTTAAGCGGAATGTGAAAGCCCCGGGCTCAACCTGGGAACTGCATTGCGAACTGGCAAGCTAGAGTACAGTAGAGGGTAGTGGAATTTCCTGTGTAGCGGTGAAATGCGTAGAGATGGGAAGGAACATCAGTGGCGAAGGCGACTGCCTGGACTGATACTGACACTGAGGTGCGAAAGCGTGGGGAGCAAACAGG

Kingdom	Phylum	Class	Order	Family	Genus	Blank Total (n=1)	Sample Total (n=56)	Amplicon Sequence Variants (ASVs)
Bacteria	Proteobacteria	Alphaproteobacteria	Rhodobacterales	Rhodobacteraceae	Rhodobacteraceae	32	0	TACGGAGGGGGTTAGCGTTGTTCGGAATTACTGGGCGTAAAGCGCACGTAGGCGGATTAATAAGTCAGGGGTGAAATCCCGAGGCTCAACCTCGGAACTGCCTTTGATACTGTTAGTCTTGAGTTCGAGAGAGGTGAGTGGAATTCCGAGTGTAGAGGTGAAATTCGTAGATATTCGGAGGAACACCAGTGGCGAAGGCGGCTCACTGGCTCGATACTGACGCTGAGGTGCGAAAGCGTGGGGAGCAAACAGG
Bacteria	Proteobacteria	Alphaproteobacteria	Alphaproteobacteria	Alphaproteobacteria	Alphaproteobacteria	32	0	TACGGAGGGGGTTAGCGTTGTTCGGAATTACTGGGCGTAAAGCGCGCGTAGGCGGACTGCCAAGTCAGGGGTGAAATCCCGGGGCTTAACCCCGGAACTGCCCTTGAGACTGGTAATCTAGAGTTCGGGAGAGGTAAGTGGAATTCCTAGTGTAGAGGTGAAATTCGTAGATATTAGGAAGAACACCAGTGGCGAAGGCGGCTTACTGGACCGATACTGACGCTGAGGTGCGAAAGCGTGGGGAGCAAACAGG
Bacteria	Proteobacteria	Gammaproteobacteria	Vibrionales	Vibrionaceae	Photobacterium	33	10330	TACGGAGGGTGCGAGCGTTAATCGGAATTACTGGGCGTAAAGCGCATGCAGGCGGCTTGTTAAGCCAGATGTGAAAGCCCGGGGCTCAACCTCGGAATAGCATTTGGAACTGGCAGGCTAGAGTCTTGTAGAGGGGGGTAGAATTTCAGGTGTAGCGGTGAAATGCGTAGAGATCTGAAGGAATACCGGTGGCGAAGGCGGCCCCCTGGACAAAGACTGACGCTCAGATGCGAAAGCGTGGGGAGCAAACAGG
Bacteria	Proteobacteria	Alphaproteobacteria	Rhodobacterales	Rhodobacteraceae	Pseudooceanicola	33	1469	TACGGAGGGGGTTAGCGTTGTTCGGAATTACTGGGCGTAAAGCGCGCGTAGGCGGACTATTAAGTCAGGGGTGAAATCCCGGGGCTCAACCCCGGAACTGCCTTTGATACTGGTAGTCTTGAGTTCGAGAGAGGTGAGTGGAATTCCGAGTGTAGAGGTGAAATTCGTAGATATTCGGAGGAACACCAGTGGCGAAGGCGGCTCACTGGCTCGATACTGACGCTGAGGTGCGAAAGTGTGGGGAGCAAACAGG

Kingdom	Phylum	Class	Order	Family	Genus	Blank Total (n=1)	Sample Total (n=56)	Amplicon Sequence Variants (ASVs)
Bacteria	Proteobacteria	Alphaproteobacteria	Rickettsiales	S25-593	S25-593	33	143	TACGGAGGGAGCTAGCGTTGTTCGGAATTACTGGGCGTAAAGAGCGTGTAGGCTGTTAGGTAAGTCAGAGGTGAAATCCCTAAGCTCAACTTAGGAACTGCCTTTGATACTATCTTACTAGAGATCGAGAGAAGTTGGCGGAATTCCAAGTGTAGAGGTGAAATTCGTAGATATTTGGAAGAACACCAGTGGCGAAAGCGGCCAACTGGCTCGTTTCTGACGCTAAGACGCGAAAGCGTGGGTAGCAAACAGG
Bacteria	Proteobacteria	Gammaproteobacteria	SAR86_clade	SAR86_clade	SAR86_clade	33	110	TACGGAGGGTCCAAGCGTTAATCGGAATTACTGGGCGTAAAGCGCGCGTAGGCGGTTTAGCAAGTTGTATGTGAAAGCCCTGGGCTCAACCTAGGAACTGCATTCAAAACTACTAAGCTAGAGTACGAGAGAGGAGAGTAGAATTTCTGGTGTAGCGGTGAAATGCGTAGATATCAGAAGGAATACCAATGGCGAAGGCAGCTCTCTGGCTCGTCACTGACGCTGAGGTGCGAAAGCGTGGGTAGCAAACAGG
Bacteria	Proteobacteria	Gammaproteobacteria	Oceanospirillales	Saccharospirillaceae	Reinekea	34	902	TACGGAGGGTGCAAGCGTTAATCGGAATTACTGGGCGTAAAGCGCGCGTAGGCGGTTTGTTAAGTTGGATGTGAAAGCCCCGGGCTCAACCTGGGAACTGCATTCAAAACTGGCAAACTGGAGTACGGCAGAGGCAAGTGGAATTTCCAGTGTAGCGGTGAAATGCGTAGAGATTGGAAGGAACACCAGTGGCGAAGGCGACTTGCTGGGCTGATACTGACGCTGAGGTGCGAAAGCGTGGGGAGCAAACAGG
Bacteria	Proteobacteria	Gammaproteobacteria	KI89A_clade	KI89A_clade	KI89A_clade	34	27	TACGGAGGGTACGAACGTTAATCGGAATTACTGGGCGTAAAGCGCGCGTAGGCGGTTTGATAAGTGGGATGTGAAAGCCCCGGGCTCAACCTGGGAACTGCATTCCAAACTGTCAGACTAGAGTGTGGTAGAGGGAGGTAGAATTTCCTGTGTAGCGGTGAAATGCGTAGATATAGGAAGGAATACCGATGGCGAAGGCAGCCTCCTGGACCAACACTGACGCTGAGGTGCGAAAGCGTGGGGAGCAAACAGG

Kingdom	Phylum	Class	Order	Family	Genus	Blank Total (n=1)	Sample Total (n=56)	Amplicon Sequence Variants (ASVs)
Bacteria	Proteobacteria	Gammaproteobacteria	Alteromonadales	Pseudoalteromonadaceae	Psychrosphaera	35	192	TACGGAGGGTGCGAGCGTTAATCGGAATTACTGGGCGTAAAGCGTACGCAGGCGGCCATTTAAGTCAGATGTGAAAGCCCCGGGCTCAACCTGGGAACTGCATTTGAAACTGGATGGCTAGAGTGTGATAGAGGGTGGTAGAATTTCAGGTGTAGCGGTGAAATGCGTAGAGATCTGAAGGAATACCGATGGCGAAGGCAGCCACCTGGGTCAACACTGACGCTCATGTACGAAAGCGTGGGTAGCAAACAGG
Bacteria	Proteobacteria	Gammaproteobacteria	Cellvibrionales	Halieaceae	Pseudohaliea	36	0	TACGGAGGGTGCAAGCGTTAATCGGAATTACTGGGCGTAAAGCGCGCGTAGGCGGTTTGTTAAGTCGGATGTGAAAGCCCCGGGCTCAACCTGGGAATTGCACCCGATACTGGCCGACTGGAGTGCGAGAGAGGGAGGTAGAATTCCACGTGTAGCGGTGAAATGCGTAGATATGTGGAGGAATACCGGTGGCGAAGGCGGCCTCCTGGCTCGACACTGACGCTGAGGTGCGAAAGCGTGGGGAGCAAACAGG
Bacteria	Bacteroidetes	Bacteroidia	Flavobacteriales	Flavobacteriaceae	Aquibacter	37	240	TACGGAGGATCCAAGCGTTATCCGGAATCATTGGGTTTAAAGGGTCCGTAGGTGGATTAGTAAGTCAGAGGTGAAAGCCTGCAGCTCAACTGTAGAACTGCCTTTGATACTGCTAGTCTTGAGTCATTATGAAGTGGTTAGAATGTGTAGTGTAGCGGTGAAATGCATAGATATTACACAGAATACCGATTGCGAAGGCAGATCACTAATAATGTACTGACACTGATGGACGAAAGCGTGGGGAGCGAACAGG
Bacteria	Proteobacteria	Alphaproteobacteria	Micavibrionales	Micavibrionaceae	Micavibrionaceae	38	9	TACGAATGGGGCGAGCGTTGTTCGGAATCACTGGGCGTAAAGCGTATGTAGGCGGAATGGAAAGTCAGAAGTGAAATCCCGGGGCTCAACCCCGGAACTGCTTTTGAAACTTCCATTCTAGAGTTCTGGAGAGGCAAGGGGAATTCCGAGTGTAGAGGTGAAATTCGCAGATATTCGGAGGAACACCAGTGGCGTAGGCGCCTTGCTGGACAGATACTGACGCTGAGATACGAAAGCGTGGGGAGCAAACAGG

Kingdom	Phylum	Class	Order	Family	Genus	Blank Total (n=1)	Sample Total (n=56)	Amplicon Sequence Variants (ASVs)
Bacteria	Proteobacteria	Gammaproteobacteria	Alteromonadales	Alteromonadaceae	Agaribacter	38	0	TACGGAGGGTGCGAGCGTTAATCGGAATTACTGGGCGTAAAGCGCACGCAGGCGGTTATTTAAGCTAGAAGTGAAAGCCCTGGGCTCAACCTGGGAAGGCCTTTTAGAACTGGATGACTAGAGTCTTGGAGAGGGGAGTGGAATTCCACGTGTAGCGGTGAAATGCGTAGATATGTGGAGGAACATCAGTGGCGAAGGCGACTCCCTGGCCAAAGACTGACGCTCATGTGCGAAAGTGTGGGTAGCGAACAGG
Bacteria	Proteobacteria	Alphaproteobacteria	Rhizobiales	Beijerinckiaceae	Methylobacterium	39	4	TACGAAGGGGGCTAGCGTTGCTCGGAATCACTGGGCGTAAAGGGCGCGTAGGCGGCCATTCAAGTCGGGGGTGAAAGCCTGTGGCTCAACCACAGAATTGCCTTCGATACTGTTTGGCTTGAGTCTGGTAGAGGTTGGTGGAACTGCGAGTGTAGAGGTGAAATTCGTAGATATTCGCAAGAACACCAGTGGCGAAGGCGGCCAACTGGACCAGTACTGACGCTGAGGCGCGAAAGCGTGGGGAGCAAACAGG
Bacteria	Actinobacteria	Actinobacteria	Micrococcales	Micrococcaceae	Renibacterium	41	585	TACGTAGGGTGCAAGCGTTATCCGGAATTATTGGGCGTAAAGAGCTCGTAGGCGGTTTGTCGCGTCTGTCGTGAAAGTCCGGGGCTCAACTCCGGATCTGCGGTGGGTACGGGCAGACTAGAGTGATGTAGGGGAGACTGGAATTCCTGGTGTAGCGGTGGAATGCGCAGATATCAGGAGGAACACCGATGGCGAAGGCAGGTCTCTGGGCATTAACTGACGCTGAGGAGCGAAAGCATGGGGAGCGAACAGG
Bacteria	Proteobacteria	Gammaproteobacteria	Alteromonadales	Alteromonadaceae	Alteromonas	41	0	TACGGAGGGTGCGGGCGTTAATCGGAATTACTGGGCGTAAAGCGCACGCAGGCGGTTTGTTAAGCTAGATGTGAAAGCCCCGGGCTCAACCTGGGATGGTCATTTAGAACTGGCAGACTAGAGTCTTGGAGAGGGGAGTGGAATTCCAGGTGTAGCGGTGAAATGCGTAGATATCTGGAGGAACATCAGTGGCGAAGGCGACTCCCTGGCCAAAGACTGACGCTCATGTGCGAAAGTGTGGGTAGCGAACAGG

Kingdom	Phylum	Class	Order	Family	Genus	Blank Total (n=1)	Sample Total (n=56)	Amplicon Sequence Variants (ASVs)
Bacteria	Proteobacteria	Gammaproteobacteria	Oceanospirillales	Endozoicomonadaceae	Endozoicomonas	42	68	TACGGAGGGTGCGAGCGTTAATCGGAATTACTGGGCGTAAAGCGTGCGTAGGCGGCCTTTTAAGTTGAATGTGAAAGCCCCGGGCTCAACCTGGGAACGGCATCCAAAACTGAGAGGCTCGAGTGCGGAAGAGGAGTGTGGAATTTCCTGTGTAGCGGTGAAATGCGTAGATATAGGAAAGAACACCAGTGGCGAAGGCGACACTCTGGTCTGACACTGACGCTGAGGTACGAAAGCGTGGGGAGCAAACAGG
Bacteria	Proteobacteria	Alphaproteobacteria	Puniceispirillales	SAR116_clade	SAR116_clade	42	0	TACGAAGGGGGCGAGCGTTGTTCGGAATTACTGGGCGTAAAGGGAGCGCAGGCGGTTCATTCAGTTAGGCGTGAAAGCCCCGGGCTCAACCTGGGAACTGCGCTTAATACTGATGAGCTAGAAATCAGAAGAGGATAGTGGAATTCCCAGTGTAGAGGTGAAATTCGTAGATATTGGGAAGAACACCAGTGGCGAAAGCGGCTATCTGGTCTGAATTTGACGCTGAGGCTCGAAAGCGTGGGGAGCAAACAGG
Bacteria	Actinobacteria	Acidimicrobiia	Actinomarinales	Actinomarinaceae	Candidatus_Actinomarina	44	3873	TACGTAGGGGGCGAGCGTTGTCCGGATTTATTGGGCGTAAAGAGCTCGTAGGCGGTTCAACAAGTCGGTCGTAAAAGTTCAGGGCTCAACCCTGAAATGTCGATCGATACTGTTGTGACTAGGATACGGTAGAGGTGAGTGGAATTCCGAGTGTAGCGGTGAAATGCGTAGATATTCGGAGGAACACCAATTGCGAAGGCAGCTCACTGGGCCGCTATCGACGCTGAGGAGCGAAAGCTAGGGGAGCAAACAGG
Bacteria	Cyanobacteria	Oxyphotobacteria	Nostocales	Phormidiaceae	Phormidiaceae	44	196	TACGGAGGAGGCAAGCGTTATCCGGAATGATTGGGCGTAAAGCGTCTGTAGGTGGCTATTCAAGTCTGCTGTCAAAGCCTGAGGCTCAACCTCAGATCGGCAGTGGAAACTGAGTAGCTTGAGTGCGGTAGGGGCAGAGGGAATTCCCGGTGTAGCGGTGAAATGCGTAGAGATCGGGAAGAACACCGGTGGCGAAAGCGCTCTGCTGGGCCGCAACTGACACTGAGGGACGAAAGCTAGGGGAGCGAATGGG

Kingdom	Phylum	Class	Order	Family	Genus	Blank Total (n=1)	Sample Total (n=56)	Amplicon Sequence Variants (ASVs)
Bacteria	Bacteroidetes	Bacteroidia	Flavobacteriales	Flavobacteriaceae	Winogradskyella	46	0	TACGGAGGATGCAAGCGTTATCCGGAATCATTGGGTTTAAAGGGTCCGTAGGTGGATTAATAAGTCAGAGGTGAAAGCCTGCAGCTTAACTGTAGAACTGCCTTTGATACTGTTAATCTTGAATTATTGTGAAGTAGTTAGAATATGTAGTGTAGCGGTGAAATGCATAGATATTACATAGAATACCGATTGCGAAGGCAGATTACTAACAATATATTGACACTGATGGACGAAAGCGTGGGGAGCAAACAGG
Bacteria	Cyanobacteria	Oxyphotobacteria	Synechococcales	Cyanobiaceae	Prochlorococcus_MIT9313	49	34	TACGGGAGTGGCAAGCGTTATCCGGAATTATTGGGCGTAAAGCGTCCGCAGGCGGCTTTTCAAGTCTGCTGTTAAAACGTGGAGCTTAACTCCATCATGGCAGTGGAAACTGAAAGGCTTGAGTATGGTAGGGGCAGAGGGAATTCCCGGTGTAGCGGTGAAATGCGTAGATATCGGGAAGAACACCAGTGGCGAAGGCGCTCTGCTGGGCCATTACTGACGCTCATGGACGAAAGCCAGGGGAGCGAAAGGG
Bacteria	Proteobacteria	Alphaproteobacteria	Rhodospirillales	AEGEAN-169_marine_group	AEGEAN-169_marine_group	52	1658	TACGGAAGGTCCTAGCGTTGTTCGGAATTACTGGGCGTAAAGCGCATGTAGGCGGAACAGAAAGTTAGAAGTGAAATCCCTGGGCTCAACCTAGGAATTGCTTTTAAAACTTCTGTTCTGGAATTCAGGAGAGGAAAATGGAATTTCCAGTGTAGAGGTGAAATTCGTAGATATTGGAAGGAACACCAGTGGCGAAGGCGATTTTCTGGACTGATATTGACGCTGAGATGCGAAGGCATGGGTAGCAAACGGG
Bacteria	Actinobacteria	Actinobacteria	Corynebacteriales	Corynebacteriaceae	Corynebacterium_1	53	127	TACGTAGGGTGCGAGCGTTGTCCGGAATTACTGGGCGTAAAGGGCTCGTAGGTGGTTTGTCGCGTCGTCTGTGAAATTCCGGGGCTTAACTCCGGGCGTGCAGGCGATACGGGCATAACTTGAGTACTGTAGGGGTAACTGGAATTCCTGGTGTAGCGGTGAAATGCGCAGATATCAGGAGGAACACCGATGGCGAAGGCAGGTTACTGGGCAGTTACTGACGCTGAGGAGCGAAAGCATGGGTAGCGAACAGG

Kingdom	Phylum	Class	Order	Family	Genus	Blank Total (n=1)	Sample Total (n=56)	Amplicon Sequence Variants (ASVs)
Bacteria	Proteobacteria	Alphaproteobacteria	SAR11_clade	Clade_II	Clade_II	54	5327	TACGAAGGGACCTAGCGTAGTTCGGAATTACTGGGCTTAAAGAGCTCGTAGGTGGTTAAAAAAGTTGATGGTGAAATCCCAAGGCTCAACCTTGGAACTGCCATCAAAACTTTTTAGCTAGAGTGTGATAGAGGTAAGTGGAATTTCTAGTGTAGAGGTGAAATTCGTAGATATTAGAAAGAACACCAAATGCGAAGGCAACTTACTGGGTCACTACTGACACTGAGGAGCGAAAGCATGGGTAGCGAAGAGG
Bacteria	Proteobacteria	Gammaproteobacteria	Alteromonadales	Pseudoalteromonadaceae	Pseudoalteromonas	55	969	TACGGAGGGTGCGAGCGTTAATCGGAATTACTGGGCGTAAAGCGTACGCAGGCGGTTTGTTAAGCGAGATGTGAAAGCCCCGGGCTCAACCTGGGAACTGCATTTCGAACTGGCAGGCTAGAGTGTGATAGAGGGTGGTAGAATTTCAGGTGTAGCGGTGAAATGCGTAGAGATCTGAAGGAATACCGATGGCGAAGGCAGCCACCTGGGTCAACACTGACGCTCATGTACGAAAGCGTGGGGAGCAAACAGG
Bacteria	Proteobacteria	Alphaproteobacteria	Rhodobacterales	Rhodobacteraceae	Leisingera	57	4331	TACGGAGGGGGTTAGCGTTGTTCGGAATTACTGGGCGTAAAGCGCGCGTAGGCGGATTGGAAAGTTGGGGGTGAAATCCCGGGGCTCAACCCCGGAACTGCCTCCAAAACTCCCAGTCTTGAGTTCGAGAGAGGTGAGTGGAATTCCGAGTGTAGAGGTGAAATTCGTAGATATTCGGAGGAACACCAGTGGCGAAGGCGGCTCACTGGCTCGATACTGACGCTGAGGTGCGAAAGTGTGGGGAGCAAACAGG
Bacteria	Proteobacteria	Alphaproteobacteria	SAR11_clade	Clade_I	Clade_Ib	58	2177	TACGAAGGGACCTAGCGTAGTTCGGAATTACTGGGCTTAAAGAGTTCGTAGGTGGTTAAAAAAGTTGGTGGTGAAATCCCAGAGCTTAACTCTGGAACTGCCATCAAAACTTTTTAGCTAGAGTATGATAGAGGAAAGCAGAATTTCTAGTGTAGAGGTGAAATTCGTAGATATTAGAAAGAATACCAATTGCGAAGGCAGCTTTCTGGATCATTACTGACACTGAGGAACGAAAGCATGGGTAGCGAAGAGG

Kingdom	Phylum	Class	Order	Family	Genus	Blank Total (n=1)	Sample Total (n=56)	Amplicon Sequence Variants (ASVs)
Bacteria	Proteobacteria	Gammaproteobacteria	Alteromonadales	Colwelliaceae	Thalassotalea	59	7215	TACGGAGGGTGCGAGCGTTAATCGGAATTACTGGGCGTAAAGCGTGCGTAGGCGGTTTGATAAGCCAGATGTGAAATCCCGGGCTTAACCTCGGAACTGCATTTGGAACTGTTTGACTAGAGTACTGTAGAGGGTGGTGGAATTTCCAGTGTAGCGGTGAAATGCGTAGAGATTGGAAGGAACATCAGTGGCGAAGGCGGCCACCTGGACAGATACTGACGCTGAGGCACGAAAGCGTGGGGAGCGAACAGG
Bacteria	Proteobacteria	Gammaproteobacteria	Vibrionales	Vibrionaceae	Vibrio	59	756	TACGGAGGGTGCGAGCGTTAATCGGAATTACTGGGCGTAAAGCGCATGCAGGTGGATGATTAAGTCAGATGTGAAAGCCCGGGGCTCAACCTCGGAATAGCATTTGAAACTGGTCATCTAGAGTACTGTAGAGGGGGGTAGAATTTCAGGTGTAGCGGTGAAATGCGTAGAGATCTGAAGGAATACCGGTGGCGAAGGCGGCCCCCTGGACAGATACTGACACTCAGATGCGAAAGCGTGGGGAGCAAACAGG
Bacteria	Proteobacteria	Alphaproteobacteria	Rickettsiales	Midichloriaceae	MD3-55	59	0	TACGAAGGGGGCAAGCGTTACTCGGAATTATTGGGCGTAAAGCGTGCGTAGGCGGTTTTATAAGTTGAAAGTGAAAGCCTTTGGCTCAACCAAAGAATTGCTTACAAAACTGTAAAACTAGAGTATTAGAGAGGATAGAAGAATTCCTGATGTAGGGGTGAAATCCGTAGATATCAGGAGGAATATCGAAGTCGAAAGCATCTGTCTGGCTAAATACTGACGCTGTTGCACGAAAGCGTGGGGAGCAAACAGG
Bacteria	Proteobacteria	Gammaproteobacteria	Vibrionales	Vibrionaceae	Vibrio	60	3710	TACGGAGGGTGCGAGCGTTAATCGGAATTACTGGGCGTAAAGCGCATGCAGGTGGTTTGTTAAGTCAGATGTGAAAGCCCGGGGCTCAACCTCGGAACTGCATTTGAAACTGGCAAACTAGAGTACTGTAGAGGGGGGTAGAATTTCAGGTGTAGCGGTGAAATGCGTAGAGATCTGAAGGAATACCAGTGGCGAAGGCGGCCCCCTGGACAGATACTGACACTCAGATGCGAAAGCGTGGGGAGCAAACAGG

Kingdom	Phylum	Class	Order	Family	Genus	Blank Total (n=1)	Sample Total (n=56)	Amplicon Sequence Variants (ASVs)
Bacteria	Proteobacteria	Gammaproteobacteria	Oceanospirillales	Saccharospirillaceae	Thalassolituus	60	1044	TACGGAGGGTGCAAGCGTTAATCGGAATTACTGGGCGTAAAGCGCGCGTAGGTGGTTTGTTAAGCGGAATGTGAAAGCCCCGGGCTCAACCTGGGAACTGCATTGCGAACTGGCAAGCTAGAGTACAGTAGAGGGTAGTGGAATTTCCTGTGTAGCGGTGAAATGCGTAGAGATGGGAAGGAACATCAGTGGCGAAGGCGACTGCCTGGACTGATACTGACACTGAGGTGCGAAAGCGTGGGGAGCAAACAGG
Bacteria	Verrucomicrobia	Verrucomicrobiae	Verrucomicrobiales	Rubritaleaceae	Rubritalea	61	620	TACGAAGGTCCCGAGCGTTATTCGGAATCACTGGGCGTAAAGGGAGCGTAGGCTGCGCGGTAAGTCAGATGTGAAATCTCAGGGCTCAACCTTGAAACTGCATCCGATACTGCCGTGCTAGAGTAATGGAGAGGTAAGTGGAATTCTCGGTGTAGCAGTGAAATGCGTAGATATCGAGAGGAAGACCAATGGCGAAGGCAGCTTACTGGACATTTACTGACGCTGAGGCTCGAAGGCTAGGGTAGCGAAAGGG
Bacteria	Bacteroidetes	Bacteroidia	Bacteroidia	Bacteroidia	Bacteroidia	61	436	TACGGAGGATTCAAGCGTTATCCGGATTCATTGGGTTTAAAGGGTCTGTAGGCGGATTTATAAGTCAGTGGTGAAATCCCGAGGCTCAACCTCGGAACTGCCATTGAAACTGTAAGTCTTGAGTATAACAGAAGTTGGCGGAATGTGTGGTGTAGCGGTGAAATGCATAGAGATCACACAGAACACCAATTGCGAAGGCAGCTGACTATGTATATACTGACGCTGAGAGACGAAAGCGTGGGGAGCGAACAGG
Bacteria	Proteobacteria	Alphaproteobacteria	Sphingomonadales	Sphingomonadaceae	Erythrobacter	61	170	TACGGAGGGAGCTAGCGTTGTTCGGAATTACTGGGCGTAAAGCGCGCGTAGGCGGCTTTTCAAGTCAGGGGTGAAATCCCGGGGCTCAACCCCGGAACTGCCCTTGAAACTGGATGGCTAGAATACTGGAGAGGTGAGTGGAATTCCGAGTGTAGAGGTGAAATTCGTAGATATTCGGAAGAACACCAGTGGCGAAGGCGACTCACTGGACAGTTATTGACGCTGAGGTGCGAAAGCGTGGGGAGCAAACAGG

Kingdom	Phylum	Class	Order	Family	Genus	Blank Total (n=1)	Sample Total (n=56)	Amplicon Sequence Variants (ASVs)
Bacteria	Proteobacteria	Alphaproteobacteria	Alphaproteobacteria	Alphaproteobacteria	Alphaproteobacteria	62	503	TACGGAGGGGGCGAACGTTATTCGGATTTACTGGGCGTAAAGCGTCTGTAGGCGGTTCAATTAGTTGGATGTGAAAGCCCCGTGCTCAACATGGGAACTGCATCCAATACTGTTGAACTAGAGTATCGGAGAGGAAAGCGGAATATCCAGTGTAGAGGTGAAATTCGTAGATATTGGATGGAACACCAGAGGCGAAGGCGGCTTTCTGGACGATTACTGACGCTGAGAGACGAAAGCGTGGGGAGCAAACAGG
Bacteria	Proteobacteria	Gammaproteobacteria	Francisellales	Francisellaceae	Francisellaceae	62	57	TACGGAGGGTGCGAGCGTTAATCGGAATTACTGGGCGTAAAGGGTTCGTAGGTGGTTAGATCAGTCAGATGTGAAATCCCTGGGCTTAACCTAGGAATTGCATTTGATACTGTTTAACTAGAGTTCACTAGAGGATTGGGGAATTTCCGGTGTAGCGGTGAAATGCGTAGAGATCGGAAGGAACATCAATGGCGAAGGCAACAATCTGGGGTTGAACTGACACTGAGGGACGAAAGCGTGGGTAGCAAACAGG
Bacteria	Cyanobacteria	Oxyphotobacteria	Nostocales	Nostocaceae	Rivularia_PCC-7116	71	426	TACGGAGGATGCAAGCGTTATCCGGAATAATTGGGCGTAAAGCGTTCGTAGGTGGTTTTGTAAGTCTGCTGTTAAAGCGTGTAGCTCAACTACATATAGGCAGTGGAAACTACAAGACTTGAGTGCGTTCGGGGTAGAGGGAATTCCTGGTGTAGCGGTGAAATGCGTAGAGATCAGGAAGAACACCGGTGGCGAAGGCGCTCTACTAGACCGCAACTGACACTGAGGGACGAAAGCTAGGGGAGCGAATGGG
Bacteria	Bacteroidetes	Bacteroidia	Flavobacteriales	Flavobacteriaceae	Tenacibaculum	74	3171	TACGGAGGGTGCAAGCGTTATCCGGAATCATTGGGTTTAAAGGGTCCGCAGGCGGTCAATTAAGTCAGAGGTGAAATCCTACAGCTTAACTGTAGAACTGCCTTTGATACTGGTTGACTTGAGTTATACGGAAGTAGGTAGAATGTGTAGTGTAGCGGTGAAATGCATAGATATTACACAGAATACCGATTGCGAAGGCAGCCTACTACGTATATACTGACGCTCATGGACGAAAGCGTGGGGAGCGAACAGG

Kingdom	Phylum	Class	Order	Family	Genus	Blank Total (n=1)	Sample Total (n=56)	Amplicon Sequence Variants (ASVs)
Bacteria	Lentisphaerae	Lentisphaeria	Lentisphaerales	Lentisphaeraceae	Lentisphaera	74	417	TACGTAGGTGGCGAGCGTTATTCGGATTTACTGGGCGTAAAGGGTCCGTAGGGGGATAAATAAGTTTGATGTGAAATTTCGAGGCTCAACCTCGAACCTGCATTGAAAACTGTTTATCTAGAATTCGGTAGAGGTAAGTGGAATTTGTGGTGTAGCGGTGGAATGCGTAGATATCACAAGGAACATCAAAGGCGAAAGCAGCTTACTGGGCCGATATTGACCCTGAGGGACGAAAGCTAAGGTAGCGAAAAGG
Bacteria	Epsilonbacteraeota	Campylobacteria	Campylobacterales	Arcobacteraceae	Arcobacter	76	0	TACTGAGGGTGCAAGCGTTACTCGGAATCACTGGGCGTAAAGAGCGTGTAGGCGGGTTAATAAGTCAGATGTGAAAGCCTATGGCTCAACCATAGAACTGCATTTGAAACTGTTAACCTAGAATATGGGAGAGGTAGATGGAATTTCTGGTGTAGGGGTAAAATCCGTAGAGATCAGAAGGAATACCGATTGCGAAGGCGATCTACTGGAACATTATTGACGCTGAGACGCGAAAGCGTGGGGAGCAAACAGG
Bacteria	Proteobacteria	Deltaproteobacteria	Desulfovibrionales	Desulfovibrionaceae	Desulfovibrio	77	1154	TACGGAAGGTGCGAGCGTTAATCGGAATCACTGGGCGTAAAGCGCGCGTAGGCGGCCTTTTAAGTCGGACGTGAAAGCCCACGGCTCAACCGTGGAATTGCGCTCGATACTGAGAGGCTTGAGTCCTGGAGGGGGTGGCGGAATTTCGGGTGTAGGAGTGAAATCCGTAGATATCCGAAGGAACACCGGTGGCGAAGGCGGCCACCTGGACAGGTACTGACGCTGAGGTGCGAAAGCGTGGGGAGCAAACAGG
Bacteria	Proteobacteria	Gammaproteobacteria	Oceanospirillales	Saccharospirillaceae	Reinekea	81	502	TACGGAGGGTGCAAGCGTTAATCGGAATTACTGGGCGTAAAGCGCGCGTAGGCGGTTTGTTAAGTTGGATGTGAAAGCCCTGGGCTCAACCTGGGAACTGCATTCAAAACTGGCAAGCTAGAGTACAGCAGAGGCAAGTGGAATTTCAGGTGTAGCGGTGAAATGCGTAGAGATCTGAAGGAACATCAGTGGCGAAGGCGACTTGCTGGGCTGATACTGACGCTGAGGTGCGAAAGCGTGGGGAGCAAACAGG

Kingdom	Phylum	Class	Order	Family	Genus	Blank Total (n=1)	Sample Total (n=56)	Amplicon Sequence Variants (ASVs)
Bacteria	Proteobacteria	Alphaproteobacteria	Alphaproteobacteria	Alphaproteobacteria	Alphaproteobacteria	86	1202	TACGAAGGGGGCAAGCGTTGTTCGGATTTACTGGGCGTAAAGGGCGTGTAGGCGGATGATATAGTTAGAGGTGAAAGCCCAGGGCTCAACCTTGGAACTGCCTTTAATACTTATCATCTAGAGTTTGTGAGAGGGTGGCGGAATATCCAGTGTAGAGGTGAAATTCGTAGATATTGGATAGAATACCAGTGGCGAAGGCGGCCACCTGGCACAACACTGACGCTGAGGCGCGAAAGCGTGGGGAGCAAACAGG
Bacteria	Proteobacteria	Alphaproteobacteria	Alphaproteobacteria	Alphaproteobacteria	Alphaproteobacteria	88	2094	TACGGAGGGGGCTAGCGTTGTTCGGAATCACTGGGCGTAAAGCGCGCGTAGGCGGACTGCCAAGTTGGGGGTGAAAGCCCGGAGCTCAACTCCGGAACGGCCCTCAAAACTGGCAGTCTAGAATCCGGGAGAGGTGAGCGGAATTCCTAGTGTAGAGGTGAAATTCGTAGATATTAGGAAGAACACCAGTGGCGAAGGCGGCTCACTGGACCGGTATTGACGCTGAGGTGCGAAAGCGTGGGGAGCAAACAGG
Bacteria	Bacteroidetes	Bacteroidia	Flavobacteriales	Flavobacteriaceae	NS5_marine_group	89	4872	TACGGAGGATCCAAGCGTTATCCGGAATTATTGGGTTTAAAGGGTCCGCAGGCTGTTTGTTAAGTCAGAGGTGAAATCCTACCGCTCAACGGTAGAACTGCCTTTGATACTGGCAAACTTGAGTTATTGTGAAGTAGTTAGAATGTGTAGTGTAGCGGTGAAATGCATAGATATTACACAGAATACCGATTGCGAAAGCAGATTACTAACAATATACTGACGCTGAGGGACGAAAGCGTGGGTAGCGAACAGG
Bacteria	Proteobacteria	Gammaproteobacteria	Oceanospirillales	Nitrincolaceae	Profundimonas	91	2284	TACGGAGGGTGCGAGCGTTAATCGGAATTACTGGGCGTAAAGCGCGCGTAGGCGGCCAAGTCAGTCAGATGTGAAAGCCCCGGGCTCAACCTGGGAACTGCACCTGATACTGCTTGGCTAGAGTACAGAAGAGGGTGGTGGAATTTCCTGTGTAGCGGTGAAATGCGTAGATATAGGAAGGAACATCAGTGGCGAAGGCGGCCACCTGGTCTGATACTGACGCTGAGGTGCGAAAGCGTGGGGAGCAAACAGG

Kingdom	Phylum	Class	Order	Family	Genus	Blank Total (n=1)	Sample Total (n=56)	Amplicon Sequence Variants (ASVs)
Bacteria	Proteobacteria	Gammaproteobacteria	Alteromonadales	Colwelliaceae	Thalassotalea	94	7570	TACGGAGGGTGCGAGCGTTAATCGGAATTACTGGGCGTAAAGCGTGCGTAGGCGGATAGTTAAGCGAGATGTGAAATCCCGGGGCTCAACCTCGGAACTGCATTTCGAACTGGCTGTCTAGAGTCTTGTAGAGGGTGGTGGAATTTCCAGTGTAGCGGTGAAATGCGTAGAGATTGGAAGGAACATCAGTGGCGAAGGCGGCCACCTGGACAAAGACTGACGCTGAGGCACGAAAGCGTGGGGAGCGAACAGG
Bacteria	Bacteroidetes	Bacteroidia	Flavobacteriales	Flavobacteriaceae	Aquibacter	96	1587	TACGGAGGATCCAAGCGTTATCCGGAATCATTGGGTTTAAAGGGTCCGTAGGTGGATTAGTAAGTCAGAGGTGAAATCCTGCAGCTCAACTGTAGAACTGCCTTTGATACTGCTAGTCTTGAGTCATTATGAAGTGGTTAGAATGTGTAGTGTAGCGGTGAAATGCATAGATATTACACAGAATACCGATTGCGAAGGCAGATCACTAATAATGTACTGACACTGATGGACGAAAGCGTGGGGAGCGAACAGG
Bacteria	Proteobacteria	Alphaproteobacteria	Rhodobacterales	Rhodobacteraceae	Thalassobius	97	4436	TACGGAGGGGGTTAGCGTTGTTCGGAATTACTGGGCGTAAAGCGCGCGTAGGCGGATTAGTAAGTTAGAGGTGAAATCCCGGGGCTCAACCCCGGAACTGCCTTTAATACTGCTAGTCTTGAGTTCGAGAGAGGTGAGTGGAATTCCGAGTGTAGAGGTGAAATTCGTAGATATTCGGAGGAACACCAGTGGCGAAGGCGGCTCACTGGCTCGATACTGACGCTGAGGTGCGAAAGTGTGGGGAGCAAACAGG
Bacteria	Proteobacteria	Alphaproteobacteria	Rhodospirillales	Terasakiellaceae	Terasakiellaceae	97	1030	TACGAAGGGTGCTAGCGTTGTTCGGAATTACTGGGCGTAAAGGGCGCGTAGGCGGACATGTCAGTCAGGGGTGAAATCCCGGGGCTCAACCTCGGAACTGCCTTTGATACTGCATGTCTAGAATATATGAGGGGATAGTGGAATACCAAGTGTAGAGGTGAAATTCGTAGATATTTGGTGGAACACCAGTGGCGAAGGCGACTATCTGGCATATTATTGACGCTGAGGCGCGAAAGCGTGGGGAGCAAACAGG

Kingdom	Phylum	Class	Order	Family	Genus	Blank Total (n=1)	Sample Total (n=56)	Amplicon Sequence Variants (ASVs)
Bacteria	Actinobacteria	Acidimicrobiia	Actinomarinales	Actinomarinaceae	Candidatus_Actinomarina	102	12093	TACGTAGGGGGCGAGCGTTGTCCGGATTTATTGGGCGTAAAGAGCTCGTAGGCGGTTCAACAAGTCGGTCGTGAAAGTTCAGGGCTCAACCCTGAAATGTCGATCGATACTGTTGTGACTAGGATACGGTAGAGGTGAGTGGAATTCCGAGTGTAGCGGTGAAATGCGTAGATATTCGGAGGAACACCAATTGCGAAGGCAGCTCACTGGGCCGCTATCGACGCTGAGGAGCGAAAGCTAGGGGAGCAAACAGG
Bacteria	Proteobacteria	Gammaproteobacteria	Alteromonadales	Alteromonadaceae	Alteromonadaceae	121	4319	TACGGAGGGTGCGAGCGTTAATCGGAATTACTGGGCGTAAAGCGCACGCAGGCGGTTTGTCAAGCTAGATGTGAAAGCCCCGGGCTTAACCTGGGAATTGCATTTAGAACTGTCAGACTAGAGTCATCTAGAGGGGAGTGGAATTCCAGGTGTAGCGGTGAAATGCGTAGAGATCTGGAGGAACATCAGTGGCGAAGGCGACTCCCTGGAGATAGACTGACGCTCATGTGCGAAAGCGTGGGTAGCGAACAGG
Bacteria	Proteobacteria	Gammaproteobacteria	Francisellales	Francisellaceae	Francisellaceae	128	4621	TACGGAGGGTGCGAGCGTTAATCGGAATTACTGGGCGTAAAGGGTTCGTAGGTGGTTAGATCAGTCAGATGTGAAAGCCCTGGGCTTAACCTAGGAATTGCATTTGATACTGTTTAACTAGAGTTCACTAGAGGATTGGGGAATTTCCGGTGTAGCGGTGAAATGCGTAGAGATCGGAAGGAACATCAATGGCGAAGGCAACAATCTGGGGTTGAACTGACACTGAGGGACGAAAGCGTGGGTAGCAAACAGG
Bacteria	Proteobacteria	Gammaproteobacteria	Alteromonadales	Pseudoalteromonadaceae	Algicola	131	1769	TACGGAGGGTGCGAGCGTTAATCGGAATTACTGGGCGTAAAGCGTACGCAGGCGGTCATTTAAGTCAGATGTGAAAGCCCCGGGCTCAACCTGGGAACTGCATTTGAAACTGGATGACTAGAGTGCGGCAGAGGGTGGTAGAATTTCAGGTGTAGCGGTGAAATGCGTAGAGATCTGAAGGAATACCGATGGCGAAGGCAGCCACCTGGGCCGACACTGACGCTCATGTACGAAAGCGTGGGTAGCAAACAGG

Kingdom	Phylum	Class	Order	Family	Genus	Blank Total (n=1)	Sample Total (n=56)	Amplicon Sequence Variants (ASVs)
Bacteria	Proteobacteria	Gammaproteobacteria	Oceanospirillales	Saccharospirillaceae	Oleibacter	140	3326	TACGGAGGGTGCAAGCGTTAATCGGAATTACTGGGCGTAAAGCGCGCGTAGGTTGTTTGCTAAGCGAGATGTGAAAGCCCCGGGCTTAACCTGGGAACTGCATTTCGAACTGGCAAGCTAGAGTACAGTAGAGGGTGGCGGAATTTCCTGTGTAGCGGTGAAATGCGTAGAGATGGGAAGGAACATCAGTGGCGAAGGCGGCCACCTGGACTGATACTGACACTGAGGTGCGAAAGCGTGGGTAGCGAACAGG
Bacteria	Proteobacteria	Gammaproteobacteria	Vibrionales	Vibrionaceae	Vibrio	161	5365	TACGGAGGGTGCGAGCGTTAATCGGAATTACTGGGCGTAAAGCGCATGCAGGTGGTTAGTTAAGTCAGATGTGAAAGCCCGGGGCTCAACCTCGGAACTGCATTTGAAACTGGCTGACTAGAGTACTGTAGAGGGGGGTAGAATTTCAGGTGTAGCGGTGAAATGCGTAGAGATCTGAAGGAATACCGGTGGCGAAGGCGGCCCCCTGGACAGATACTGACACTCAGATGCGAAAGCGTGGGGAGCAAACAGG
Bacteria	Cyanobacteria	Oxyphotobacteria	Synechococcales	Cyanobiaceae	Prochlorococcus_MIT9313	178	2994	TACGGGAGTGGCAAGCGTTATCCGGAATTATTGGGCGTAAAGCGTCCGCAGGCGGCTTTTCAAGTCTGCTGTTAAAGCGTGGAGCTTAACTCCATCATGGCAGTGGAAACTGAAAGGCTTGAGTATGGTAGGGGCAGAGGGAATTCCCGGTGTAGCGGTGAAATGCGTAGATATCGGGAAGAACACCAGTGGCGAAGGCGCTCTGCTGGGCCATTACTGACGCTCATGGACGAAAGCCAGGGGAGCGAAAGGG
Bacteria	Proteobacteria	Gammaproteobacteria	Alteromonadales	Pseudoalteromonadaceae	Algicola	181	3680	TACGGAGGGTGCGAGCGTTAATCGGAATTACTGGGCGTAAAGCGTACGCAGGCGGTCTTATAAGTCAGATGTGAAAGCCCCGGGCTCAACCTGGGAACTGCATTTGAAACTGTAAGACTAGAGTGCGGCAGAGGGTGGTAGAATTTCAGGTGTAGCGGTGAAATGCGTAGAGATCTGAAGGAATACCGATGGCGAAGGCAGCCACCTGGGCCGACACTGACGCTCATGTACGAAAGCGTGGGTAGCAAACAGG

Kingdom	Phylum	Class	Order	Family	Genus	Blank Total (n=1)	Sample Total (n=56)	Amplicon Sequence Variants (ASVs)
Bacteria	Proteobacteria	Alphaproteobacteria	Alphaproteobacteria	Alphaproteobacteria	Alphaproteobacteria	183	5340	TACGGAGGGGGCGAACGTTATTCGGATTTACT<br>GGGCGTAAAGCGTCTGTAGGCGGTTCAATTAG<br>TTGGATGTGAAAGCCCCGTGCTCAACATGGGA<br>ACTGCATTCAATACTGTTGAACTAGAGTATCGG<br>AGAGGAAAGCGGAATATCCAGTGTAGAGGTGA<br>AATTCGTAGATATTGGATGGAACACCAGAGGC<br>GAAGGCGGCTTTCTGGACGATTACTGACGCTG<br>AGAGACGAAAGCGTGGGGAGCAAACAGG
Bacteria	Proteobacteria	Alphaproteobacteria	Rickettsiales	Midichloriaceae	MD3-55	295	1031	TACGAAGGGGGCAAGCGTTACTCGGAATTATT<br>GGGCGTAAAGCGTGCGTAGGCGGTTTTATAAG<br>TTGAAAGTGAAAGCCTTTGGCTCAACCAAAGA<br>ATTGCTTACAAAACTGTAAAACTAGAGTATTA<br>GAGAGGATAGAAGAATTCCTGATGTAGGGGTG<br>AAATCCGTAGATATCAGGAGGAATATCGAAGG<br>CGAAAGCATCTGTCTGGCTAAATACTGACGCT<br>GGTGCACGAAAGCGTGGGGAGCAAACAGG
Bacteria	Proteobacteria	Gammaproteobacteria	Alteromonadales	Pseudoalteromonadaceae	Pseudoalteromonas	376	39787	TACGGAGGGTGCGAGCGTTAATCGGAATTACT<br>GGGCGTAAAGCGTACGCAGGCGGTTTGTTAAG<br>CGAGATGTGAAAGCCCCGGGCTCAACCTGGGA<br>ACTGCATTTCGAACTGGCAAACTAGAGTGTGA<br>TAGAGGGTGGTAGAATTTCAGGTGTAGCGGTG<br>AAATGCGTAGAGATCTGAAGGAATACCGATGG<br>CGAAGGCAGCCACCTGGGTCAACACTGACGCT<br>CATGTACGAAAGCGTGGGGAGCAAACAGG
Bacteria	Epsilonbacteraeota	Campylobacteria	Campylobacterales	Arcobacteraceae	Arcobacter	405	17617	TACGGAGGGTGCAAGCGTTACTCGGAATCACT<br>GGGCGTAAAGAGCGTGTAGGCGGGTTAATAAG<br>TCAGATGTGAAAGCCTATGGCTCAACCATAGA<br>ACTGCATTTGAAACTGTTAACCTAGAATATGG<br>GAGAGGTAGATGGAATTTCTGGTGTAGGGGTA<br>AAATCCGTAGAGATCAGAAGGAATACCGATTG<br>CGAAGGCGATCTACTGGAACATTATTGACGCT<br>GAGACGCGAAAGCGTGGGGAGCAAACAGG

Kingdom	Phylum	Class	Order	Family	Genus	Blank Total (n=1)	Sample Total (n=56)	Amplicon Sequence Variants (ASVs)
Bacteria	Proteobacteria	Alphaproteobacteria	Rickettsiales	Midichloriaceae	MD3-55	577	12032	TACGAAGGGGGCAAGCGTTACTCGGAATTATTGGGCGTAAAGCGTGCGTAGGCGGTTTTATAAGTTGAAAGTGAAAGCCTTTGGCTCAACCAAAGAATTGCTTACAAAACTGTAAAACTAGAGTATTAGAGAAGATAGAAGAATTCCTGATGTAGGGGTGAAATCCGTAGATATCAGGAGGAATATCGAAGGCGAAAGCATCTGTCTGGCTAAATACTGACGCTGTTGCACGAAAGCGTGGGGAGCAAACAGG
Bacteria	Proteobacteria	Gammaproteobacteria	Vibrionales	Vibrionaceae	Catenococcus	635	19707	TACGGAGGGTGCGAGCGTTAATCGGAATTACTGGGCGTAAAGCGCATGCAGGTGGTTTGTTAAGTCAGATGTGAAAGCCCGGGGCTCAACCTCGGAATAGCATTTGAAACTGGCAGACTAGAGTACTGTAGAGGGGGGTAGAATTTCAGGTGTAGCGGTGAAATGCGTAGAGATCTGAAGGAATACCGGTGGCGAAGGCGGCCCCCTGGACAGATACTGACACTCAGATGCGAAAGCGTGGGGAGCAAACAGG
Bacteria	Proteobacteria	Gammaproteobacteria	Vibrionales	Vibrionaceae	Vibrio	857	92604	TACGGAGGGTGCGAGCGTTAATCGGAATTACTGGGCGTAAAGCGCATGCAGGTGGTTTGTTAAGTCAGATGTGAAAGCCCGGGGCTCAACCTCGGAATTGCATTTGAAACTGGCAGACTAGAGTACTGTAGAGGGGGGTAGAATTTCAGGTGTAGCGGTGAAATGCGTAGAGATCTGAAGGAATACCGGTGGCGAAGGCGGCCCCCTGGACAGATACTGACACTCAGATGCGAAAGCGTGGGGAGCAAACAGG
Bacteria	Bacteroidetes	Bacteroidia	Chitinophagales	Saprospiraceae	Saprospira	1396	39426	TACGGAGGGTGCAAGCGTTATCCGGAATCACTGGGTTTAAAGGGTACGTAGGCGGTTCAGTAAGTCAGATGTGAAATGTCGGAGCTCAACTTCGAACTTGCATTTGAAACTGTTGAACTTGAATTAGGTGGAAGTGTGCGGAATGTATCATGTAGCGGTGAAATGCATAGATATGATATAGAACACCAATAGCGAAGGCAGCACACTACGCTTTGATTGACGCTGAGGTACGAAAGCGTGGGGAGCGAACAGG

Kingdom	Phylum	Class	Order	Family	Genus	Blank Total (n=1)	Sample Total (n=56)	Amplicon Sequence Variants (ASVs)
Bacteria	Proteobacteria	Gammaproteobacteria	Oceanospirillales	Saccharospirillaceae	Thalasso lituus	1527	44125	TACGGAGGGTGCAAGCGTTAATCGGAATTACTGGGCGTAAAGCGCGCGTAGGTGGTTTGTTAAGCGGAATGTGAAAGCCCCGGGCTCAACCTGGGAACTGCATTGCGAACTGGCAGACTAGAGTACAGTAGAGGGTAGTGGAATTTCCTGTGTAGCGGTGAAATGCGTAGAGATGGGAAGGAACATCAGTGGCGAAGGCGACTGCCTGGACTGATACTGACACTGAGGTGCGAAAGCGTGGGGAGCAAACAGG
Bacteria	Proteobacteria	Gammaproteobacteria	Oceanospirillales	Endozoicomonadaceae	Endozoicomonas	2093	28638	TACGGAGGGTGCAAGCGTTAATCGGAATTACTGGGCGTAAAGCGTGCGTAGGCGGCCTTTTAAGTTGGATGTGAAAGCCCCGGGCTCAACCTGGGAACGGCATCCAAAACTGAGAGGCTCGAGTGCGGAAGAGGAGTGTGGAATTTCCTGTGTAGCGGTGAAATGCGTAGATATAGGAAAGAACACCAGTGGCGAAGGCGACACTCTGGTCTGACACTGACGCTGAGGTACGAAAGCGTGGGGAGCAAACAGG
Bacteria	Proteobacteria	Gammaproteobacteria	Alteromonadales	Pseudoalteromonadaceae	Algicola	2584	76978	TACGGAGGGTGCGAGCGTTAATCGGAATTACTGGGCGTAAAGCGTACGCAGGCGGTTAGTTAAGTCAGATGTGAAAGCCCCGGGCTCAACCTGGGAACTGCATTTGAAACTGGCTAACTAGAGTGCGGCAGAGGGTGGTAGAATTTCAGGTGTAGCGGTGAAATGCGTAGAGATCTGAAGGAATACCGATGGCGAAGGCAGCCACCTGGGCCGACACTGACGCTCATGTACGAAAGCGTGGGTAGCAAACAGG
Bacteria	Proteobacteria	Alphaproteobacteria	Rickettsiales	Midichloriaceae	MD3-55	4010	40833	TACGAAGGGGGCAAGCGTTACTCGGAATTATTGGGCGTAAAGCGTGCGTAGGCGGTTTTATAAGTTGAAAGTGAAAGCCTTTGGCTCAACCAAAGAATTGCTTACAAAACTGTAAAACTAGAGTATTAGAGAGGATAGAAGAATTCCTGATGTAGGGGTGAAATCCGTAGATATCAGGAGGAATATCGAAGGCGAAAGCATCTGTCTGGCTAAATACTGACGCTGTTGCACGAAAGCGTGGGGAGCAAACAGG

Table S3: Amplicon sequence variants as indicator species for hypoxia.

ASV #	Family	Genus	Correlation Stat	P-value	Sequence
ASV 17	Alteromonadaceae	Alteromonas	0.589	0.0001	TACGGAGGGTGCGAGCGTTAATCGGAATTACTGGGCG TAAAGCGCACGCAGGCGGTTTGTTAAGCTAGATGTGA AAGCCCCGGGCTCAACCTGGGATGGTCATTTAGAACT GGCAGACTAGAGTCTTGGAGAGGGGAGTGGAATTCCA GGTGTAGCGGTGAAATGCGTAGATATCTGGAGGAACA TCAGTGGCGAAGGCGACTCCCTGGCCAAAGACTGACG CTCATGTGCGAAAGTGTGGGTAGCGAACAGG
ASV 256	Nitrincolaceae	Neptuniibacter	0.533	0.0003	TACGGAGGGAGCTAGCGTTGTTCGGAATTACTGGGCG TAAAGCGCACGTAGGCGGCTTTTCAAGTCAGGGGTGA AATCCCGGGGCTCAACCCCGGAACTGCCCTTGAAACT GGATGGCTAGAATACTGGAGAGGTGAGTGGAATTCCG AGTGTAGAGGTGAAATTCGTAGATATTCGGAAGAACA CCAGTGGCGAAGGCGACTCACTGGACAGTTATTGACG CTGAGGTGCGAAAGCGTGGGGAGCAAACAGG
ASV 314	Cellvibrionaceae	Aestuariicella	0.528	0.0002	TACGGAGGGTGCAAGCGTTAATCGGAATTACTGGGCG TAAAGCGCGCGTAGGCGGTTACATAAGTTGGAGGTGA AATCCCCGGGCTCAACCTGGGAACTGCTTTCAAAACTG TGTGACTAGAGTGCAGCAGAGGATAGTGGAATTTCTA GTGTAGCGGTGAAATGCGTAGATATTAGAAGGAACAC CAGTGGCGAAGGCGACTGTCTGGGCTGACACTGACGC TGAGGTGCGAAAGCGTGGGGAGCAAACAGG
ASV 425	Marinobacteraceae	Marinobacter	0.52	0.0003	TACGGAGGGTGCAAGCGTTAATCGGAATTACTGGGCG TAAAGCGCGCGTAGGTGGTTTGGTAAGCGAGATGTGA AAGCCCCGGGCTTAACCTGGGAACGGCATTTCGAACT GTCAGACTAGAGTGTGGTAGAGGGTAGTGGAATTTCC TGTGTAGCGGTGAAATGCGTAGATATAGGAAGGAACA CCAGTGGCGAAGGCGGCTACCTGGACCAACACTGACA CTGAGGTGCGAAAGCGTGGGGAGCAAACAGG

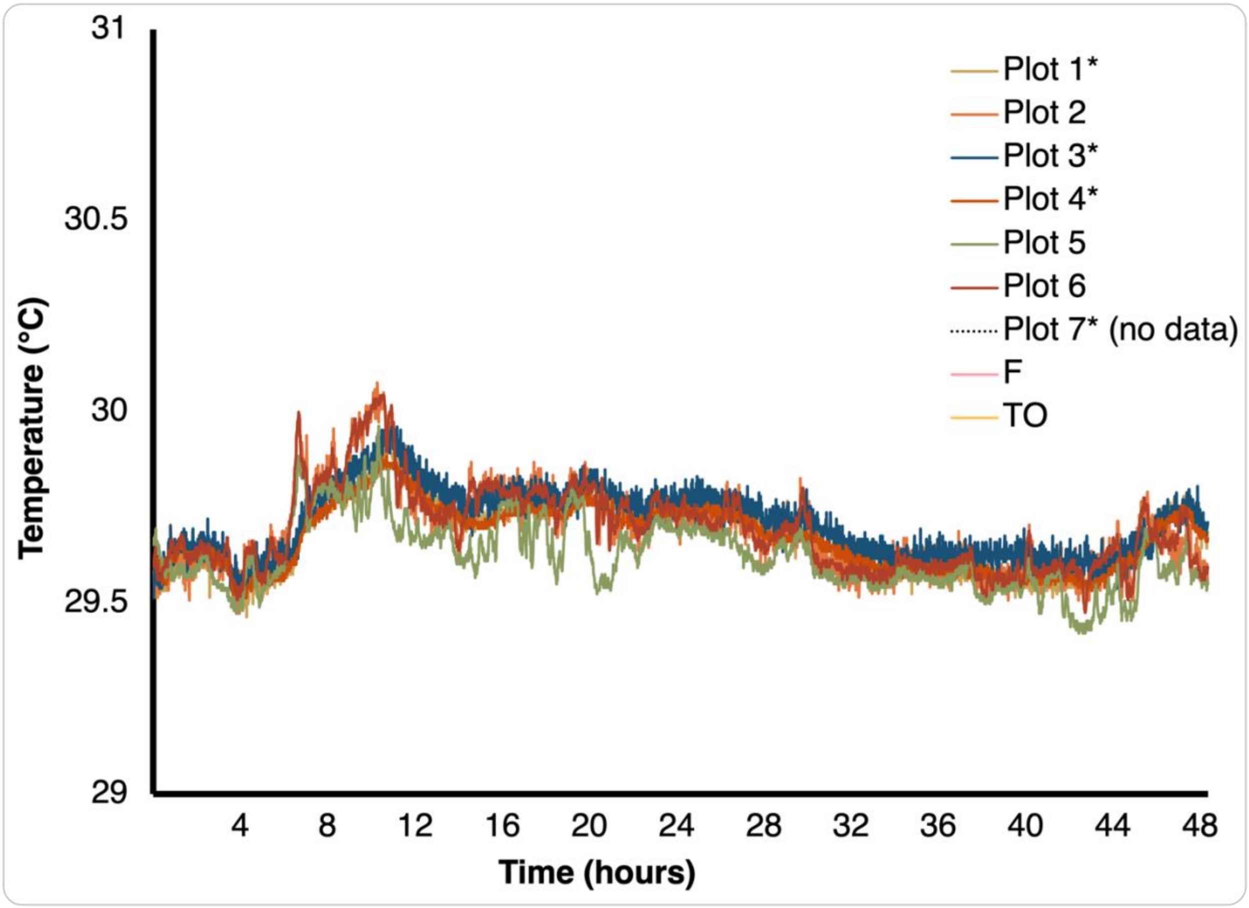

Figure S1: Temperature fluctuations (°C) in the experimental plots in Punta Caracol, Tierra Oscura (TO), and Finca (F) over the course of 48 hours.

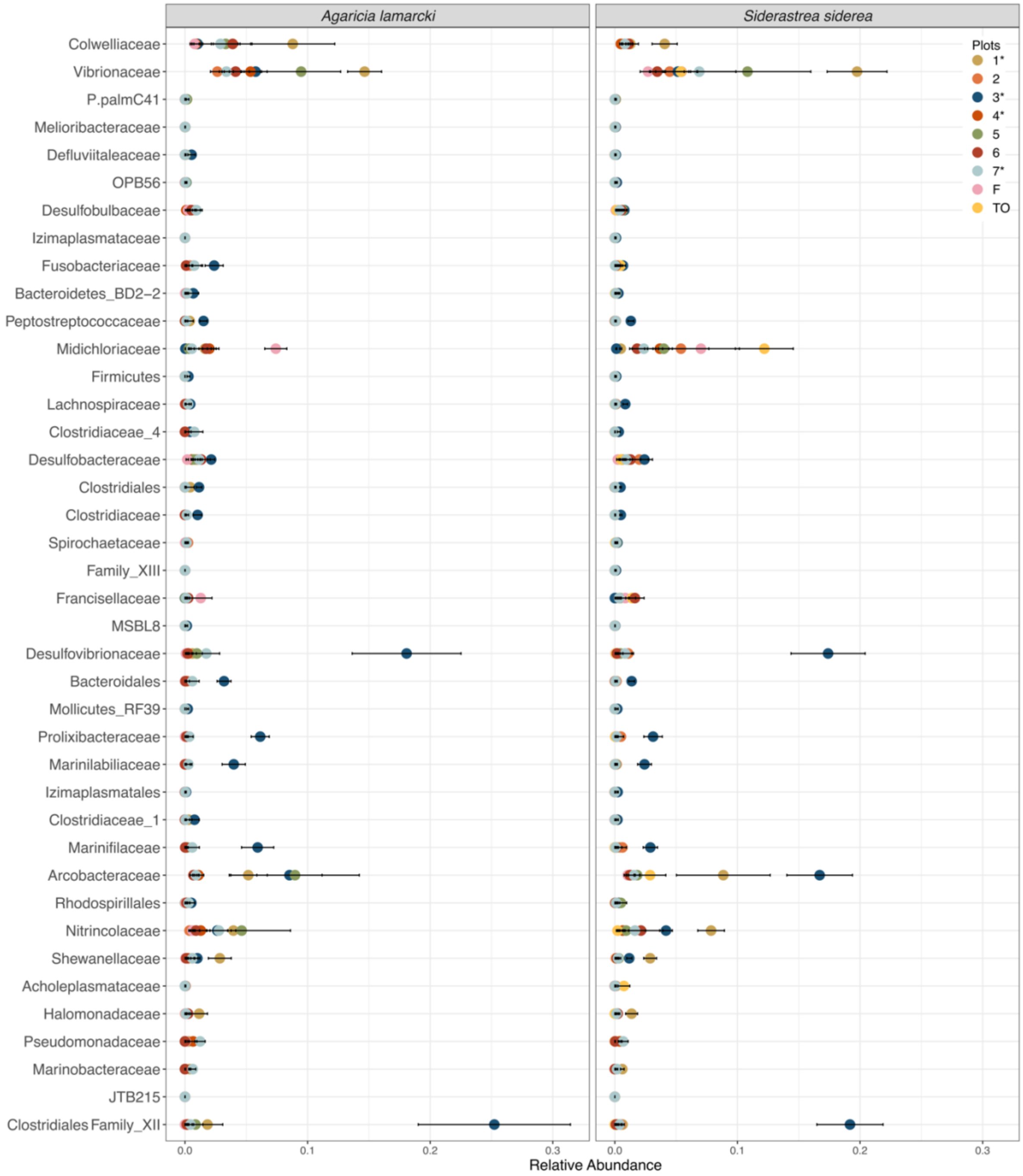

Figure S2: Relative abundance of 40 differentially abundant families across all loggers in Finca (F), Tierra Oscura (TO), control plots and hypoxia plots. Points represent the average relative abundance and error bars depict the standard error from analysis of 56 coral samples. Asterisks next to plot numbers represent hypoxic plots. Notable families that increased significantly in

relative abundance during prolonged, severe hypoxia include *Arcobacteraceae*, *Desulfovibrionaceae*, and *Clostridiales Family XII*.

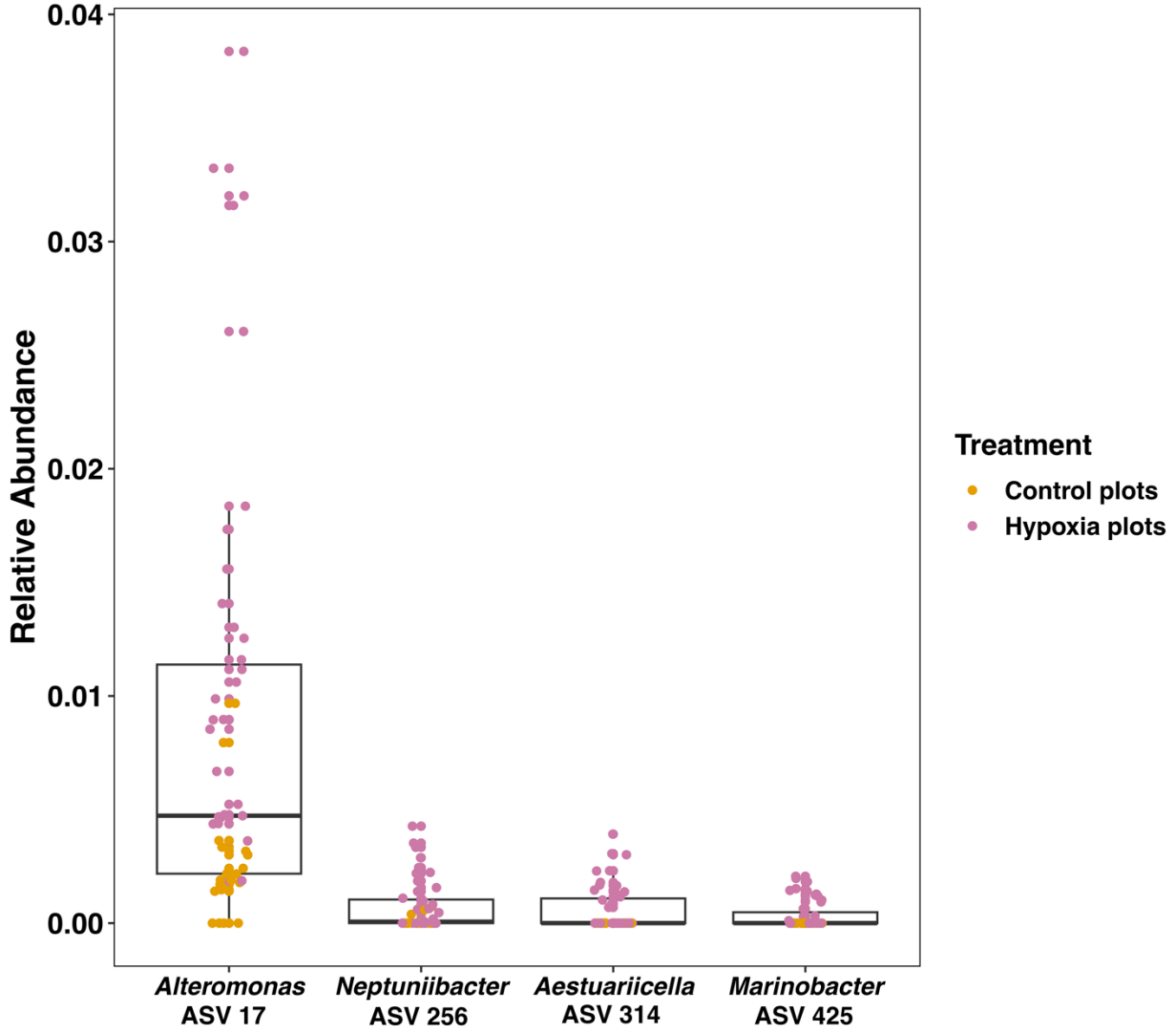

Figure S3: Relative abundance of the four indicator amplicon sequence variants from the genera *Alteromonas*, *Neptuniibacter*, *Aestuariicella*, and *Marinobacter* that were higher in hypoxia plots versus control plots.
